# Chemical programming of kinase inhibitors in a modular chemputer-based system

**DOI:** 10.1038/s42003-026-09873-8

**Published:** 2026-03-27

**Authors:** Hammed A. Badmos, Petrisor-Alin Pirvan, Elena Klimareva, Leroy Cronin, Ross Cagan

**Affiliations:** 1 School of Cancer Sciences, the University of Glasgow, Glasgow, UK; 2 School of Chemistry, the University of Glasgow, University Avenue, Glasgow, UK

**Keywords:** Cancer therapy, Drug screening

## Abstract

Manual synthesis of small molecules can represent a rate-limiting step in medicinal chemistry. This study describes the application of an automated, modular synthesis platform (‘Chemputer’) to a drug discovery project targeting a model of KRAS-mutant colorectal cancer (K-CRC). A 4-anilinoquinazoline-based compound library was synthesized using automated and digitized protocols for nucleophilic aromatic substitution (SnAr) and Suzuki cross-coupling reactions. Chemical synthesis is guided by phenotypic screening of a transgenic *Drosophila* line engineered to model the genetic profile of a patient’s K-CRC tumour. This integrated system enables iterative synthesis and screening cycles. An initial run identified the hit compound AP2-83, which strongly improves animal survival. Kinase profiling and genetic validation find that AP2-83 activity is mediated in part through inhibition of CLK1 and PI3K. A subsequent optimisation effort, informed by these results, produced AP4-43. AP4-43 demonstrates increased efficacy in the *Drosophila* model and greater potency than regorafenib in a mammalian CRC organoid growth assay. Functional analysis indicates AP4-43 acts as a multi-kinase inhibitor, with its enhanced activity associated with the inhibition of a network including CLK1 and NEK4. This work demonstrates the utility of a digital synthesis platform for generating and optimising lead compounds in a complex, preclinical drug discovery context.

## Abstract (web summary)

Chemical programming on a modular Chemputer digital chemistry platform couples automated synthesis with *Drosophila* phenotypic screening, yielding AP4-43, a multi-kinase inhibitor lead that improved survival in a KRAS-mutant colorectal cancer model.

## Introduction

Drug development is a resource-intensive process, with preclinical discovery phases contributing significantly to both cost and timelines^1^. Oncology represents an especially challenging therapeutic area, with typical timelines of 10–20 years, costs exceeding US$1 billion per approved drug, and recent approval rates below 5%^2^.

One rate-limiting step is the synthesis of novel chemical entities, which traditionally relies on manual procedures that can be difficult to scale and reproduce^3,4^. Automated synthesis platforms offer an alternative by translating chemical protocols into standardized, machine-executable code, which can improve reproducibility and accelerate synthetic workflows. The Chemputer is one such modular system that uses a chemical programming language (_*X*_DL) to digitize and automate multi-step synthesis^5^. The application of these platforms to complex biological problems can provide a framework for more efficient discovery cycles.

This study demonstrates the application of a Chemputer medicinal chemistry platform for targeting KRAS-mutant colorectal cancer (K-CRC). K-CRC is often characterized by genetic heterogeneity that contributes to resistance against single-target therapies, making polypharmacological agents—single compounds that engage multiple targets—an area of increasing interest^6,7^. However, identifying effective multi-targeting compounds will require extensive exploration of chemical space and, importantly, functionally tying it to clinically relevant biological models^8,9^.

Here, we report a workflow that integrates the Chemputer’s automated synthesis capabilities with a phenotypic screening platform. We designed and automated a modular, two-step synthesis of a library based on the 4-anilinoquinazoline core scaffold commonly found in kinase inhibitors^10,11^. Using an iterative QSAR-based design, the resulting chemical library was explored using a transgenic *Drosophila* model engineered to express the genetic alterations of a specific K-CRC patient’s tumour, providing a quantitative readout of compound activity in a whole-animal system^7,12,13^. This report details the progression from an initial screening library to the identification and optimisation of a lead compound, AP4-43. The study serves as an example of an integrated, technology-driven workflow for lead compound generation in a preclinical setting. In addition, it generates a compound that, in turn, defines a complex whole animal K-CRC network.

## Results

### Establishing an automated synthesis and screening workflow

Our goal was to establish a modular approach that ties together digital chemistry and whole animal phenotyping, taking advantage of our previous experience developing kinase inhibitors (KIs)^8,9^. We previously demonstrated that Type II KIs can be usefully divided into three domains for synthesis: a *hinge-binder* that interferes with the kinase’s enzymatic core, a *cap* domain that provides additional target specificity and is an attractive site to focus QSAR, and a *linker* that connects the two^14^.

**Fig. 1:**
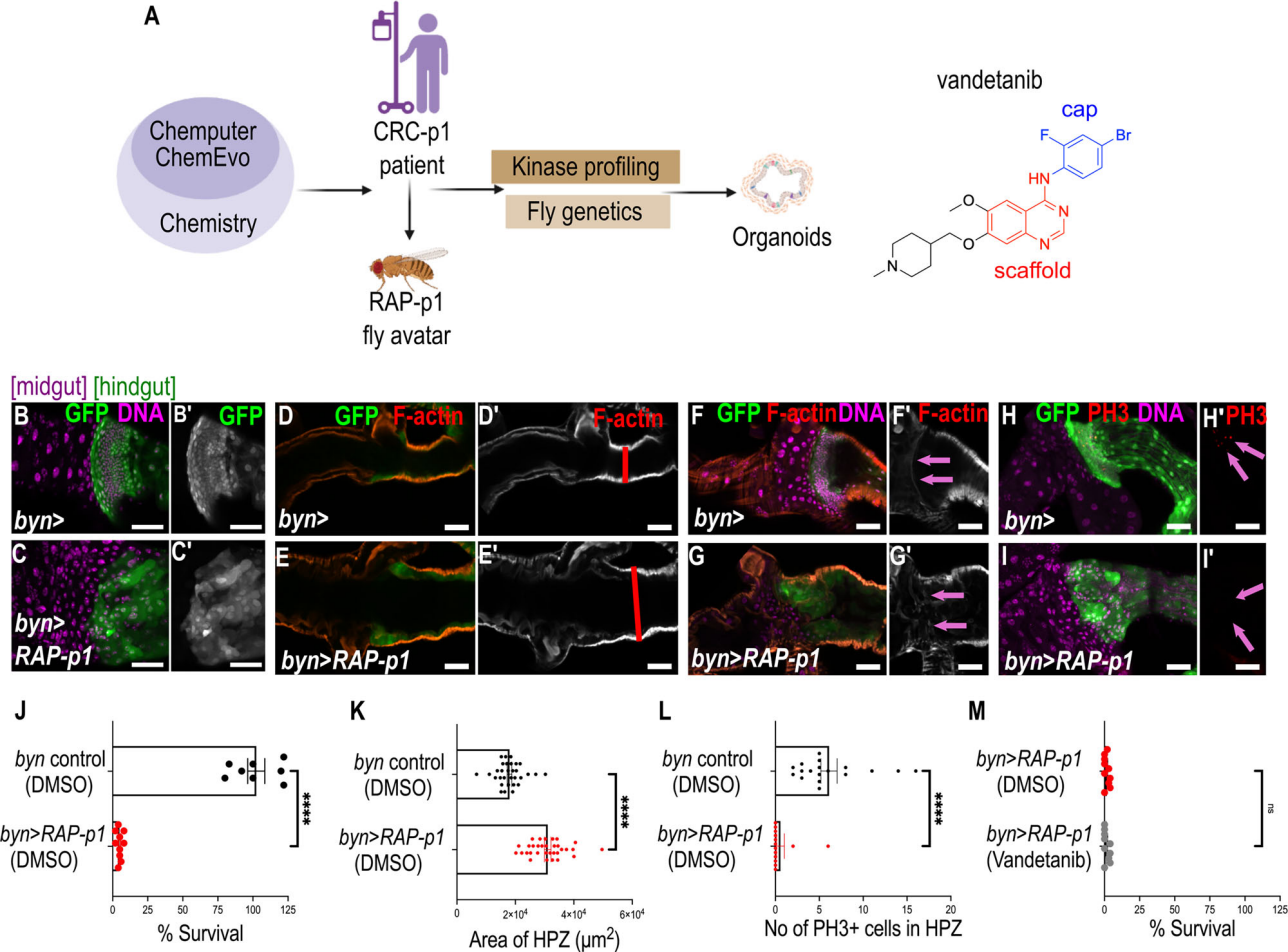
Overview of our approach. **A** Overview of the platform combining chemistry approaches (Chemputer-chemical evolution) with cancer genomics and fly genetics for the discovery of novel KIs. Chemical structure of vandetanib showing the quinazoline scaffold core (red) and cap (blue). **B**, **C** Confocal micrographs of larva hindgut marked with GFP (green) under the control of *byn-Gal4*. The midgut and hindgut regions of the digestive tract are shown in (**B**). **B** *byn-Gal4* control (*byn*>) showing normal morphology of the hindgut proliferation zone (HPZ). **C** Expression of the *RAP-p1* transgene in the hindgut (*byn* > *RAP-p1*) led to abnormal HPZ morphology. Confocal micrographs of GFP (green) labelled hindgut; HPZ marked with F-actin (red, indicated with grayscale in (**D’**) and (**E’**)). Compared with control hindgut which shows normal size (**D**), expression of *RAP-p1* induced thickening of the HPZ (**E**). **F**, **G** Localisation of F-actin (in red, indicated with grayscale and arrows in (**G’**) and (**H’**)) at midgut/hindgut boundary was normal in control gut (**G**) and abnormal in *byn* > *RAP-p1* guts (**H**). **H**, **I** Confocal representation of GFP (green) labelled hindguts; HPZ stem cells marked with PH3 (red, indicated with arrows in (**I’**) and (**J’**)). HPZ stem cells exhibited the ability to regenerate in control (**I**) but not in *byn* > *RAP-p1* HPZ cells (**J**). **J** Plots showing that expressing *RAP-p1* in the hindgut led to organismal lethality. **K** Quantitation of area of HPZ in *byn* > *RAP-p1* hindguts compared to control. Plots showing the number of regenerative stem cells in HPZ in control compared to *byn* > *RAP-p1* (**L**). **M** Plots showing that *byn* > *RAP-p1* flies failed response to vandetanib. Nuclei are labelled with TO-PRO-3 (magenta) in all images. Scale bar in confocal images is 50 µm. ****P < 0.0001 by Student’s *t* test. Error bars represent mean ± SEM. **A** was created with the aid of BioRender.com.

Guided by our previous work in *Drosophila* tumour models^15,16^, we adopted the 4-anilinoquinazoline hinge-binder framework as the starting point for synthesis; its widespread use as a canonical scaffold for KIs reflects its capacity to form key hydrogen bonds within ATP/substrate binding pockets^10,11^. The FDA-approved KI vandetanib—constructed on this scaffold and validated in a fly model of medullary thyroid carcinoma^15,16^—supplied the ‘hinge binder-plus-linker’ scaffold for building our library (Fig. 1A).

**Fig. 2:**
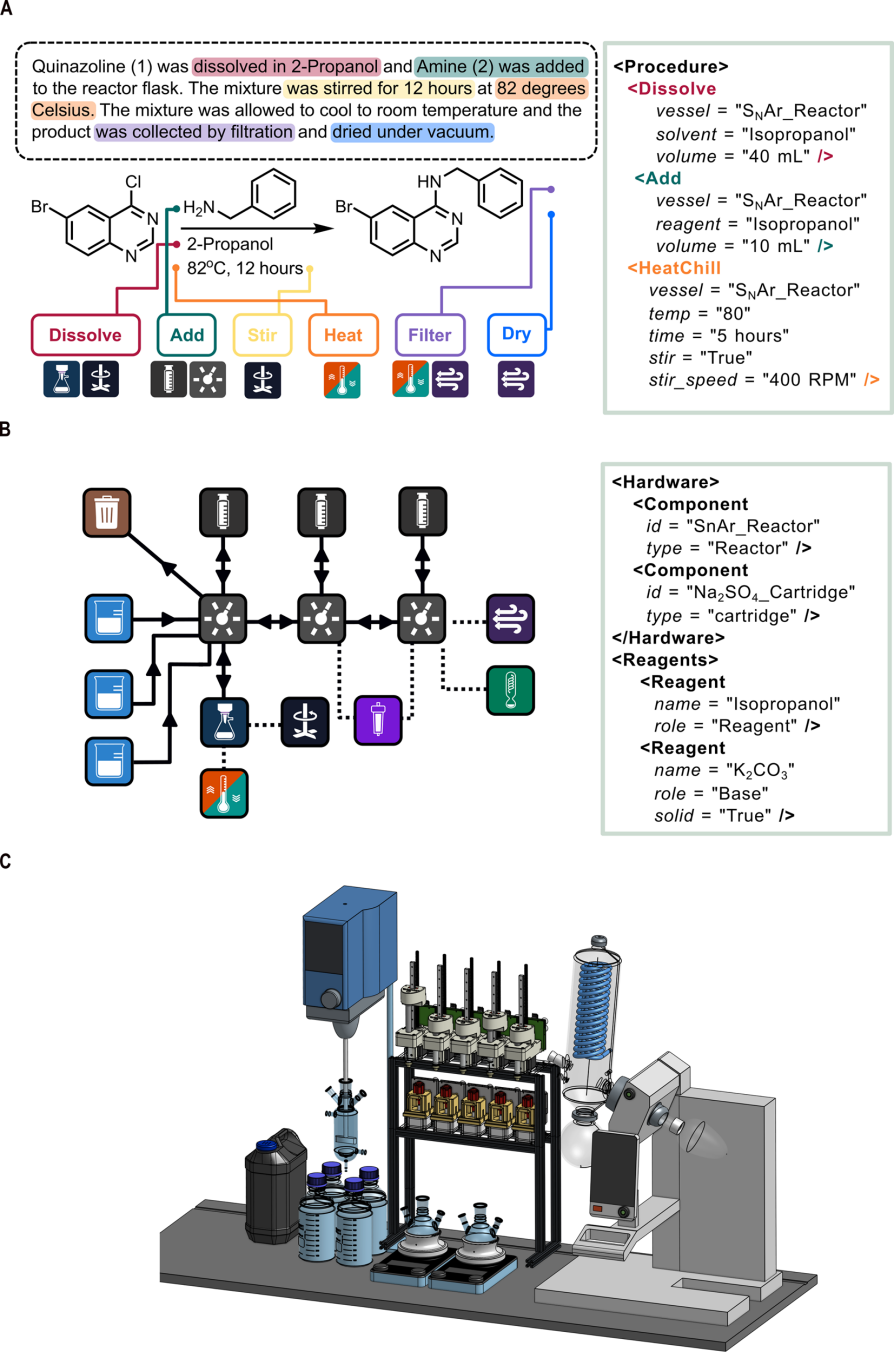
Automated chemputation system. **A** Chemical program representation of the stepwise synthesis of a target compound using automated procedures, highlighting operations such as dissolution, addition, stirring, heating, filtration, and drying. **B** Hardware map of a Chemputer graph describing the setup of the interconnected components required for the synthesis, including pumps, valves, chillers, and reactors. **C** Chemputation platform of the integrated hardware setup used to execute the chemical program autonomously. Hardware and software schematics in (**A**–**C**) were generated using ChemIDE.

To automate our chemical exploration, we used the Chemputer platform, which provides a universal platform for digitizing chemical synthesis at the level of discrete unit operations (e.g., heating, stirring, extraction). Steps are encoded as executable _*X*_DL scripts (Fig. 2A–C)^5^. These scripts are mapped onto the physical configuration of the system using a graph file that captures module connectivity and flow paths, ensuring precise alignment between digital protocols and hardware (Fig. 2B)^17,18^. We leveraged these capabilities to automate the exploration of selected chemical space, linking phenotypic data directly to synthesis inputs (Fig. 2A).

**Fig. 3:**
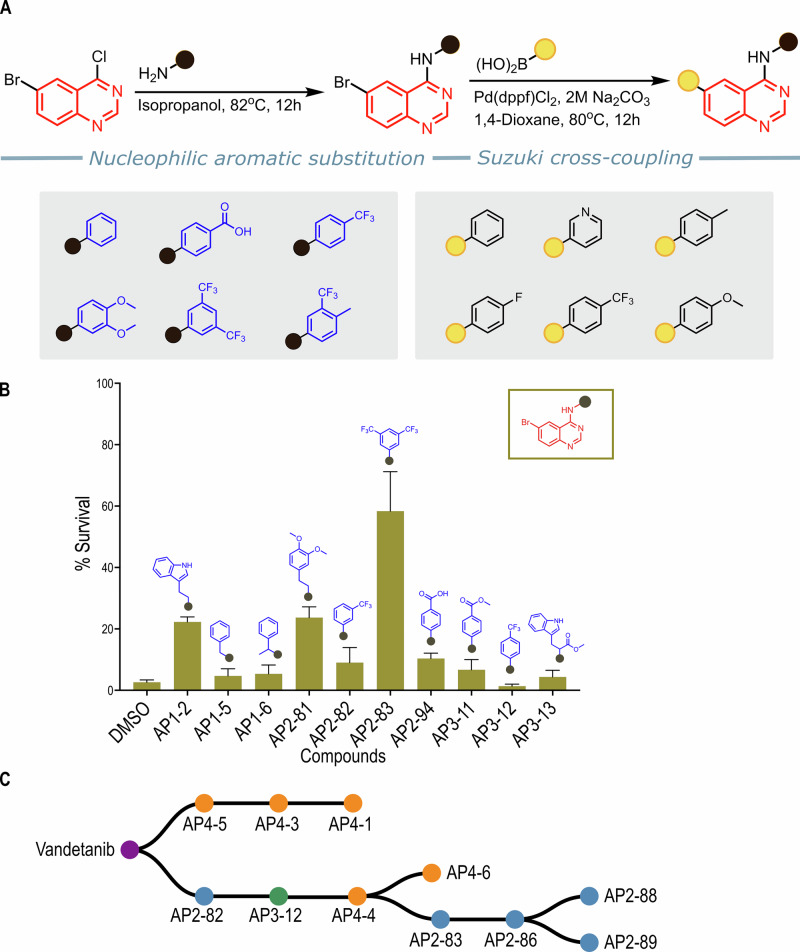
Discovery of AP2-83. Overview of the modular chemical space used in this study; scaffold above, caps below. Molecules were synthesized using nucleophilic aromatic substitution (dark substituents) followed by Suzuki cross-coupling (yellow substituents). **A** The building blocks for each step are shown below their corresponding reaction. **B** Graph showing quantitative structure-activity relationship (QSAR) analysis in *byn* > *RAP-p1* model. Caps are indicated on the x-axis. The y-axis shows the percentage survival of *byn* > *RAP-p1* animals for individual compounds. All compounds were tested by feeding at 10 µM final food concentration. **C** Molecular similarity tree rooted at vandetanib, constructed using an algorithm prioritizing substructure inclusion and iterative molecular weight increase. Each node represents a compound synthesized in the series, with branches reflecting the most probable paths based on structural similarity and fragment addition. Node colours correspond to the dominant building block introduced at each stage. Error bars represent mean ± SEM.

A two-step diversification strategy was designed for automation (Fig. 3A). *Nucleophilic Aromatic Substitution (SnAr)* was used to react the 4-chloro position of the quinazoline core with various anilines to generate a diverse set of ‘cap’ groups. In parallel, the 6-bromo site was modified via Suzuki coupling, allowing fine-tuning of the core despite the somewhat more complex setup. We also introduced linker variants to modulate torsional flexibility, assessing its impact on multi-kinase binding and in vivo activity (Fig. 3A).

Initial quantitative structure–activity relationship (QSAR) focused on commercially available aniline derivatives to balance cost and synthetic ease while capturing informative QSAR. We used a batch-wise approach, introducing sets of substituents while preserving the 4-anilinoquinazoline core (Fig. 3A, B). All analogues were synthesized using standardized _*X*_DL protocols, ensuring repeatability and minimizing procedural drift.

### A Drosophila model for in vivo phenotypic screening

We developed a complex, patient-based Drosophila line to assess compound activity in a whole-animal context. A transgenic line was generated to model a specific stage-IIIB CRC patient tumour (*TCGA-AA-3681-01*) identified in the TCGA cancer database^19^. This ‘personalised fly avatar’ expresses a *UAS-RAP-p1* transgene containing oncogenic *Ras*^*G12V*^ plus shRNAs targeting orthologues of human tumour suppressors *Apc, P53, ago, wts, CG7742*, and *Atg2*; we refer to this 7-hit line as ‘RAP-p1’.

Expression of the RAP-p1 transgene in the larval hindgut (*byn* > *RAP-p1*) induced morphological abnormalities, including expansion of the hindgut proliferation zone (HPZ) and disrupted midgut/hindgut boundaries, consistent with invasive tumour behaviour (Fig. 1B–G, K). While control HPZs displayed PH3-positive stem cells (Fig. 1H, L), *byn* > *RAP-p1* hindguts lacked PH3 staining, suggesting the presence of abnormal, non-regenerative cells despite increased size (Fig. 1I, L). The result was animal lethality, with ~5% of animals surviving to pupariation (Fig. 1J). This lethal phenotype was not rescued by clinically relevant drugs we tested, including vandetanib (Fig. 1M), mirroring CRC patient response. Rescue-from-lethality, therefore, served as a useful quantitative endpoint for a chemical screen.

### Identifying first-generation hit AP2-83

The Chemputer was programmed to synthesize an initial library of 4-anilinoquinazoline analogues, which were evaluated for their ability to rescue the lethal phenotype of *byn* > *RAP-p1* flies. To assess efficacy and toxicity, we tested each Chemputer-synthesised analogue in our *byn* > *RAP-p1* model, which exhibited ~5% survival to pupariation at 27 °C. Compounds were evaluated by mixing into the fly media at 1, 10, and 100 µM final concentration, assessing quantitative structure–activity relationships (QSAR) in a whole-animal context. For example, compounds lacking electron-withdrawing caps (e.g., AP1-5, AP1-6) showed little or no rescue activity (≤3% survival; Fig. 3B).

Although initial benzylamine analogues were weak, compounds with the indole-based AP1-2 achieved 23% survival at 10 µM. However, synthetic limitations prompted us to switch to more tractable substituents. Inspired by tryptamine-like activity, we introduced a dimethoxyphenylethylamine moiety that yielded two leads: AP2-81 (flexible propyl linker, 25% survival) and AP2-83 (rigid di-meta-substituted aniline, 58%; Fig. 3A, B). The improved rescue suggests a role for conformational constraints in activity, although further structural studies are needed to explore this point. Modifications at the 6-position of AP2-83 consistently reduced efficacy (e.g., AP2-86, AP2-88, AP2-89), indicating structural sensitivity at that site. Trifluoro and bulkier tryptophan-like caps (e.g., AP3-11 to AP3-13) also reduced rescue, while bis-dimethoxy substitutions led to solubility issues and poor activity (Fig. 3B).

Our iterative design combined with QSAR allowed us to develop a modular similarity tree of vandetanib and derivatives (Fig. 3C, Supplementary Fig. 1). These results underscore the efficiency of directly pairing phenotypic screening with systematic compound design. Further, they highlighted the AP2-83 cap region as a valuable starting point for further chemical refinement: we further diversified this cap, aiming to balance hydrophobicity and solubility.

**Fig. 4:**
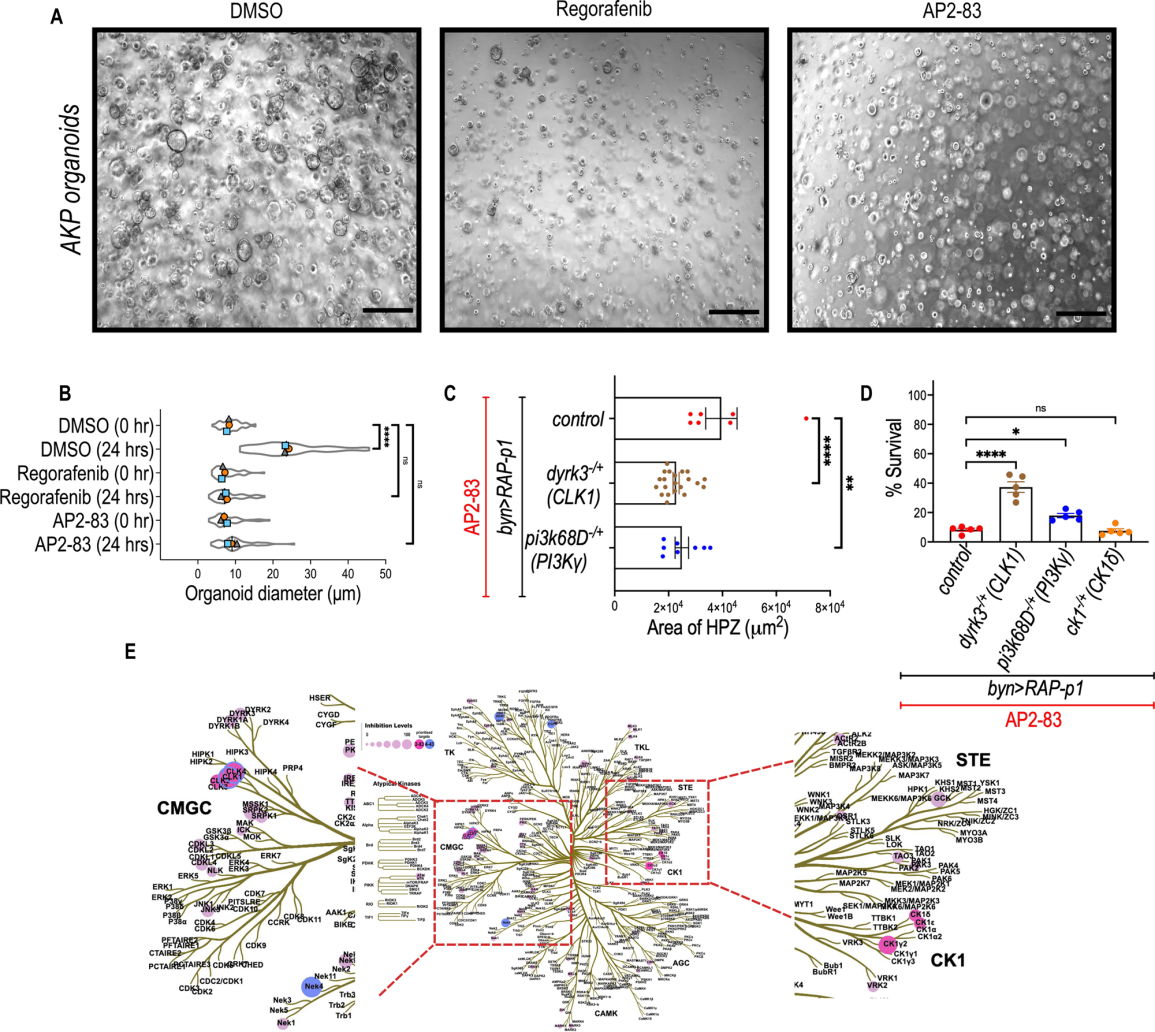
Validation of AP2-83. **A**, **B** Brightfield images of AKP organoids treated with DMSO, 5 µM Regorafenib or 5 µM AP2-83 for 24 h (**A**). Plots showing mean organoid diameter from means of each replicate superimposed on violin plots of organoid diameter measurements for each experimental condition (**B**). **C** Quantitation of the HPZ area in *byn*** >** *RAP-p1* control animals and *byn*** >** *RAP-p1* animals carrying the loss of one genomic copy of kinases animals in the presence of low dose (10 µM) AP2-83. **D** Plots showing that expressing *RAP-p1* in the hindgut led to organismal lethality, which was modified by removing one genomic copy of individual kinases in the presence of low-dose AP2-83. **E** Kinase tree showing AP2-83 kinase profile in magenta. Scale bar in brightfield images is 100 µm. ****P** <** 0.0001, **P** <** 0.005, **P** <** 0.05, by one-way ANOVA with Tukey correction. Error bars represent mean ± SEM.

To assess the relevance of this finding in a mammalian CRC model, AP2-83 was tested in the *Villin Cre*^*ERT2*^*:Apc*^*fl/fl*^ *Kras*^*G12D/+*^ *Trp53*^*fl/fl*^ (AKP) mouse K-CRC organoid line^20^. At 5 µM, AP2-83 inhibited the expansion of AKP organoids to an extent comparable to 5 µM regorafenib, a clinically approved targeted therapy for CRC (Fig. 4A, B). These results further suggest that combining chemputer technology with Drosophila whole-animal models can yield interesting KI hits.

### Target deconvolution of AP2-83

To understand the compound’s mechanism of action, AP2-83 was profiled at 10 µM against a panel of 378 human kinases (Eurofins Discovery Kinase Profiler LeadHunter). Profiling AP2-83 at 10 µM found it to be a less promiscuous compound with a shifted kinase profile compared to its parent compound vandetanib (Supplementary Fig. 2), consistent with our goal to ‘re-tune’ vandetanib for K-CRC. Based on its inhibition profile, AP2-83 most strongly inhibits CLK1, CLK4, CK1∂ and PIK3CG (Fig. 4E, Supplementary Data 1). CLK1 and CLK4 are CDC2-like kinases that affect gene expression through regulation of pre-mRNA splicing; CLK1 is significantly overexpressed in colorectal cancer^21^. PIK3CG is part of a larger complex that can act either in CRC as an oncogene or, more commonly, as a tumour suppressor^22–25^.

The functional relevance of these targets was assessed in vivo using a ‘dominant genetic modifier’ screen in *Drosophila*. Low dose AP2-83 (10 µM final food concentration) was administered to *byn* > *RAP-p1* flies that were also heterozygous for a loss-of-function allele of each candidate AP2-83 target gene. Reducing the gene dosage of the fly orthologues of CLK1 ((*byn* > *RAP-p1; dyrk3*^*–/+*^) and PI3K (*byn* > *RAP-p1;pi3k68D*^*+/*^^−^) significantly decreased HPZ area and increased animal survival compared to flies treated with low-dose AP2-83 alone (Fig. 4C, D). Conversely, reducing CK1∂ dosage did not enhance survival, suggesting it is not a (rate-limiting target) of AP2-83 (Fig. 4D). This genetic interaction suggests that CLK1 and PI3K are functionally important targets of AP2-83 in this biological context, with the caveat that Drosophila does not have a separate orthologue that distinguishes PIK3CA from PIK3CG.

These data indicate that AP2-83 acts at least in part by targeting CLK1 and PI3K, defining a therapeutic network and indicating that targeting these networks could further improve AP2-83 in vivo efficacy. Next, we used this data to improve the overall activity of AP2-83 using a chemical evolution approach.

### Optimising AP2-83 to AP4-43

**Fig. 5:**
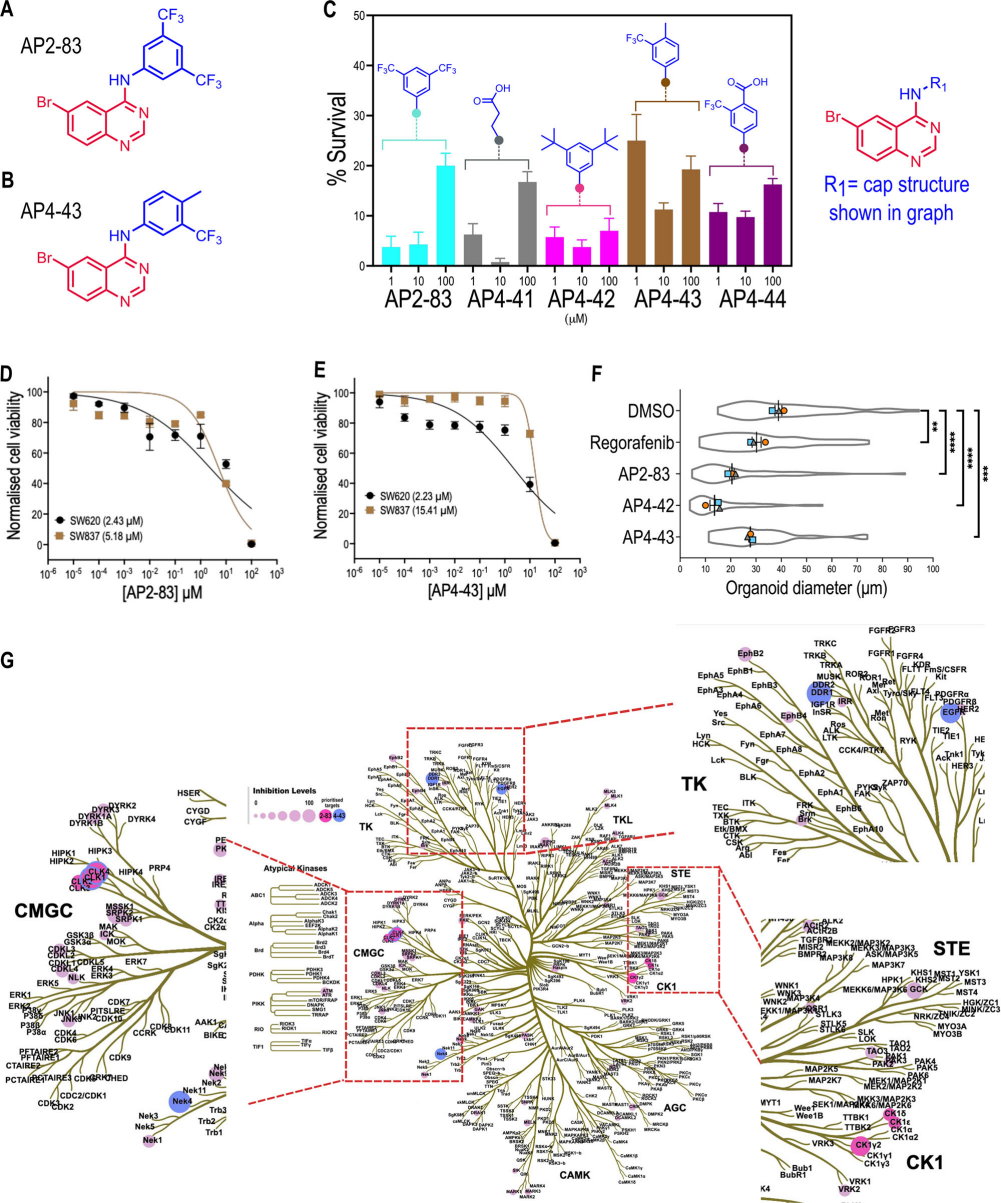
Chemical optimisation of AP2-83. **A**, **B** Chemical structures of AP2-83, AP4-43. **C** Bar chart showing quantitative structure-activity relationship (QSAR) analysis in *byn*** >** *RAP-p1* model. Progressively optimised compounds are indicated on x-axis. y-axis shows the percentage survival of *byn*** >** *RAP-p1* animals for individual compounds. **D**, **E** IC_50_ dose response survival curves for AP2-83 and its derivative AP4-43 on SW620 and SW837 human KRAS-driven colorectal cancer cell lines. Cells were treated for 72 h (*n*** =** 3 biological replicates (mean, SEM)). **F** Plots showing mean organoid diameter from means of each replicate superimposed on violin plots of organoid diameter measurements for each experimental condition treated with DMSO and 2 µM of compounds. (**E**, **G** Kinase tree showing AP4-43 kinase profile in blue. Overlapping activity of AP2-83 and AP4-43 is indicated by blue outlined magenta circles. ****P** <** 0.0001, ***P** <** 0.001, **P** <** 0.005, by one-way ANOVA with Tukey correction. Error bars represent mean ± SEM.

The dominant genetic modifier data from AP2-83 indicated that further refinement was possible. The Chemputer was used to synthesize a small set of analogues with modest changes to the cap region of AP2-83, intended to subtly alter its target profile (Fig. 5A–C)^14^. Changes such as substituting the strongly electron-withdrawing trifluoro groups with bulkier, more lipophilic tert-butyl moieties (AP4-42) did not improve *RAP-p1* survival (Fig. 5C). In contrast, introducing a para methyl group to fine-tune the steric and electronic profile of the compound led to AP4-43, which significantly improved *byn* > *RAP-p1* survival over its parent compound, AP2-83, across multiple concentrations (Fig. 5C). Testing impact on tumour cell survival in 2D cultured human K-CRC cell lines SW620 and SW837 treated for 72 h, AP2-83 showed IC_50_s of 2.43 µM and 5.18 µM, respectively (Fig. 5D); AP4-43 showed similar activity (2.23 µM and 15.41 µM, respectively; Fig. 5E).

In *AKP* mouse CRC organoids, AP4-43 (2 µM) was more effective at reducing organoid growth than the standard-of-care drug regorafenib at the same concentration (Fig. 5F), though less effective than AP2-83 or AP4-42. These data suggest that the platform can enhance the activity and potency of the parent compound in a whole animal setting with relatively few chemical steps, providing a simple feed-forward approach that closely links phenotype with compound efficacy.

### AP4-43 Mechanism of Action in *byn* > *RAP-p1*

In vitro kinase profiling of AP4-43 (10 µM) identified an overlapping but altered target profile compared to AP2-83 (Supplementary Data 1). AP4-43 retained key targets CLK1 and CLK4 but also potently inhibited cancer regulatory kinases DDR1 (collagen-driven adhesion and signalling), EGFR (proliferation and survival), and NEK4 (cell cycle control; Fig. 5G)^26,27^.

**Fig. 6:**
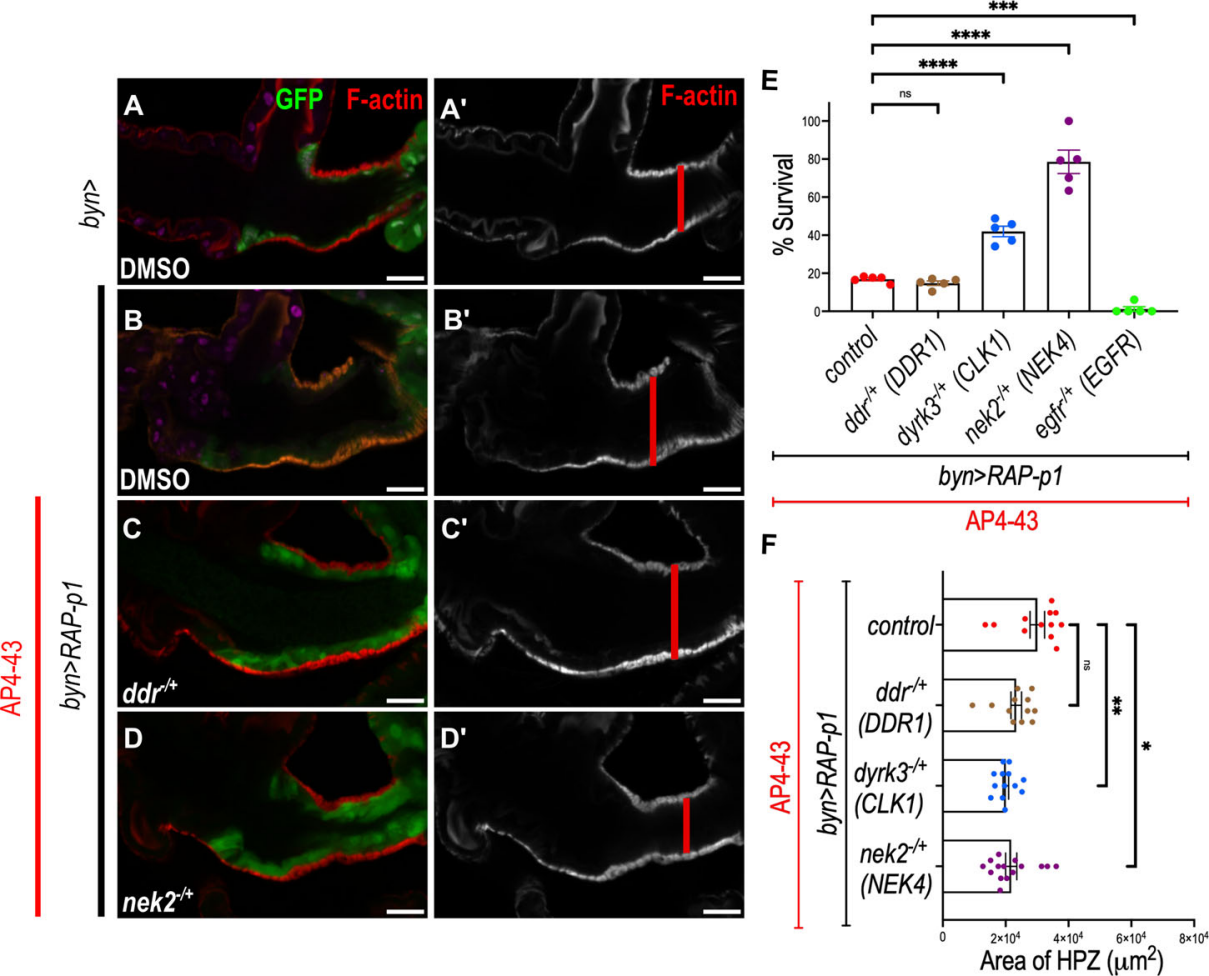
Chemical-genetics screening of AP4-43 targets. **A**–**D** Confocal micrographs of GFP (green) labelled hindgut; HPZ marked with F-actin (red, indicated with grayscale in **A’**, **B’**, **C’**, and **D’**). *byn*** >** *RAP-p1* tumours showed a significant response to the loss of one genomic copy of *nek2* (**D**) but no significant response to the loss of one genomic copy of *ddr* (**C**). **E** Quantification of the HPZ area in *byn*** >** *RAP-p1* control animals and *byn*** >** *RAP-p1* animals heterozygous for kinase gene deletions, treated with low-dose (10 µM) AP4-43. **F** Plots showing that *RAP-p1* expression in the hindgut induces organismal lethality, which was modulated by heterozygous loss of individual kinase genes in the presence of low-dose AP4-43. Scale bar in confocal images is 50 µm. ****P** <** 0.0001, **P** <** 0.005, *P** <** 0.05, by one-way ANOVA with Tukey correction. Error bars represent mean ± SEM.

The functional relevance of these new targets was assessed using our in vivo dominant genetic modifier assay with low-dose AP4-43. Heterozygous loss of the *Drosophila* orthologue of *NEK4* (*byn* > *RAP-p1; nek4*^*-/+*^) in flies treated with AP4-43 produced a significant synergistic effect, increasing survival to 78.5 ± 6.2% (from 16.83 ± 0.76% in AP4-43-only controls, *P* < 0.0001; Fig. 6A–F). This enhanced survival was accompanied by a significant reduction in HPZ area (Fig. 6D, F). Genetic reduction of the CLK1 orthologue (*byn* > *RAP-p1; dyrk3*^*–/+*^) also improved survival, confirming it as a functionally important target. In contrast, reducing *ddr* gene dosage *(byn* > *RAP-p1; ddr⁻/⁺*) had no impact on AP4-43 efficacy (Fig. 6A–F). Heterozygous loss of *egfr* (*byn* > *RAP-p1; egfr⁻/⁺*) reduced survival, suggesting AP4-43 already maximally targets EGFR activity and that further reduction reduces efficacy.

These data indicate that the increased efficacy of AP4-43 is mediated by its engagement with a multi-kinase network that includes the CLK kinases, PI3K, NEK4, and, possibly, EGFR, defining a useful in vivo therapeutic network that matches the complexity of the model.

### Assessing impact on pathway activity in *AKP* organoids

To determine whether AP2-83 and AP4-43 show similar activity in mammalian cells, we used mouse AKP-based 3D organoid cultures to monitor CLK1- and CLK4-dependent signalling together with canonical PI3K and RAS/MAPK pathways. CLK1 activity was assessed by measuring levels of stable active β-catenin (non-phosphorylated on Ser33/Ser37/Thr41 residues), which is regulated via SR protein–mediated splicing, whereas CLK4 (with CLK2) has a defined role in NF-κB activation (phospho-NF-κB).

**Fig. 7:**
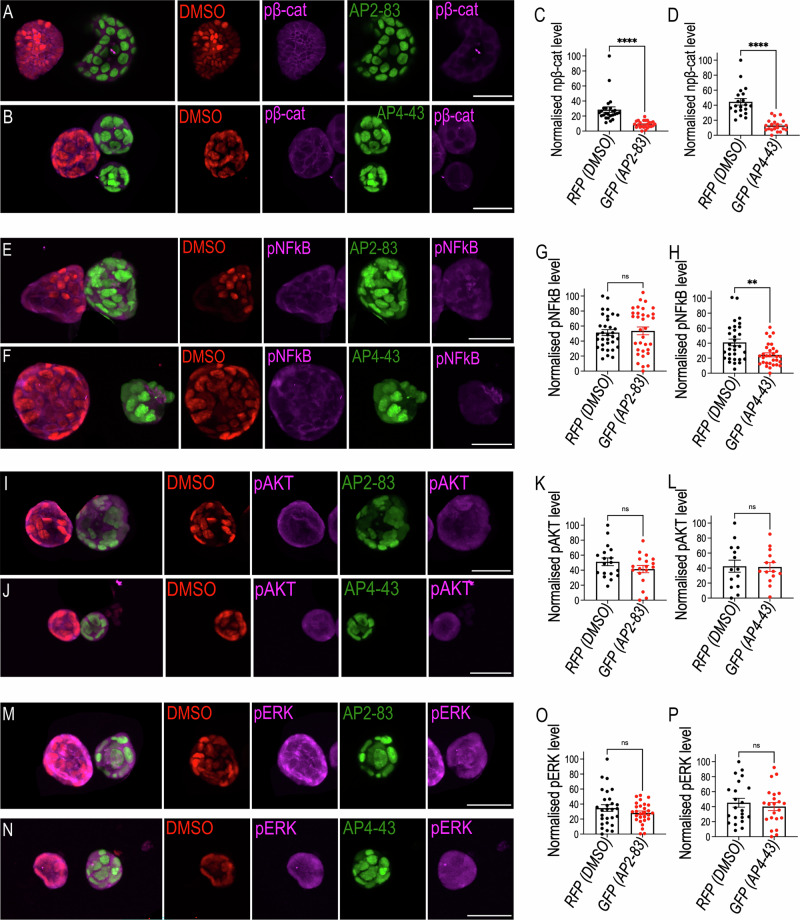
Activity in AKP organoids. Confocal micrographs of mixed organoids containing RFP-labelled DMSO-treated controls (red) and GFP-labelled organoids treated with 2 μM AP2-83 (**A**, green) or 2 μM AP4-43 (**B**, green). Stable non-phosphorylated-β-catenin (npβ-cat) is shown in magenta (single-channel images to the right). **C**, **D** Quantification of normalised npβ-catenin levels in RFP (DMSO) versus GFP (AP2-83 or AP4-43) organoids from (**A**, **B**), indicating a significant reduction in npβ-catenin in both AP2-83- and AP4-43-treated organoids. **E**, **F** Confocal micrographs of mixed DMSO (RFP, red) and AP2-83 (2 µM)- or AP4-43 (2 µM)-treated (GFP, green) organoids stained for phospho-NF-κB (pNF-κB, magenta). Quantification of normalised pNF-κB levels for AP2-83 (**G**) and AP4-43 (**H**), showing no significant change with AP2-83 but a significant reduction with AP4-43 treatment compared with DMSO controls. Confocal micrographs of mixed organoids stained for phospho-AKT (pAKT, magenta) with RFP-labelled DMSO controls (red) and GFP-labelled organoids treated with 2 μM AP2-83 (**I**) or 2 μM AP4-43 (**J**). Quantification of normalised pAKT levels in AP2-83- (**K**) and AP4-43- (**L**) treated organoids, showing no significant differences relative to DMSO controls. Confocal micrographs of mixed organoids stained for phosphorylated-ERK (pERK, magenta) with RFP-labelled DMSO controls (red) and GFP-labelled organoids treated with 2 μM AP2-83 (**M**) or 2 μM AP4-43 (**N**). Quantification of normalised pERK levels for AP2-83 (**O**) and AP4-43 (**P**), showing no significant differences relative to DMSO controls. Scale bars represent 50 µm for each row. Error bars represent mean ± SEM.

Treatment with either AP2-83 or AP4-43 led to a strong reduction in non-phospho-β-catenin compared with DMSO (Fig. 7A–D), consistent with inhibition of CLK1-dependent Wnt/β-catenin signalling and in line with prior studies of the pan-CLK inhibitor cirtuvivint in gastrointestinal cancer models^28,29^. In contrast, only AP4-43 reduced phospho-NF-κB (Fig. 7E–H), mirroring previous work showing that CLK4 relays DNA damage signals to promote NF-κB activation in a manner that is sensitive to small-molecule inhibition^30^. Our kinome profiling is consistent with this view, indicating selective targeting of CLK4 by AP4-43 but not AP2-83.

Neither compound altered the PI3K–AKT pathway readout phospho-AKT (Fig. 7I–L). Failure to alter phospho-AKT is consistent with previous work showing that PIK3CA is the primary regulator of PI3K pathway activity in CRC tumours^31,32^. Another, non-exclusive possibility is that PIK3CA and PIK3CG are at least partially redundant, consistent with the modest, context-dependent benefit from PI3Kα inhibitors in CRC clinical trials^33^.

Finally, neither AP2-83 nor AP4-43 altered phospho-ERK (Fig. 7M–P), consistent with their kinase profiles that indicate that both are active on K-CRC tumours using mechanisms independent of RAS/MAPK. Together, these data support a model in which AP2-83 and AP4-43 act primarily through CLK-dependent suppression of Wnt/β-catenin, with additional AP4-43 impact on NF-κB.

## Discussion

**Fig. 8:**
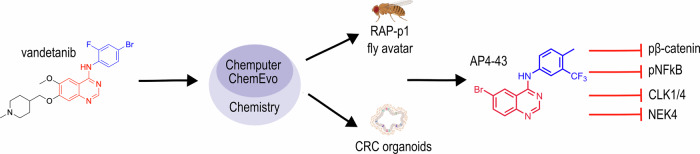
Model for modular platform. An iterative chemical optimisation strategy, guided by a genetically complex patient-matched fly ‘avatar’ and validated in CRC organoids. AP4-43 emerged from this stepwise process with enhanced whole body rescue activity and an altered target profile that includes both direct (CLK1, CLK4, NEK4) and indirect (pNF-κB, and npβ-catenin) functional targets. This model was created with the aid of BioRender.com.

Here, we develop a modular platform that integrates automated synthesis with phenotypic screening to accelerate the discovery of multi-kinase inhibitors optimised for genetically complex cancers. Focusing on a 4-anilinoquinazoline scaffold, we used a multi-step process to identify AP4-43 as a lead compound with strong preclinical efficacy against RAS-mutant colorectal cancer in both *Drosophila* and mammalian models (Fig. 8).

At the core of this approach is tailoring kinase chemistry to the Chemputer, a programmable system that digitizes synthetic protocols into reproducible workflows^34^. By leveraging well-established, automatable reactions—such as SnAr and Suzuki couplings—we ensured high synthetic fidelity and minimized procedural drift. This allowed for quick and scalable SAR cycles. Although our approach is broadly useful, reactions requiring precise quenching, unconventional solvents, or tight thermal control would currently prove challenging for our platform.

Our iterative chemical optimisation strategy, guided by a complex patient-matched fly ‘avatar’ (*byn* > *RAP-p1*) survival assay, enabled us to refine compounds iteratively and in a whole-animal context. AP4-43 emerged from this stepwise process with enhanced activity and a broadened target profile, including CLK1, CLK4, NEK4, EGFR, and DDR1. These kinases represent key nodes in pathways controlling proliferation, splicing, DNA damage, and stress responses^21,26,27,35^. Functional genetic experiments confirmed the relevance of these targets. That is, AP4-43 both helped us identify a candidate therapeutic and define a therapeutic network tailored to *RAP-p1*. These targets appear to be mostly conserved in mouse *AKP* organoids, with the complexity that a role for PIK3CG is still unclear. Also of note, AP2-83 showed better activity in mouse organoids, either reflecting (i) differences in whole animal vs. cultured cells, such as toxicity, or (ii) differences between mouse and fly.

By combining digitized synthesis with in vivo screening, our platform enables the discovery of biologically calibrated, multi-targeting compounds more efficiently than traditional medicinal chemistry. Importantly, the ability to quickly identify functionally important targets using standard kinase panels makes polypharmacology more practical for subsequent IND-enabling studies. This, in turn, allows for a broader approach to drug development. While applied here to CRC, our platform is readily adaptable to other diseases where polypharmacology can offer a therapeutic advantage. The ability to define complex networks also opens the possibility of creating useful tools for exploring complex biology.

In summary, our Chemputer–chemical optimisation platform represents a scalable and reproducible framework for discovering complex therapeutics and biological probes. AP4-43 serves as a proof-of-concept for its potential in addressing treatment-resistant CRC and, by analysing its kinase profile, it defines a multi-targeted in vivo therapeutic profile. More broadly, this study provides a practical example of how such technology can be applied to address challenges in preclinical drug discovery.

## Methods

### *Drosophila* stocks and genetics

Flies were maintained at 25 °C using standard protocols. Cancer models were generated using the GAL4/UAS system with temperature-sensitive GAL80^ts^ to control transgene expression: GAL4 was suppressed at 18 °C and activated at higher temperatures, in this study 27 °C. *w*^*1118*^ was used as the wild-type control.

For lethality experiments, tumours were generated by crossing parental flies (*byn-GAL4, UAS-GFP, GAL80ts* with *UAS-RAP-p1* or *w*^*1118*^) at 18 °C for 24 h; parents were then removed, and vials were kept at 18 °C for another 24 h. After a total of 48 h at 18 °C, vials were moved to 27 °C until eclosion.

For dissection experiments, hindgut tumours were generated by crossing parent flies at 18 °C for 24 h, parents were removed, and vials were left at 18 °C for another 24 h. After 24 h, vials were moved to 27 °C and 3rd instar larvae were dissected after 5 days at 27 °C.

### Immunofluorescent staining

*Drosophila* larva hindguts were dissected in PBS and fixed with 3.7% PFA in PBS. The hindguts were washed with PBS plus Tween 20 (PBST; 1x PBS, 0.2% Tween 20) three times for 15 minutes each time. Hindguts were then blocked with PBTB (1x PBS, 0.2% Tween 20, 5% FBS) for 1 hour at room temperature. The hindguts were treated with primary antibodies in PBTB at 4  °C overnight. The primary antibodies were washed with PBST three times for 15 minutes and then blocked with PBTB for 1 hour at room temperature. Phalloidin 546 (1:50; Molecular Probes, A22283) was used to stain F-actin or secondary antibody at 4 °C overnight. Hindgut was washed with PBST for 15 min before staining nuclei with TOPRO-3 (1:1000; Life Technologies, T3605) in PBST for 15 min. Hindgut was mounted in VECTASHIELD (Vector Laboratories).

### Image acquisition and analysis of fixed hindgut samples

Images were taken on a confocal microscope (LSM 780; Carl Zeiss) using a 20x/0.5 NA air objective. Three laser lines were used based on the excitation wavelength of the staining dyes, which included 488-nm, 561-nm, and 633-nm wavelengths. ImageJ software (https://imagej.nih.gov/ij/) was used for the quantification of the hindgut proliferation zone area and the diameter of organoids.

### Organoids culture and compound treatment

#### Mouse organoid line

Colorectal cancer organoids line expressing oncogenic Ras plus loss of Apc and p53 (Villin Cre^ERT2^: Apc^fl/fl^ Kras^G12D/+^ Trp53^fl/fl^; obtained from Owen Sansom, CRUK Scotland Institute, UK) were cultured in Advanced DMEM/F12 supplemented with 2 mM Glutamine, 10 mM HEPES, 100/100 U/ml Pen/Strep, 1x N2/B27, 50 ng/ml EGF, and 100 ng/ml Noggin. Basal media for passaging contained Advanced DMEM/F12, Glutamine, HEPES, and Pen/Strep. Organoids were embedded in 100% growth factor-reduced (GFR) Matrigel (Corning).

Organoids were passaged 1:2 every 3-4 days: growth media was removed, and Matrigel was resuspended in cold basal media and transferred to 15 ml Falcon tube. The organoids were mechanically dissociated by trituration with p200 pipette tips for 20-30 times. Single cell organoids were generated with an additional step of TrypLE treatment for 7 min at 37 °C. The cells were centrifuged at 1000 rpm for 3 minutes and the supernatant was aspirated. Cells were then washed with 5 ml of basal media and triturated 20 times. Cells were centrifuged for 3 minutes at 1000 rpm. Pelleted cells were then resuspended in 120 µL 100% Matrigel and plated as 20 µL domes in 6-well plates supplemented with organoid culture media.

#### Compound screening

Organoids were plated as 10 µL domes of Matrigel containing 3000 cells in 24-well plates. The domes were supplemented with organoid culture media containing DMSO or Chemputer-generated compounds.

### Adherent cell-line culturing

*SW620* and *SW837* are KRAS-driven human colorectal cancer cell lines that were obtained from the American Type Culture Collection (ATCC). Cell lines were grown in DMEM-GlutaMAX (10% foetal bovine serum (FBS) and 1% penicillin-streptomycin) at 37 °C, 5% CO_2,_ and split at confluency.

### Compound efficacy assay in cell lines

To assess the ability of each compound to reduce cell number in culture, 3000 cells were seeded per well in uncoated 96-well plates. Cells were treated with Dimethyl sulfoxide (DMSO control), AP2-83, and AP4-43 after 24 h of seeding. The final concentrations of DMSO were kept at 0.1%. Cell viability was measured 72 h post-compound treatment with fluorescence from CellTiter-Blue. Viability data were analysed by normalising the treatment wells with the DMSO control wells. IC_50_ was calculated with a non-linear regression fit in GraphPad Prism 10.

### Development of labelled organoids

The lentiviral vectors, pLV-CMV-mcherry(NLS)-Puro and pLV-CMV-GFP (NLS)-Puro, were synthesised by VectorBuilder to label organoids with red or green fluorescent proteins (RFP or GFP), respectively. HEK293T cells were obtained from American Type Culture Collection (ATCC). To generate lentiviral particles, HEK293T cells were transfected with 0.5 µg of lentiviral vector, 2 µg of psPAX2, and pCMV-VSV-G (packaging vectors) at 37 °C, 5% CO_2_ for 8 h. The transfection supernatant was removed and replaced with organoid culture media. Lentiviral supernatants were collected and cleared using a 0.45 µm syringe filter. The supernatant was then used to infect CRC organoids in the presence of 5 µg/ml polybrene. GFP or RFP organoids were selected using 10 µg/ml of puromycin.

### Immunofluorescence of treated organoids

RFP positive organoids were treated with 0.1% DMSO for 24 h, while GFP positive organoids were treated with 2 µM of AP2-83 or AP4-43 for 24 hours. After 24 hours, intact organoids were collected for immunofluorescence by dissolving Matrigel in cell recovery solution (Corning). RFP and GFP organoids were mixed gently. The mixed organoids were then plated into 8 well chamber µ-slide in PBS (Ibidi). Mixed organoids were fixed with 3.7% PFA in PBS. The organoids were washed with PBS plus Tween 20 (PBST; 1x PBS, 0.2% Tween 20) three times for 15 minutes each time. Mixed organoids were then blocked with PBTB (1x PBS, 0.2% Tween 20, 5% FBS) for 1 hour at room temperature. The organoids were treated with primary antibodies in PBTB at 4 °C overnight. The primary antibodies were washed with PBST three times for 15 min and then blocked with PBTB for 1 hr at room temperature. The organoids were then incubated with Alexa Fluor-conjugated secondary antibody (1:500, Life Technologies, A21070) at 4 °C overnight. Primary antibodies were from Cell Signaling Technology. The primary antibodies used are rabbit anti-phospho-AKT (4060 L, 1:100), rabbit anti-[non-phospho-S33/S37/T41]-beta-catenin (8814S, 1:100), rabbit anti-phospho-ERK (9101S, 1:100), and rabbit anti-phospho-NF-kB (3033S, 1:100).

### Image acquisition and analysis of fixed mixed organoids

Images were taken on a confocal microscope (LSM 780; Carl Zeiss) using a 20x/0.5 NA air objective. Three laser lines were used based on the excitation of wavelength of the fluorescent proteins and staining dyes, which included 488-nm, 561-nm, and 633-nm wavelengths. ImageJ software (https://imagej.nih.gov/ij/) was used for the quantification of the staining intensity in organoids.

### In-vitro kinase profiling

AP2-83 and AP4-43 inhibitory profiles were performed using the Eurofins Discovery Kinase Profiler LeadHunter platform, a radiometric assay that measures kinase activity in the presence of test compounds. Kinases were incubated with peptide substrates in the presence of radiolabelled ATP tracers in the reaction buffer. 10 µM of AP2-83 or AP4-43 was added to the mix; reactions were then initiated by the addition of 10 mM Magnesium Acetate, incubated for 40 minutes at room temperature, and stopped with 0.5% v/v phosphoric acid. 1 µL of each reaction was spotted onto filter paper, washed in 0.425% v/v phosphoric acid four times for 4 minutes, then washed in methanol and left to dry. Phosphorylated products were quantified by scintillation counting. Inhibitory profile trees were visualised using Coral^36^.

### Statistics and reproducibility

Sample size was selected based on previous experience, and no statistical method was used to predetermine the sample size. No data were excluded from the analysis. All results are expressed as the mean ± standard error of mean from *n* > 2 biological replicates. Statistical tests were performed with GraphPad Prism 10, including Student’s *t* tests and, for multiple comparisons, one-way ANOVA with Tukey correction. Statistical significance was set at *P* < 0.05. Raw data is provided in Supplementary Data 2.

### Chemistry

The automated synthesis platform configuration, experimental details of specific compounds, and NMR spectra are described in Supplemental Information.

Solvents and reagents were used as received from commercial suppliers unless otherwise stated. NMR measurements were performed with a Bruker Avance III HD 600 spectrometer operating at 600.1 and 150.9 MHz for ^1^H and ^13^C, respectively. Spectra were collected at 298 K, chemical shifts are reported in ppm and were calibrated for the NMR solvent signal. Multiplicities are given as singlet (s), doublet (d), triplet (t), quartet (q) and multiplet (m), with coupling constants reported in Hz. The spectra were processed using MesReNova 14.2.2. ESI-MS Measurements. Performed using a Bruker MaXis Impact instrument in MeCN/H_2_O. A standard tuning mix was used, and the machine was calibrated for 100–1500 m/z. All scans were recorded with positive ion polarity, and the capillary tip voltage was set to 4000 V. Spectra were processed with Bruker’s DataAnalysis 4.1 software.

#### General procedure for aromatic substitution reactions

To a solution consisting of halide (1 eq.) in isopropanol (5 mL/mmol) was added amine (1.1 eq). The reaction mixture was heated (82 °C) and stirred under a flow of nitrogen. After 15 hours, the reaction mixture was cooled to room temperature and then the solvent was removed over a fritted funnel. The filtered solid was rinsed with excess isopropanol.

#### General procedure for Suzuki Cross-Coupling reactions

A dry 100 mL, three-necked flask was evacuated and refilled with nitrogen and charged with halide (1 eq.) and boronic acid (1.5 eq.). 1,4-Dioxane (8 mL) and 2 M Na_2_CO_3_ (2 mL) were added, and the mixture was purged with nitrogen for 30 minutes. Pd(dppf)Cl_2_ (10 mol%) was added and the mixture was further purged with nitrogen for 15 minutes. The solution was then heated to 85 °C for 12 hours under a nitrogen atmosphere. After the reaction time had elapsed, the contents of the reactor flask were filtered through a celite filter. The filter was subsequently washed with ethyl acetate. Water was added to the mixture and the two layers were separated. The organic layer was washed four times with 1 M sodium hydroxide, followed by three washes with brine. The combined organic layers were dried over magnesium sulfate, and the solvent was evaporated in vacuo. The resulting solid was re- dissolved in a minimal volume of ethyl acetate and passed through a 10 g Biotage KP-Sil silica column.

### Figure preparation

Figures 1a and 8 were created by the authors using BioRender.com under a professional publication license. Figure 2a–c was generated using ChemIDE (Cronin Laboratory). All chemical structures were rendered using ChemDraw Prime (institutional license R-25.0.2).

### Reporting summary

Further information on research design is available in the Nature Portfolio Reporting Summary linked to this article.

## Supplementary information

Transparent Peer Review file

Supplemental Information

Description of Additional Supplementary Files

Supplementary Data 1

Supplementary Data 2

Reporting Summary

## Data Availability

All relevant data supporting the findings of this study are available within the article and its Supplementary Information files, or from the corresponding author upon request. Specifically, kinase profiling data are provided in Supplementary Data 1 and raw screening data are in Supplementary Data 2. R. C. is responsible for replying to data requests.
