# Supplementary material for: Chemical programming of kinase inhibitors in a modular chemputer-based system
Source: Commun Biol. 2026 Mar 27;9:874. doi: 10.1038/s42003-026-09873-8 (PMC13315788; doi:10.1038/s42003-026-09873-8)
Supplement: Supplementary file 2 — Supplemental Information [file 42003_2026_9873_MOESM2_ESM.pdf]

Chemical Programming of Kinase Inhibitors in a Modular Chemputer- Based System- Supplemental Figures

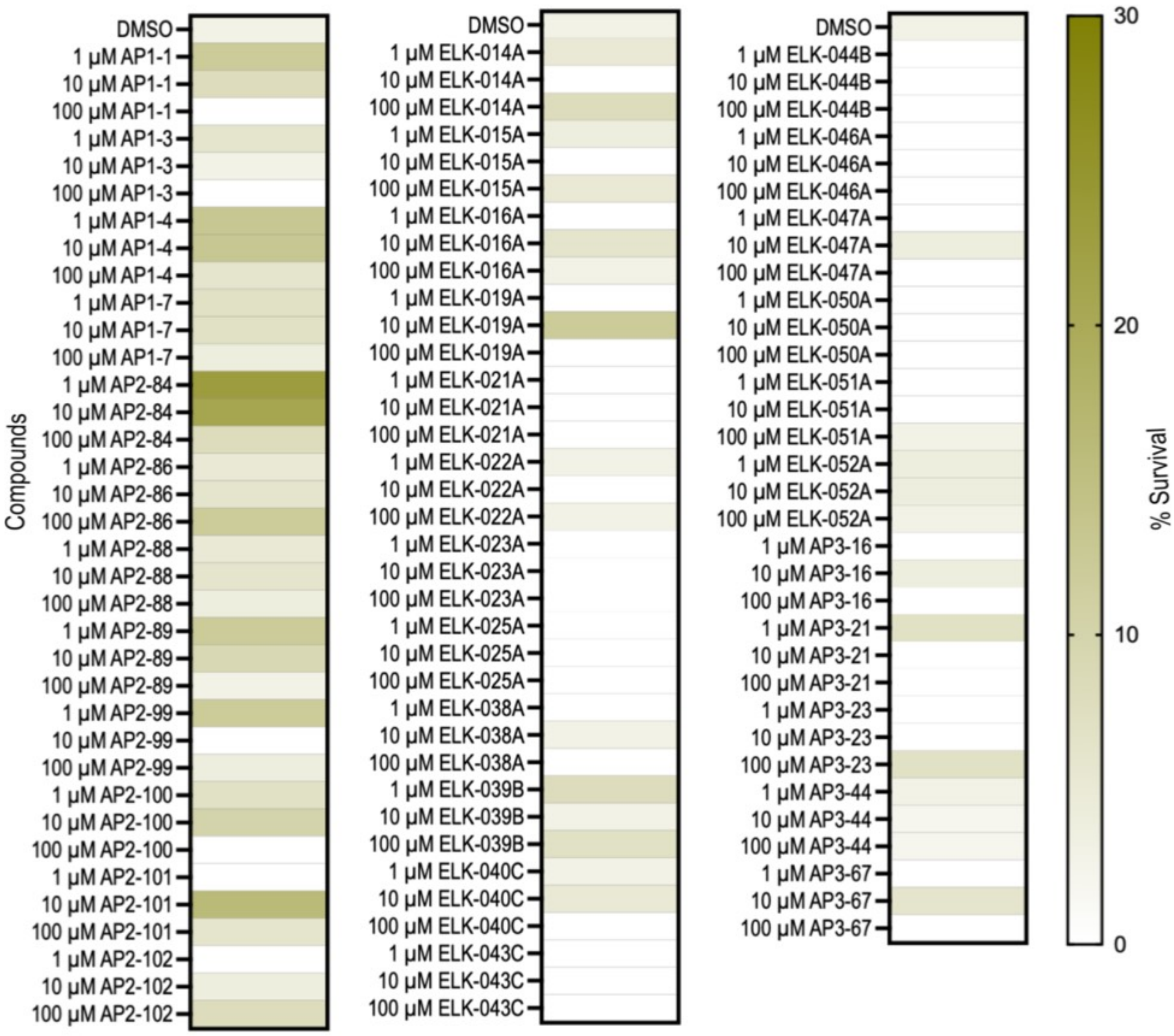

Supplementary Figure 1:

Heatmap showing the mean percentage survival of *byn>RAP-p1* animals for 1, 10, 100 μM of compounds.

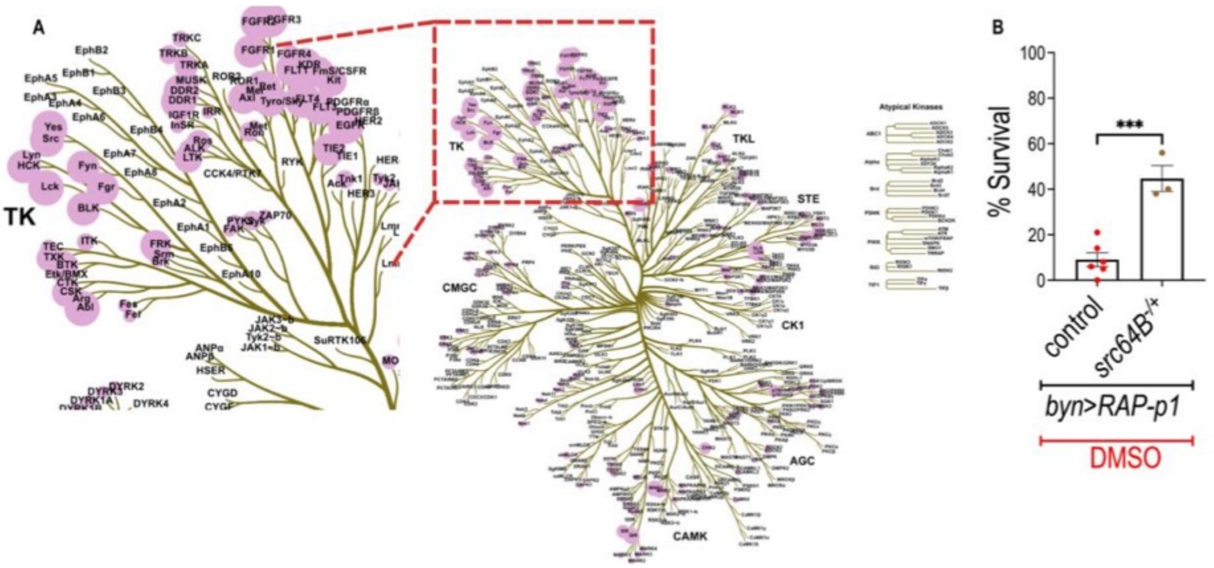

Supplementary Figure 2:

(A) Kinase tree showing vandetanib kinase profile. **(B)** Plot showing that *byn>RAP-p1* lethality was rescued by loss of one genomic copy of *src* (*byn>RAP-p1; src64B*$^{+/-}$).

Chemical Programming of Kinase Inhibitors in a Modular Chemputer- Based System- Supplemental Methods

Contents

Materials and Methods 3

Automated Synthesis Platform Configuration 3

(1) General procedure for nucleophilic aromatic substitution 4

(2) General procedure for Suzuki Cross-Coupling reactions 4

Experimental details 6

N-phenylquinazolin-4-amine (AP2-1): 6

N-benzylquinazolin-4-amine (AP2-2): 6

6-bromo-N-quinazolin-4-amine (AP2-3): 7

N-(2-(1H-indol-3-yl)ethyl)-6-bromoquinazolin-4-amine (AP2-7): 7

N-benzyl-6-phenylquinazolin-4-amine (AP2-8): 8

6-bromo-N-(3,4-dimethoxyphenethyl)quinazolin-4-amine (AP2-81): 8

6-bromo-N-(3-(trifluoromethyl)phenyl)quinazolin-4-amine (AP2-82): 9

N-(3,5-bis(trifluoromethyl)phenyl)-6-bromoquinazolin-4-amine (AP2-83): 9

N-(3,4-dimethoxyphenethyl)-6-phenylquinazolin-4-amine (AP2-84): 10

N-(3,5-bis(trifluoromethyl)phenyl)-6-phenylquinazolin-4-amine (AP2-86): 10

N-(3,5-bis(trifluoromethyl)phenyl)-6-(4-methoxyphenyl)quinazolin-4-amine (AP2-89): .. 11

4-((6-bromoquinazolin-4-yl)amino)benzoic acid (AP2-94): 11

N-benzyl-6-(4-fluorophenyl)quinazolin-4-amine (AP2-99): 12

N-benzyl-6-(p-tolyl)quinazolin-4-amine (AP2-100): 12

N-benzyl-6-(4-methoxyphenyl)quinazolin-4-amine (AP2-101): 13

N-benzyl-6-(4-(trifluoromethyl)phenyl)quinazolin-4-amine (AP2-102): 13

Methyl-4-((6-bromoquinazolin-4-yl)amino)benzoate (AP3-11): 14

6-bromo-N-(4-(trifluoromethyl)phenyl)quinazolin-4-amine (AP3-12): 14

N-[4-methyl-3-(trifluoromethyl)phenyl]-6-bromoquinazolin-4-amine (AP4-2): 15

N-(3-fluoro-5-(trifluoromethyl)phenyl)quinazolin-4-amine (AP4-3): 15

N-(2-fluoro-4-(trifluoromethyl)phenyl)quinazolin-4-amine (AP4-5): 16

N-(2-fluoro-4-(trifluoromethyl)phenyl)-6-bromoquinazolin-4-amine (AP4-6): 16

3-(6-bromoquinazolin-4-ylamino)propanoic acid (AP4-41): 17

N-(3,5-di-tert-butylphenyl)-6-bromoquinazolin-4-amine (AP4-42): 17

N-(4-methyl-3-(trifluoromethyl)phenyl)-6-bromoquinazolin-4-amine (AP4-43): 18

2-[(6-bromoquinazolin-4-yl)amino]-5-fluoro-4-(trifluoromethyl)benzoic acid (AP4-44): 18

6,7-dimethoxy-N-(3,4-difluorobenzyl)quinazolin-4-amine (ELK-014A): 19

6,7-dimethoxy-N-(2-morpholin-4-ylethyl)quinazolin-4-amine (ELK-015A): 19

6,7-dimethoxy-N-(2-(4-methylpiperazin-1-yl)ethyl)quinazolin-4-amine (ELK-016A): 20

6,7-bis(2-methoxyethoxy)-N-(3,4-difluorobenzyl)quinazolin-4-amine (ELK-019A): 20

6,7-bis(2-methoxyethoxy)-N-(2-morpholin-4-ylethyl)quinazolin-4-amine (ELK-021A): 21

6,7-bis(2-methoxyethoxy)-N-(2-(4-methylpiperazin-1-yl)ethyl)quinazolin-4-amine (ELK-022A): 21

N-[2-(3,4-dimethoxyphenyl)ethyl]-6,7-dimethoxyquinazolin-4-amine (ELK-023A): 22

6,7-dimethoxy-N-((thiophen-2-yl)methyl)quinazolin-4-amine (ELK-038A): 22

N-(furan-2-ylmethyl)-6,7-dimethoxyquinazolin-4-amine (ELK-039B): 23

6,7-dimethoxy-N-(2-(thiophen-2-yl)ethyl)quinazolin-4-amine (ELK-040C): 23

6-bromo-N-(thiophen-2-ylmethyl)quinazolin-4-amine (ELK-043C): 24

6-bromo-N-(2-(thiophen-2-yl)ethyl)quinazolin-4-amine (ELK-046C): 24

N-(thiophen-2-ylmethyl)quinazolin-4-amine (ELK-047A): 25

6-iodo-N-(thiophen-2-ylmethyl)quinazolin-4-amine (ELK-050A): 25

6-iodo-N-(furan-2-ylmethyl)quinazolin-4-amine (ELK-051A): 26

6-iodo-N-(2-(thiophen-2-yl)ethyl)quinazolin-4-amine (ELK-052A): 26

NMR Spectra 27

References 68

Materials and Methods

Solvents and reagents were used as received from commercial suppliers unless otherwise stated. NMR measurements were performed with a Bruker Avance III HD 600 spectrometer operating at 600.1 and 150.9 MHz for ^{1}H and ^{13}C respectively. Spectra were collected at 298 K, chemical shifts are reported in ppm and were calibrated for the NMR solvent signal. Multiplicities are given as singlet (s), doublet (d), triplet (t), quartet (q) and multiplet (m), with coupling constants reported in Hz. The spectra were processed using MesReNova 14.2.2. ESI-MS Measurements. Performed using a Bruker MaXis Impact instrument in MeCN/H_2O. A standard tuning mix was used, and the machine calibrated for 100-1500 m/z. All scans were recorded with positive ion polarity, and capillary tip voltage set to 4000 V. Spectra were processed with Bruker's DataAnalysis 4.1 software.

Automated Synthesis Platform Configuration

The automated synthesis platform employed in this study utilized a modular Chemputer system, as previously described[1] consisting of four to six syringe pumps and six multi-position selection valves for reagent handling and flow routing. Temperature control was achieved using IKA RCT Digital hotplate stirrers with PT1000 temperature probes for in-vessel monitoring. Mechanical agitation was provided by either IKA Eurostar 60 overhead stirrers or integrated magnetic stirring via the IKA RCT Digital stirrer plates. All reactions were performed in standard laboratory glassware fitted with appropriate septa and ground glass joints.

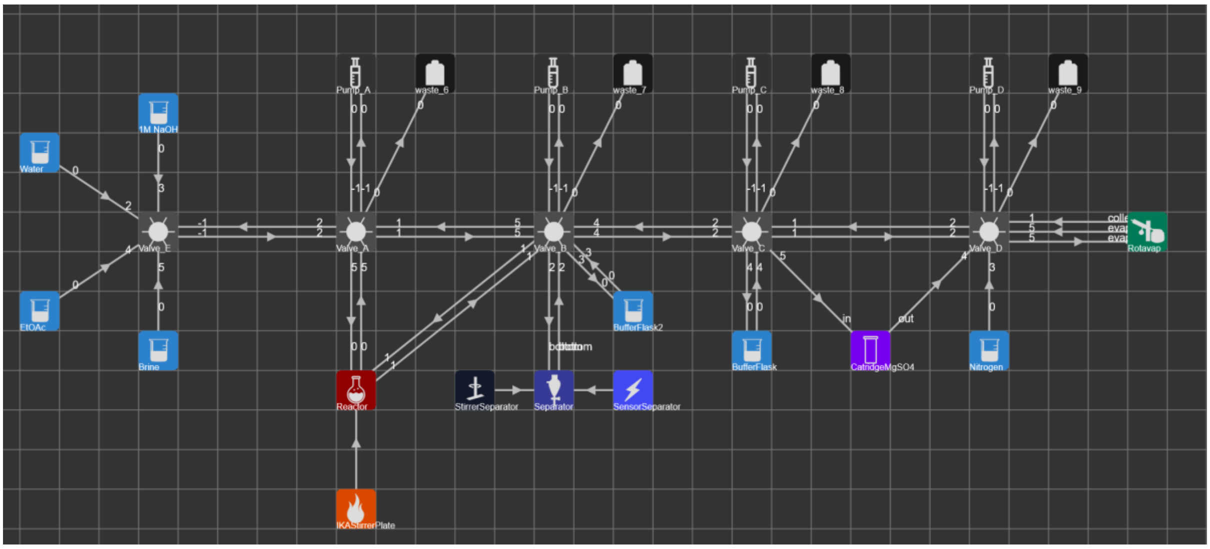

Figure S1: Layout of the Chemputer platform showing the arrangement of pumps, valves, reactor flasks, and supporting hardware used for all synthetic procedures.

(1) General procedure for nucleophilic aromatic substitution: To a solution consisting of halide (1 eq.) in isopropanol (5 mL/mmol) was added amine (1.1 eq). The reaction mixture was heated (82°C) and stirred under a flow of nitrogen. After 15 hours, the reaction mixture was cooled to room temperature and then the solvent was removed over a fritted funnel.

Manual preparations

All reagent flasks were connected to a passive (non-controllable) nitrogen supply. Solid starting materials were manually loaded into the reactor flasks, with all subsequent operations handled by the automated platform according to the XDL protocol. The solid products were manually collected following filtration and dried overnight in a desiccator.

Automated Synthesis

1) Confirm halide (5 mmol) is in reactor.

2) Add Isopropanol (50 mL) directly to reactor at t default speed with stirring at 250 RPM.

3) Add amine (5.5 mmol) directly to reactor at t default speed with stirring at 250 RPM.

4) Heat/Chill reactor to 82°C for 15 h with stirring at 650 RPM. Temperature control is continued after the temperature has been reached.

5) Heat/Chill reactor to 25°C without stirring. Temperature control is continued after the temperature has been reached.

6) Filter contents of filter without stirring, discarding filtrate using standard transfer speeds.

(2) General procedure for Suzuki Cross-Coupling reactions: A dry 100 mL, three-necked flask was evacuated and refilled with nitrogen and charged with halide (1 eq.) and boronic acid (1.5 eq.). 1,4-Dioxane (8 mL) and 2M Na_2CO_3 (2 mL) were added, and the mixture was purged with nitrogen for 30 minutes. Pd(dppf)Cl_2 (10 mol%) was added and the mixture was further purged with nitrogen for 15 minutes. The solution was then heated to 85°C for 12 hours under a nitrogen atmosphere. After the reaction time had elapsed, the contents of the reactor flask were filtered through a celite filter. The filter was subsequently washed with ethyl acetate. Water was added to the filtrate, and the two layers were separated and the aqueous layer was extracted with ethyl acetate. The combined organic layers were washed four times with 1M sodium hydroxide, followed by three washes with brine. The combined organic layers were dried over magnesium sulfate, and the solvent was evaporated in vacuo.

Manual preparations

All reagent flasks were connected to a passive (non-controllable) nitrogen supply. The catalyst along with insoluble solid reagents were pre-loaded into reactor flasks and kept under a nitrogen blanket until the reaction was started. All subsequent steps proceeded according to the automated protocol. Solvents were degassed prior to reaction setup. To prevent contamination of the conductivity sensor probe, an in-line celite cartridge was employed to filter the palladium residue before any liquid separation steps. Products were manually collected from the rotary evaporator flask and purified using Biotage SNAP Ultra and Biotage Sfär cartridges.

ChemPU steps

1) Confirm halide (1 mmol) is in reactor.

2) Confirm boronic acid (1.1 mmol) is in reactor.

3) Add 1,4-Dioxane (10 mL) directly to reactor at t default speed with stirring at 250 RPM.

3) Add 2M Na_2CO_3 (2 mL) directly to reactor at t default speed with stirring at 250 RPM.

4) Heat/Chill reactor to 85°C for 15 h with stirring at 650 RPM.

5) Transfer all from reactor2 directly to separator at default speed, rinsing reactor2 with EtOAc (2 x 10 mL), flushing tubing after the transfer.

6) Add water (40 mL) directly to separator at default speed with stirring at 250 RPM.

7) Wash contents of separator with water (3 x 20 mL). Transfer waste phase (bottom) to waste, and product phase (top) directly to separator.

8) Wash contents of separator with 1M NaOH (4 x 20 mL). Transfer waste phase (bottom) to waste, and product phase (top) directly to separator.

9) Wash contents of separator with sat. Brine (3 x 20 mL). Transfer waste phase (top) to waste, and product phase (bottom) through magnesium sulfate to rotavap.

10) Evaporate contents of rotavap with pressure 50 mbar at temperature 40°C for 60 min.

Experimental details

N-phenylquinazolin-4-amine (AP2-1):

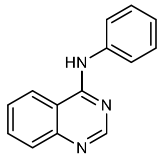

Synthesized according to general procedure (1) using 4-Chloroquinazoline (0.33 g, 2 mmol) and Aniline (0.20 mL, 2.2 mmol). The product was obtained as a white solid (0.3 g, 1.3 mmol, 67%).

^{1}H NMR (600 MHz, DMSO-*d$_6$*) δ 8.93 (s, 1H), 8.92 (d, J = 8.8 Hz, 1H), 8.12 (ddd, J = 8.4, 7.1, 1.2 Hz, 1H), 7.99 (dd, J = 8.5, 1.1 Hz, 1H), 7.88 (ddd, J = 8.3, 7.1, 1.2 Hz, 1H), 7.73 (d, J = 7.5 Hz, 2H), 7.51 (t, J = 7.6 Hz, 2H), 7.35 (t, J = 7.5 Hz, 1H).
^{13}C NMR (151 MHz, DMSO-*d$_6$*) δ 159.9, 151.1, 138.7, 136.6, 136.2, 128.8, 128.6, 126.8, 124.9, 124.8, 119.8, 113.5.
LCMS: calcd. for $C_{14}H_{11}N_3$, 222.0952[M+H$^+$]; found 222.0960.

The analytical data is in accordance with that previously reported in the literature.[2]

N-benzylquinazolin-4-amine (AP2-2):

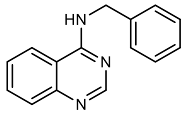

Synthesized according to general procedure (1) using 4-Chloroquinazoline (0.33 g, 2 mmol) and Benzylamine (0.24 mL, 2.2 mmol). The product was obtained as a yellow solid (0.34 g, 1.4 mmol, 72%).

^{1}H NMR (600 MHz, DMSO-*d$_6$*) δ 9.94 (s, 1H), 8.63 (s, 1H), 8.56 (d, *J* = 8.2 Hz, 1H), 7.88 (ddd, *J* = 8.4, 7.0, 1.3 Hz, 1H), 7.80 (dd, *J* = 8.3, 1.3 Hz, 1H), 7.62 (ddd, *J* = 8.2, 7.0, 1.3 Hz, 1H), 7.51 (d, *J* = 7.6 Hz, 2H), 7.31 (t, *J* = 7.4 Hz, 2H), 7.24 (t, *J* = 7.4 Hz, 1H), 4.86 (d, *J* = 4.5 Hz, 2H).
^{13}C NMR (151 MHz, DMSO-*d$_6$*) δ 159.4, 155.1, 149.2, 139.5, 134.1, 132.6, 128.9, 128.3, 127.2, 126.7, 125.7, 122.7, 114.9, 42.2.
LCMS: calcd. for $C_{15}H_{13}N_3$, 236.2900 [M+H$^+$]; found 236.7550.

The analytical data is in accordance with that previously reported in the literature.[2]

6-bromo-N-quinazolin-4-amine (AP2-3):

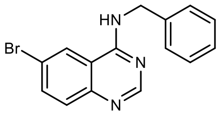

Synthesized according to general procedure (1) using 4-Chloro-6-bromoquinazoline (3 g, 12.5 mmol) and Benzylamine (1.47 mL, 13.5 mmol). The product was obtained as a bright yellow solid (2.44 g, 7.8 mmol, 62%).

^{1}H NMR (600 MHz, DMSO-*d6*) δ 9.05 (d, J = 2.0 Hz, 1H), 8.92 (s, 1H), 8.19 (dd, J = 8.9, 2.0 Hz, 1H), 7.88 (d, J = 8.9 Hz, 1H), 7.43 (d, J = 7.3 Hz, 2H), 7.35 (t, J = 7.5 Hz, 2H), 7.29 (t, J = 7.3 Hz, 1H), 4.93 (d, J = 5.8 Hz, 2H).
^{13}C NMR (151 MHz, DMSO-*d6*) δ 158.5, 155.5, 148.1, 139.0, 135.6, 129.9, 128.3, 127.6, 126.8, 125.3, 118.0, 116.2, 43.7.
LCMS: calcd. for $C_{15}H_{12}BrN_3$, 314.0215[M+H$^+$]; found 315.0390.

The analytical data is in accordance with that previously reported in the literature.[3]

N-(2-(1H-indol-3-yl)ethyl)-6-bromoquinazolin-4-amine (AP2-7):

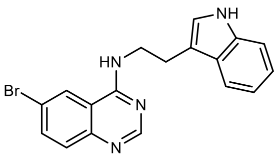

Synthesized according to general procedure (1) using 4-Chloro-6-bromoquinazoline (2.42 g, 10 mmol) and Tryptamine (1.76 g, 11 mmol). The product was obtained as a bright orange solid (2.56 g, 7 mmol, 70%).

^{1}H NMR (600 MHz, DMSO-*d6*) δ 8.98 (d, *J* = 2.1 Hz, 1H), 8.93 (s, 1H), 8.17 (dd, *J* = 8.9, 2.0 Hz, 1H), 7.87 (d, *J* = 8.8 Hz, 1H), 7.61 (d, *J* = 7.8 Hz, 1H), 7.35 (d, *J* = 8.2 Hz, 1H), 7.25 (d, *J* = 2.3 Hz, 1H), 7.07 (t, J = 8.1 Hz, 1H), 6.97 (t, *J* = 7.4 Hz, 1H), 4.00 – 3.94 (m, 2H), 3.13 (t, *J* = 7.5 Hz, 2H).
^{13}C NMR (151 MHz, DMSO-*d6*) δ 151.2, 138.4, 137.0, 136.3, 127.1, 127.0, 123.3, 123.0, 121.0, 120.4, 118.3, 118.2, 114.6, 111.5, 110.9, 109.5, 42.7, 24.0.
LCMS: calcd. for $C_{18}H_{15}BrN_4$, 367.0480 [M+H$^+$]; found 367.0669.

N-benzyl-6-phenylquinazolin-4-amine (AP2-8):

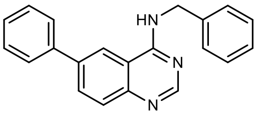

Synthesized according to general procedure (2) using N-benzyl-6-bromoquinazolin-4-amine (310 mg, 1 mmol) and Phenylboronic acid (180 mg, 1.5 mmol). The product was obtained as a white solid.

^{1}H NMR (600 MHz, DMSO-*d*$_6$) δ 8.99 (t, *J* = 5.9 Hz, 1H), 8.66 (d, *J* = 2.0 Hz, 1H), 8.46 (s, 1H), 8.13 (dd, *J* = 8.7, 2.0 Hz, 1H), 7.85 (d, *J* = 7.7 Hz, 2H), 7.77 (d, *J* = 8.6 Hz, 1H), 7.52 (t, *J* = 7.7 Hz, 2H), 7.41 (t, *J* = 7.7 Hz, 1H), 7.40 (d, *J* = 7.4 Hz, 2H), 7.33 (t, *J* = 7.6 Hz, 2H), 7.24 (t, *J* = 7.4 Hz, 1H), 4.83 (d, *J* = 5.8 Hz, 2H).

^{13}C NMR (151 MHz, DMSO-d$_6$) δ 159.5, 155.1, 148.6, 139.4, 139.1, 137.3, 131.2, 129.0, 128.3, 128.1, 127.8, 127.3, 126.9, 126.8, 120.3, 115.1, 43.6.

LCMS: calcd. for $C_{21}H_{17}N_3$, 312.1422 [M+H$^+$]; found 312.1576.

6-bromo-N-(3,4-dimethoxyphenethyl)quinazolin-4-amine (AP2-81):

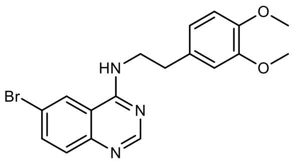

Synthesized according to general procedure (1) using 4-Chloro-6-bromoquinazoline (1.21 g, 5 mmol) and Homoveratrylamine (0.91 mL, 5.5 mmol). Product was obtained as a white solid (1.91 mmol, 0.74 g, 38%).

^{1}H NMR (600 MHz, DMSO-*d*$_6$) δ 9.99 (s, 1H), 8.85 (d, *J* = 2.2 Hz, 1H), 8.79 (s, 1H), 8.09 (dd, *J* = 8.8, 2.1 Hz, 1H), 7.78 (d, *J* = 8.8 Hz, 1H), 6.88 – 6.84 (m, 2H), 6.77 (dd, *J* = 8.1, 2.0 Hz, 1H), 3.86 (q, *J* = 6.8 Hz, 2H), 3.70 (s, 6H), 2.94 (t, *J* = 7.3 Hz, 2H).

^{13}C NMR (151 MHz, DMSO-*d*$_6$) δ 159.2, 152.4, 148.6, 147.4, 140.1, 137.6, 131.2, 126.4, 124.0, 120.6, 119.7, 115.0, 112.6, 111.9, 55.5, 55.4, 43.1, 33.5.

LCMS: calcd. for $C_{18}H_{18}N_3O_2$, 389.2650 [M+H$^+$]; found 389.0708.

6-bromo-N-(3-(trifluoromethyl)phenyl)quinazolin-4-amine (AP2-82):

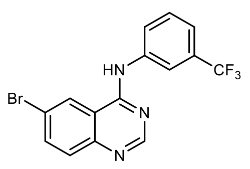

Synthesized according to general procedure (1) using 4-Chloro-6-bromoquinazoline (3 g, 12.5 mmol) and 3-(Trifluoromethyl)aniline (1.71 mL, 13.5 mmol). Product was obtained as an off-white solid (3.08 g, 8.4 mmol, 67%).

^{1}H NMR (600 MHz, DMSO-*d$_6$*) δ 9.35 (d, *J* = 2.0 Hz, 1H), 9.01 (s, 1H), 8.26 (dd, *J* = 8.9, 2.0 Hz, 1H), 8.21 (s, 1H), 8.14 (dd, *J* = 8.0, 2.0 Hz, 1H), 7.97 (d, *J* = 8.9 Hz, 1H), 7.74 (t, *J* = 7.9 Hz, 1H), 7.68 (d, *J* = 7.8 Hz, 1H).

^{13}C NMR (151 MHz, DMSO-*d$_6$*) δ 158.9, 151.6, 139.0, 138.8, 137.7, 130.0, 129.4 (q, J = 31.8 Hz), 128.1, 127.3, 123.9 (q, J = 272.0 Hz), 122.8, 121.0, 120.8 (q, J = 4.0 Hz), 115.3.

LCMS: calcd. for $C_{15}H_9BrF_3N_3$, 367.9931 [M+H$^+$]; found 368.099.

N-(3,5-bis(trifluoromethyl)phenyl)-6-bromoquinazolin-4-amine (AP2-83):

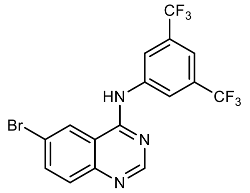

Synthesized according to general procedure (1) using 4-Chloro-6-bromoquinazoline (3 g, 12.5 mmol) and 3,5-bis (Trifluoromethyl) aniline (2.14 mL, 13.5 mmol). Product was obtained as an off-white solid (3.1 g, 7.23 mmol, 56%).

^{1}H NMR (600 MHz, DMSO-*d$_6$*) δ 9.35 (d, *J* = 2.0 Hz, 1H), 9.07 (s, 1H), 8.65 (s, 2H), 8.25 (dd, *J* = 8.9, 2.0 Hz, 1H), 8.01 (s, 1H), 7.97 (d, *J* = 8.9 Hz, 1H).

^{13}C NMR (151 MHz, DMSO-*d$_6$*) δ 158.7, 152.0, 146.5, 139.4, 138.8, 130.6 (q, J = 33.1 Hz), 128.1, 127.1, 123.6, 123.1 (q, J = 273.1 Hz), 121.0, 118.8, 115.5.

LCMS: calcd. for $C_{16}H_8BrF_6N_3$, 437.1554 [M+H$^+$]; found 437.9965.

The analytical data is in accordance with that previously reported in the literature.[4]

N-(3,4-dimethoxyphenethyl)-6-phenylquinazolin-4-amine (AP2-84):

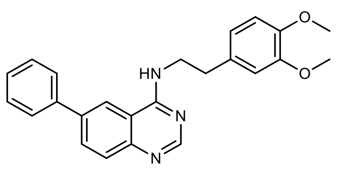

Synthesized according to general procedure (2) using 6-bromo-N-(3,4-dimethoxyphenethyl)quinazolin-4-amine (380 mg, 1 mmol) and Phenylboronic acid (120 mg, 1.5 mmol) . The product was obtained as a white solid (249 mg, 0.65 mmol, 65%).

^{1}H NMR (600 MHz, DMSO-*d$_6$*) δ 8.58 (d, *J* = 2.0 Hz, 1H), 8.52 (s, 1H), 8.08 (dd, *J* = 8.6, 2.0 Hz, 1H), 7.84 – 7.79 (m, 2H), 7.75 (d, *J* = 8.6 Hz, 1H), 7.52 (t, *J* = 7.6 Hz, 2H), 7.43 – 7.39 (m, 1H), 6.87 (d, *J* = 1.9 Hz, 1H), 6.84 (d, *J* = 8.1 Hz, 1H), 6.77 (dd, *J* = 8.2, 2.0 Hz, 1H), 3.78 (dt, *J* = 7.9, 6.0 Hz, 2H), 3.69 (d, *J* = 5.5 Hz, 6H), 2.93 (t, *J* = 7.4 Hz, 2H).
^{13}C NMR (151 MHz, DMSO-*d$_6$*) δ 159.5, 155.3, 148.6, 147.3, 139.2, 137.2, 132.0, 131.0, 129.4, 129.0, 128.1, 127.7, 126.9, 120.5, 120.2, 115.2, 112.6, 111.9, 55.5, 55.3, 42.4, 34.1.
LCMS: calcd. for $C_{24}H_{23}N_3O_2$, 386.1790 [M+H$^+$]; found 386.1445.

N-(3,5-bis(trifluoromethyl)phenyl)-6-phenylquinazolin-4-amine (AP2-86):

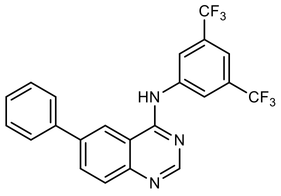

Synthesized according to general procedure (2) using N-(3,5-bis(trifluoromethyl)phenyl)-6-bromoquinazolin-4-amine (430 mg, 1 mmol) and phenylboronic acid (120 mg, 1.5 mmol). The product was obtained as a white solid (263 mg, 0.61 mmol, 61%).

^{1}H NMR (600 MHz, DMSO-*d$_6$*) δ 8.85 (d, *J* = 2.0 Hz, 1H), 8.77 (s, 1H), 8.75 (s, 2H), 8.25 (dd, *J* = 8.7, 1.9 Hz, 1H), 7.94 (d, *J* = 8.6 Hz, 1H), 7.89 (d, J = 7.6 Hz, 2H), 7.80 (s, 1H), 7.58 (t, J = 7.6 Hz, 2H), 7.48 (t, J = 7.6 Hz, 1H).
^{13}C NMR (151 MHz, DMSO-*d$_6$*) δ 157.5, 153.9, 149.1, 141.4, 140.2, 139.1, 138.7, 132.4,130.5 (q, J = 32.7 Hz), 129.1, 128.9, 128.6, 128.1, 127.4, 127.2, 126.7, 123.4 (q, J = 272.9 Hz), 121.2, 120.4, 115.8 (p), 115.3.
LCMS: calcd. for $C_{22}H_{13}F_6N_3$, 434.1014 [M+H$^+$]; found 434.1201.

N-(3,5-bis(trifluoromethyl)phenyl)-6-(4-methoxyphenyl)quinazolin-4-amine (AP2-89):

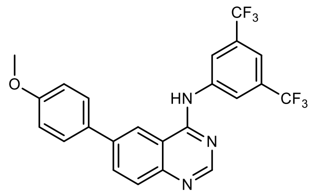

Synthesized according to general procedure (2) using N-(3,5-bis(trifluoromethyl)phenyl)-6-bromoquinazolin-4-amine (430 mg, 1 mmol) and 4-Methoxyphenyl boronic acid (150 mg, 1.5 mmol). The product was obtained as a white solid (357 mg, 0.77 mmol, 77%).

^{1}H NMR (600 MHz, DMSO-*d₆*) δ 10.30 (s, 1H), 8.78 (d, *J* = 2.0 Hz, 1H), 8.76 (s, 2H), 8.75 (s, 1H), 8.22 (dd, *J* = 8.7, 1.9 Hz, 1H), 7.91 (d, *J* = 8.6 Hz, 1H), 7.85 (d, *J* = 8.7 Hz, 2H), 7.80 (s, 1H), 7.14 (d, *J* = 8.8 Hz, 2H), 3.85 (s, 3H).
^{13}C NMR (151 MHz, DMSO-*d₆*) δ 159.4, 157.3, 153.6, 148.8, 141.5, 138.4, 132.1, 131.3, 130.5 (q, J = 32.8 Hz), 128.5, 128.4, 123.4 (q, J = 272.8 Hz), 121.2, 119.3, 115.8(d, J = 4.0 Hz), 115.4, 114.5, 55.3.
LCMS: calcd. for $C_{23}H_{15}F_6N_3O$, 464.1119 [M+H$^+$]; found 464.1326.

4-((6-bromoquinazolin-4-yl)amino)benzoic acid (AP2-94):

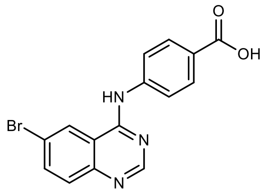

Synthesized according to general procedure (1) using 4-Chloro-6-bromoquinazoline (3 g, 12.5 mmol) and 4-Aminobenzoic acid (1.85 g, 13.5 mmol). Product was obtained as an off-white solid (4.09 g, 11.8 mmol, 95%).

^{1}H NMR (600 MHz, DMSO-*d₆*) δ 9.34 (d, *J* = 2.1 Hz, 1H), 9.01 (s, 1H), 8.25 (dd, *J* = 8.9, 2.0 Hz, 1H), 8.04 (d, *J* = 8.6 Hz, 2H), 7.98 (d, *J* = 8.9 Hz, 1H), 7.96 (d, *J* = 8.4 Hz, 2H).
^{13}C NMR (151 MHz, DMSO-*d₆*) δ 166.7, 158.7, 151.5, 140.8, 139.0, 138.8, 129.9, 128.2, 127.3, 123.8, 122.8, 121.0, 115.4.
LCMS: calcd. for $C_{15}H_{10}BrN_3O_2$, 341.9956[M-H$^+$]; found 343.9372.

N-benzyl-6-(4-fluorophenyl)quinazolin-4-amine (AP2-99):

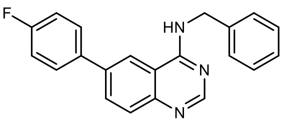

Synthesized according to general procedure (2) using N-benzyl-6-bromoquinazolin-4-amine (310 mg, 1 mmol) and 4-Fluorophenylboronic acid (140 mg, 1.5 mmol). The product was obtained as a white solid (150 mg, 0.46 mmol, 46%).

^{1}H NMR (600 MHz, DMSO-*d6*) δ 9.10 (d, J = 1.9 Hz, 1H), 8.92 (s, 1H), 8.39 (dd, J = 8.7, 1.8 Hz, 1H), 8.01 – 7.95 (m, 2H), 8.01 – 7.95 (m, 1H), 7.47 (d, *J* = 7.6 Hz, 2H), 7.37 (t, J = 14.8 Hz, 2H), 7.37 (d, J = 15.1 Hz, 2H), 7.29 (t, J = 7.4 Hz, 1H), 4.99 (d, *J* = 5.8 Hz, 2H).
^{13}C NMR (151 MHz, DMSO-*d6*) δ 159.5, 155.1, 148.6, 139.4, 138.2 (d, J = 271.7 Hz), 131.2, 129.0, 128.3, 128.1, 127.8, 127.3, 126.9, 126.8, 120.3, 115.2, 115.1, 43.6.
LCMS: calcd. for $C_{21}H_{16}FN_3$, 330.1328[M+H$^+$]; found 330.1483.

N-benzyl-6-(p-tolyl)quinazolin-4-amine (AP2-100):

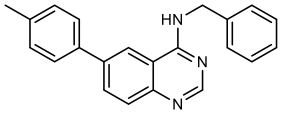

Synthesized according to general procedure (2) using N-benzyl-6-bromoquinazolin-4-amine (310 mg, 1 mmol) and p-Tolylboronic acid (140 mg, 1.5 mmol). The product was obtained as a white solid (0.11 mg, 0.34 mmol, 34%).

^{1}H NMR (600 MHz, DMSO-*d6*) δ 9.04 (s, 1H), 8.91 (s, 1H), 8.39 (dd, *J* = 8.8, 1.9 Hz, 1H), 7.97 (d, *J* = 8.7 Hz, 1H), 7.81 (d, *J* = 7.9 Hz, 2H), 7.46 (d, *J* = 7.4 Hz, 2H), 7.36 (t, *J* = 7.6 Hz, 2H), 7.35 (d, *J* = 8.1 Hz, 2H), 7.30 (t, *J* = 7.3 Hz, 1H), 4.99 (d, *J* = 5.8 Hz, 2H), 2.37 (s, 3H).
^{13}C NMR (151 MHz, DMSO-*d6*) δ 160.6, 150.7, 139.9, 138.2, 137.2, 136.6, 134.8, 134.1, 129.7, 128.5, 127.7, 127.4, 126.9, 121.2, 119.9, 113.5, 44.8, 20.7.
LCMS: calcd. for $C_{22}H_{19}N_3$, 326.1579[M+H$^+$]; found 326.1736.

N-benzyl-6-(4-methoxyphenyl)quinazolin-4-amine (AP2-101):

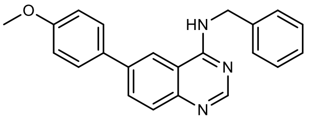

Synthesized according to general procedure (2) using 6-bromo-N-(4-methoxyphenyl)quinazolin-4-amine (310 mg, 1 mmol) and 4-methoxyphenylboronic acid (170 mg, 1.1 mmol) . The product was obtained as a white solid (210 mg, 0.61 mmol, 61%).

^{1}H NMR (600 MHz, DMSO-*d$_6$*) δ 8.60 (d, J = 2.1 Hz, 1H), 8.44 (s, 1H), 8.08 (dd, *J* = 8.7, 2.0 Hz, 1H), 7.79 (d, J = 8.7 Hz, 2H), 7.74 (d, J = 8.7 Hz, 1H), 7.40 (d, J = 7.1 Hz, 2H), 7.32 (t, *J* = 7.6 Hz, 2H), 7.24 (t, J = 7.2 Hz, 1H), 7.08 (d, *J* = 8.8 Hz, 2H), 4.83 (d, *J* = 5.8 Hz, 2H), 3.82 (s, 3H).
^{13}C NMR (151 MHz, DMSO-*d$_6$*) δ 159.4, 159.2, 154.8, 148.1, 139.4, 137.1, 131.5, 130.9, 128.3, 128.0, 128.0, 127.3, 126.8, 119.3, 115.2, 114.4, 55.2, 43.6.
LCMS: calcd. for $C_{22}H_{19}N_3O$, 342.1562 [M+H$^+$]; found 342.1679.

N-benzyl-6-(4-(trifluoromethyl)phenyl)quinazolin-4-amine (AP2-102):

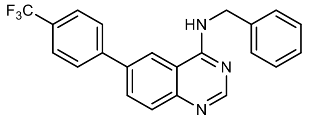

Synthesized according to general procedure (2) using 6-bromo-N-(4-methoxyphenyl)quinazolin-4-amine (310 mg, 1 mmol) and 4-(Trifluoromethyl)phenylboronic acid (280 mg, 1.5 mmol). The product was obtained as a white solid (124 mg, 0.32 mmol, 32%).

^{1}H NMR (600 MHz, DMSO-*d$_6$*) δ 8.76 (d, J = 2.0 Hz, 1H), 8.49 (s, 1H), 8.18 (dd, J = 8.6, 2.0 Hz, 1H), 8.07 (d, J = 8.1 Hz, 2H), 7.88 (d, J = 8.3 Hz, 2H), 7.81 (d, J = 8.7 Hz, 1H), 7.40 (d, J = 7.6 Hz, 2H), 7.33 (t, J = 7.5 Hz, 2H), 7.25 (t, J = 7.3 Hz, 1H), 4.84 (d, J = 5.7 Hz, 2H).
^{13}C NMR (151 MHz, DMSO-*d$_6$*) δ 159.6, 155.6, 149.1, 143.1, 139.2, 135.6, 131.2, 128.3, 127.6, 127.3, 126.8, 125.9, 125.8, 125.8 (q, *J* = 3.8 Hz), 125.8, 125.8, 121.2, 115.1, 43.6.
LCMS: calcd. for $C_{22}H_{16}F_3N_3$, 380.1296 [M+H$^+$]; found 380.1456.

Methyl-4-((6-bromoquinazolin-4-yl)amino)benzoate (AP3-11):

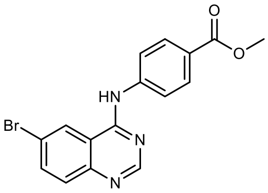

Synthesized according to general procedure (1) using 4-Chloro-6-bromoquinazoline (1.21 g, 5 mmol) and Methyl 4-aminobenzoate (0.83 g, 5.5 mmol). Product was obtained as a white solid (1.70 g, 4.7 mmol, 95%).

^{1}H NMR (600 MHz, DMSO-*d6*) δ 9.29 (d, *J* = 2.1 Hz, 1H), 9.00 (s, 1H), 8.24 (dd, *J* = 8.8, 2.0 Hz, 1H), 8.06 (d, *J* = 8.5 Hz, 2H), 8.01 (d, *J* = 8.5 Hz, 2H), 7.96 (d, *J* = 8.9 Hz, 1H), 3.88 (s, 3H).

^{13}C NMR (151 MHz, DMSO-*d6*) δ 165.7, 158.5, 151.7, 141.4, 139.8, 138.7, 129.8, 127.1, 126.7, 123.6, 123.3, 120.9, 115.5, 52.2.

LCMS: calcd. for $C_{16}H_{12}BrN_3O_2$, 357.0112 [M+H$^+$]; found 357.9782.

6-bromo-N-(4-(trifluoromethyl)phenyl)quinazolin-4-amine (AP3-12):

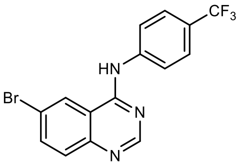

Product synthesized according to general procedure (1) using 4-Chloro-6-bromoquinazoline (1.21 g, 5 mmol) and 4-(Trifluoromethyl)aniline (0.69 mL, 5.5 mmol). Product was obtained as a white solid (1.48 g, 4 mmol, 80%).

^{1}H NMR (600 MHz, DMSO-*d6*) δ 9.35 (d, *J* = 2.1 Hz, 1H), 9.00 (s, 1H), 8.25 (dd, *J* = 8.9, 2.0 Hz, 1H), 8.06 (d, *J* = 8.4 Hz, 2H), 7.98 (d, *J* = 8.9 Hz, 1H), 7.86 (d, *J* = 8.5 Hz, 2H).

^{13}C NMR (151 MHz, DMSO-*d6*) δ 158.8, 151.6, 140.6, 139.4, 138.8, 127.2, 126.0, 125.9 (q, J = 3.8 Hz), 124.5, 124.3 (q, J = 272.6 Hz), 123.0, 121.0, 115.4.

LCMS: calcd. for $C_{15}H_9BrF_3N_3$, 367.9931 [M+H$^+$]; found 367.9588.

N-[4-methyl-3-(trifluoromethyl)phenyl]-6-bromoquinazolin-4-amine (AP4-2):

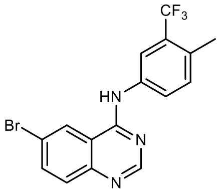

Synthesized according to general procedure (1) using 4-Chloro-6-bromoquinazoline (1.22 g, 5 mmol) and 4-methyl-3-(trifluoromethyl)aniline (0.88 g, 5.5 mmol). The product was obtained as a white solid (200 mg, 0.54 mmol, 10%).

^{1}H NMR (600 MHz, DMSO-*d6*) δ 9.16 (s, 1H), 8.96 (s, 1H), 8.22 (d, *J* = 8.9 Hz, 1H), 8.14 (s, 1H), 8.01 (d, *J* = 8.3 Hz, 1H), 7.89 (d, *J* = 8.9 Hz, 1H), 7.56 (d, *J* = 8.2 Hz, 1H), 2.48 (s, 3H).

^{13}C NMR (151 MHz, DMSO-*d6*) δ 158.5, 152.1, 140.1, 138.5, 135.3, 133.6, 133.1, 132.7, 127.5, 126.9, 124.1 (q, *J* = 274.5 Hz), 121.5, 121.1, 120.7, 115.4, 18.4.

N-(3-fluoro-5-(trifluoromethyl)phenyl)quinazolin-4-amine (AP4-3):

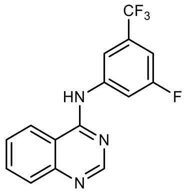

Synthesized according to general procedure (1) using 4-Chloroquinazoline (0.82 g, 5 mmol) and 4-Amino-3-fluorobenzotrifluoride (0.90 g, 5.5 mmol). The product was obtained as a white solid (200 mg, 0.67 mmol, 12%).

^{1}H NMR (600 MHz, DMSO-*d6*) δ 9.07 (s, 1H), 9.00 (d, *J* = 8.4 Hz, 1H), 8.20 (d, *J* = 9.6 Hz, 1H), 8.15 (s, 1H), 8.14 (d, *J* = 7.6 Hz, 1H), 8.02 (d, *J* = 8.3 Hz, 1H), 7.93 – 7.89 (m, 1H), 7.63 (d, *J* = 8.3 Hz, 1H).

^{13}C NMR (151 MHz, DMSO-*d6*) δ 162.6, 160.9, 159.9, 151.5, 139.9 (d, *J* = 11.0 Hz), 136.3, 131.0 (d, *J* = 9.5 Hz), 130.8 (d, *J* = 9.5 Hz), 128.7, 124.8, 124.1, 122.3, 120.8, 116.9, 115.1 (d, *J* = 25.4 Hz), 113.9, 110.0 (d, *J* = 21.1 Hz).

LCMS: calcd. for $C_{15}H_9F_4N_3$, 308.2516 [M+H$^+$]; found 308.1091 Da.

N-(2-fluoro-4-(trifluoromethyl)phenyl)quinazolin-4-amine (AP4-5):

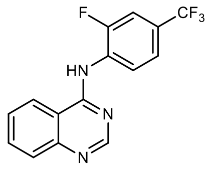

Synthesized according to general procedure (1) using 4-Chloroquinazoline (0.82 g, 5 mmol) and 3-amino-5-fluorobenzotrifluoride (0.90 g, 5.5 mmol) . The product was obtained as a white solid (237 mg, 0.77 mmol, 15%).

^{1}H NMR (600 MHz, DMSO-*d_6*) δ 8.98 (d, *J* = 8.4 Hz, 1H), 8.96 (s, 1H), 8.16 (dt, *J* = 108.1, 7.7 Hz, 1H), 8.05 (d, *J* = 8.4 Hz, 1H), 7.94 (d, *J* = 8.5 Hz, 1H), 7.91 (t, *J* = 7.6 Hz, 1H), 7.85 (t, *J* = 7.8 Hz, 1H), 7.76 (d, *J* = 8.3 Hz, 1H).

^{13}C NMR (151 MHz, DMSO-*d_6*) δ 160.7, 157.4, 155.7, 151.2, 139.2, 136.7, 129.7, 129.3 (dd, *J* = 33.2, 7.5 Hz), 129.0, 125.1, 123.2 (d, *J* = 273.8 Hz), 121.9 (t, *J* = 3.90 Hz), 120.2, 114.1 (dd, *J* = 23.7, 3.8 Hz), 113.3.

LCMS: calcd. for $C_{15}H_9F_4N_3$, 308.2516 $[M+H^+]$; found 308.1087.

N-(2-fluoro-4-(trifluoromethyl)phenyl)-6-bromoquinazolin-4-amine (AP4-6):

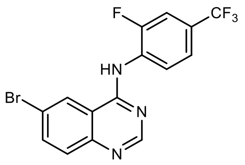

Synthesized according to general procedure (1) using 4-Chloro-6-bromoquinazoline (1.22 g, 5 mmol) and 3-amino-5-fluorobenzotrifluoride (0.90 g, 5.5 mmol) . The product was obtained as a white solid (226 mg, 0.58 mmol, 12%).

^{1}H NMR (600 MHz, DMSO-*d_6*) δ 9.27 (s, 1H), 8.95 (s, 1H), 8.28 (d, *J* = 9.0 Hz, 1H), 7.99 (d, *J* = 8.9 Hz, 1H), 7.91 (d, *J* = 9.9 Hz, 1H), 7.83 (t, *J* = 7.8 Hz, 1H), 7.74 (d, *J* = 8.3 Hz, 1H).

^{13}C NMR (151 MHz, DMSO-*d_6*) δ 159.5, 157.2, 155.5, 151.7, 146.4, 139.1, 137.3, 129.4, 127.4, 123.0, 122.8 (q, *J* = 270.4 Hz), 121.9 (q, *J* = 3.4 Hz), 121.1, 115.0, 114.1 (dd, *J* = 23.6, 3.6 Hz).

LCMS: calcd. for $C_{15}H_8BrF_4N_3$, 386.1476 $[M+H^+]$; found 386.0243.

3-(6-bromoquinazolin-4-ylamino)propanoic acid (AP4-41):

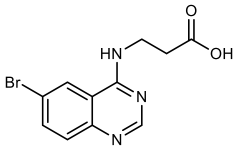

Synthesized according to general procedure (1) using 4-Chloro-6-bromoquinazoline (2.43 g, 10 mmol) and beta-alanine (1.06 g, 12 mmol). The product was obtained as a white solid (1.21 g, 4.1 mmol, 40%).

^{1}H NMR (600 MHz, DMSO-*d6*) δ 8.54 (d, *J* = 2.2 Hz, 1H), 8.50 (s, 1H), 8.44 (t, *J* = 5.3 Hz, 1H), 7.88 (dd, *J* = 8.9, 2.2 Hz, 1H), 7.62 (d, *J* = 8.8 Hz, 1H), 3.71 (q, *J* = 6.9 Hz, 2H), 2.64 (t, *J* = 7.0 Hz, 2H).

^{13}C NMR (151 MHz, DMSO-*d6*) δ 172.9, 158.4, 155.5, 148.0, 135.5, 129.8, 125.2, 117.9, 116.3, 36.9, 33.0.

LCMS: calcd. for $C_{11}H_{10}BrN_3O_2$, 297.1240 [M+H$^+$], observed: 297.9956.

N-(3,5-di-tert-butylphenyl)-6-bromoquinazolin-4-amine (AP4-42):

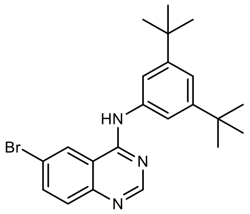

Synthesized according to general procedure (1) using 4-Chloro-6-bromoquinazoline (1.21 g, 5 mmol) and 3,5-Di-tert-butylaniline (1.23 g, 6 mmol). The product was obtained as a white solid (1.37 g, 3.3 mmol, 66%).

^{1}H NMR (600 MHz, DMSO-*d6*) δ 9.20 (s, 1H), 8.92 (s, 1H), 8.23 (dd, *J* = 8.9, 2.0 Hz, 1H), 7.92 (d, *J* = 8.9 Hz, 1H), 7.60 (d, *J* = 1.7 Hz, 2H), 7.37 (t, *J* = 1.7 Hz, 1H), 1.33 (s, 17H).

^{13}C NMR (151 MHz, DMSO-*d6*) δ 158.6, 151.4, 150.9, 138.6, 136.0, 127.1, 122.4, 120.7, 120.3, 118.8, 117.2, 115.1, 34.7, 31.2.

LCMS: calcd. for $C_{22}H_{26}BrN_3$, 412.1310 [M+H$^+$], observed: 412.1315.

N-(4-methyl-3-(trifluoromethyl)phenyl)-6-bromoquinazolin-4-amine (AP4-43):

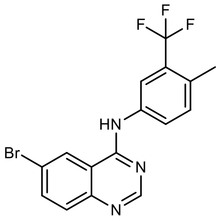

Synthesized according to general procedure (1) using 4-Chloro-6-bromoquinazoline (1.21 g, 5 mmol) and 4-Methyl-3-(trifluoromethyl)aniline (0.86 mL, 6 mmol). The product was obtained as a white solid (1.53 g, 4 mmol, 80%).

^{1}H NMR (600 MHz, DMSO-*d$_6$*) δ 11.84 (s, 1H), 9.30 (d, *J* = 2.0 Hz, 1H), 8.98 (s, 1H), 8.24 (dd, *J* = 8.8, 2.0 Hz, 1H), 8.13 (d, *J* = 2.2 Hz, 1H), 8.00 (dd, *J* = 8.3, 2.2 Hz, 1H), 7.95 (d, *J* = 8.9 Hz, 1H), 7.56 (d, *J* = 8.3 Hz, 1H), 2.48 (s, 3H).

^{13}C NMR (151 MHz, DMSO-*d$_6$*) δ 158.8, 151.5, 138.8, 135.0, 134.0, 132.7, 127.9, 127.6, 127.4, 127.2, 124.8 (q, *J* = 266.29 Hz), 122.7, 121.5, 121.5, 121.4, 121.0, 115.2, 18.4.

LCMS: calcd. for $C_{16}H_{11}BrF_3N_3$, 382.0088 [M+H$^+$], observed: 382.0080.

2-[(6-bromoquinazolin-4-yl)amino]-5-fluoro-4-(trifluoromethyl)benzoic acid (AP4-44):

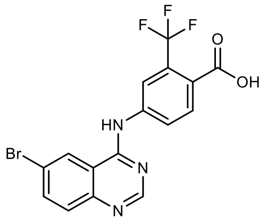

Synthesized according to general procedure (1) using 4-Chloro-6-bromoquinazoline (1.21 g, 5 mmol) and 4-Amino-2-(trifluoromethyl)benzoic acid (1.23 g, 6 mmol). The product was obtained as a white solid (2.02 g, 4.91 mmol, 98%).

^{1}H NMR (600 MHz, DMSO-*d$_6$*) δ 11.89 (s, 1H), 9.34 (d, *J* = 2.0 Hz, 1H), 9.05 (s, 1H), 8.38 (d, *J* = 2.1 Hz, 1H), 8.29 (dd, *J* = 8.5, 2.1 Hz, 1H), 8.25 (dd, *J* = 8.9, 2.0 Hz, 1H), 7.99 (d, *J* = 3.8 Hz, 1H), 7.97 (d, *J* = 4.2 Hz, 1H).

^{13}C NMR (151 MHz, DMSO-*d$_6$*) δ 167.0, 158.8, 151.8, 139.7, 138.8, 131.1, 128.8, 128.1, 127.4 (q, *J* = 31.8 Hz), 127.2, 127.0, 123.4, 123.2 (d, *J* = 269.9 Hz), 121.7 (d, *J* = 5.9 Hz), 121.0, 115.5.

LCMS: calcd. for $C_{16}H_{11}BrF_3N_3$, 411.9830 [M+H$^+$], observed: 411.9827.

6,7-dimethoxy-N-(3,4-difluorobenzyl)quinazolin-4-amine (ELK-014A):

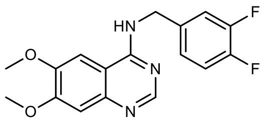

Synthesized according to general procedure (1) using 4-Chloro-6,7-dimethoxyquinazoline (0.5 g, 2.2 mmol) and 3,4-Difluorobenzylamine (0.29 mL, 2.5 mmol). The product was obtained as a yellow solid (206 mg, 0.62 mmol, 28%).

^{1}H NMR (600 MHz, DMSO-*d₆*) δ 10.17 (t, J = 5.9 Hz, 1H), 8.63 (s, 1H), 8.06 (s, 1H), 7.49 (ddd, J = 10.7, 7.9, 2.1 Hz, 1H), 7.38 (dt, J = 10.9, 8.5 Hz, 1H), 7.24 (s, 1H), 4.82 (d, J = 5.8 Hz, 2H), 3.93 (d, J = 7.0 Hz, 6H).

^{13}C NMR (151 MHz, DMSO-d_6) δ 158.8, 155.2, 150.3, 150.1 (d, J = 12.7 Hz), 149.4, 148.4 (d, J = 13.0 Hz), 147.7 (d, J = 12.4 Hz), 138.1, 136.3 (dd, J = 5.5, 3.5 Hz), 124.3 (dd, J = 6.6, 3.5 Hz), 117.0 (dd, J = 110.3, 17.2 Hz), 107.3, 103.5, 101.9, 56.6, 56.1, 43.1.

LCMS: calcd. for $C_{17}H_{15}F_2N_3O_2$, 332.1132 [M+H$^+$], observed: 332.1330.

6,7-dimethoxy-N-(2-morpholin-4-ylethyl)quinazolin-4-amine (ELK-015A):

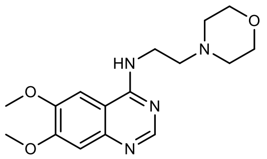

Synthesized according to general procedure (1) using 4-Chloro-6,7-dimethoxyquinazoline (0.5 g, 2.2 mmol) 4-(2-Aminoethyl)morpholine (0.319 g, 2.5 mmol). The product was obtained as a white solid (97 mg, 0.3 mmol, 14%).

^{1}H NMR (600 MHz, DMSO-d_6) δ 8.80 (s, 1H), 8.18 (s, 1H), 7.29 (s, 1H), 4.08 (q, J = 5.7 Hz, 2H), 3.97 (d, J = 7.3 Hz, 6H), 3.90 (s, 3H), 3.47 (t, J = 5.8 Hz, 2H).

^{13}C NMR (151 MHz, DMSO-d_6) δ 159.2, 155.5, 149.9, 149.6, 107.4, 103.9, 101.3, 56.8, 56.2, 55.1, 51.4, 40.1, 35.6.

LCMS: calcd. for $C_{16}H_{22}N_4O_3$, 319.1692 [M+H$^+$], observed: 319.1867.

The analytical data is in accordance with that previously reported in the literature.[5]

6,7-dimethoxy-N-(2-(4-methylpiperazin-1-yl)ethyl)quinazolin-4-amine (ELK-016A):

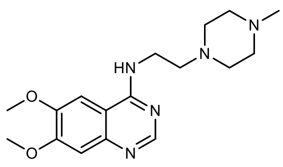

Synthesized according to general procedure (1) using 4-Chloro-6,7-dimethoxyquinazoline (0.5 g, 2.2 mmol) and 2-(4-Methyl-piperazin-1-yl)-ethylamine (0.35 g, 2.5 mmol). The product was obtained as a white solid (243 mg, 0.73 mmol, 33%).

^{1}H NMR (600 MHz, CD_3OD) δ 8.63 (s, 1H), 7.81 (s, 1H), 7.17 (s, 1H), 4.04 (d, *J* = 2.6 Hz, 6H), 3.97 (t, *J* = 6.3 Hz, 2H), 2.88 (t, *J* = 6.3 Hz, 2H), 2.85 (s, 3H).

^{13}C NMR (151 MHz, CD_3OD) δ 161.4, 158.5, 152.4, 150.0, 136.3, 108.3, 104.0, 100.7, 57.4, 57.2, 56.9, 54.8, 51.2, 43.6, 39.9.

LCMS: calcd. for $C_{17}H_{25}N_5O_2$, 332.2008 [M+H$^+$], observed: 332.2178.

6,7-bis(2-methoxyethoxy)-N-(3,4-difluorobenzyl)quinazolin-4-amine (ELK-019A):

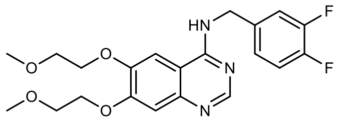

Synthesized according to general procedure (1) using 4-chloro-6,7-bis(2-methoxyethoxy)quinazoline (0.8 g, 2.5 mmol) and 3,4-Difluorobenzylamine (0.33 mL, 2.8 mmol). The product was obtained as a white solid (315 mg, 0.76 mmol, 30%).

^{1}H NMR (600 MHz, DMSO-*d*$_6$) δ 8.79 (d, *J* = 10.2 Hz, 1H), 8.07 (d, *J* = 11.3 Hz, 1H), 7.54 – 7.48 (m, 1H), 7.46 – 7.38 (m, 1H), 7.28 (s, 1H), 4.90 (d, *J* = 5.8 Hz, 2H), 4.30 (dt, *J* = 9.1, 4.7 Hz, 4H), 3.76 (dd, *J* = 5.8, 3.3 Hz, 4H), 3.35 (s, 7H).

^{13}C NMR (151 MHz, DMSO-*d*$_6$) δ 159.7, 155.7, 150.6 (d, *J* = 12.7 Hz), 150.0 (d, *J* = 12.4 Hz), 149.7, 149.6, 149.0 (d, *J* = 12.7 Hz), 148.4 (d, *J* = 12.4 Hz), 136.1, 124.9 (dd, *J* = 6.5, 3.6 Hz), 117.6 (dd, *J* = 114.0, 17.2 Hz), 107.3, 105.4, 101.3, 70.4, 70.3, 69.3, 69.2, 58.9, 58.8, 43.9.

LCMS: calcd. for $C_{21}H_{23}F_2N_3O_4$, 420.1657 [M+H$^+$], observed: 420.1862.

6,7-bis(2-methoxyethoxy)-N-(2-morpholin-4-ylethyl)quinazolin-4-amine (ELK-021A):

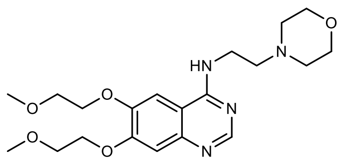

Synthesized according to general procedure (1) using 4-chloro-6,7-bis(2-methoxyethoxy)quinazoline (1 g, 3.2 mmol) and 4-(2-Aminoethyl)morpholine (0.46 mL, 3.5 mmol). The product was obtained as a white solid (255 mg, 0.63 mmol, 20%).

^{1}H NMR (600 MHz, CD$_3$OD) δ 8.48 (s, 1H), 7.65 (s, 1H), 7.10 (s, 1H), 4.30 (q, *J* = 4.0 Hz, 4H), 3.98 (t, *J* = 6.0 Hz, 2H), 3.85 (q, *J* = 4.7 Hz, 8H), 3.46 (d, *J* = 2.9 Hz, 5H), 3.19 (t, *J* = 6.1 Hz, 2H), 3.09 (t, *J* = 4.5 Hz, 4H).

^{13}C NMR (151 MHz, CD$_3$OD) δ 160.9, 156.8, 152.1, 150.8, 141.7, 109.3, 105.2, 105.0, 71.8, 71.7, 70.4, 70.1, 66.3, 59.5, 58.4, 54.1, 38.1.

LCMS: calcd. for $C_{21}H_{23}F_2N_3O_4$, 407.2216 [M+H$^+$], observed: 407.2378.

6,7-bis(2-methoxyethoxy)-N-(2-(4-methylpiperazin-1-yl)ethyl)quinazolin-4-amine (ELK-022A):

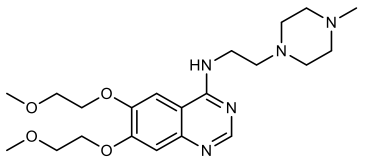

Synthesized according to general procedure (1) using 4-chloro-6,7-bis(2-methoxyethoxy)quinazoline (0.8 g, 2.5 mmol) and 2-(4-Methyl-piperazin-1-yl)-ethylamine (0.42 mL, 2.9 mmol). The product was obtained as a white solid (141 mg, 0.33 mmol, 13%).

^{1}H NMR (600 MHz, CD$_3$OD) δ 8.62 (s, 1H), 7.87 (s, 1H), 7.19 (s, 1H), 4.35 (q, *J* = 4.1 Hz, 4H), 3.96 (t, *J* = 6.2 Hz, 2H), 3.88 – 3.84 (m, 4H), 3.46 (d, *J* = 3.6 Hz, 5H), 2.88 (t, *J* = 6.3 Hz, 2H), 2.85 (s, 3H).

^{13}C NMR (151 MHz, CD$_3$OD) δ 161.3, 157.8, 151.5, 149.9, 135.8, 108.2, 105.6, 101.7, 71.7, 71.6, 70.8, 70.5, 59.5, 59.5, 56.8, 54.8, 51.1, 43.6, 39.9.

LCMS: calcd. for $C_{21}H_{33}N_5O_4$, 420.2533 [M+H$^+$], observed: 420.2690.

N-[2-(3,4-dimethoxyphenyl)ethyl]-6,7-dimethoxyquinazolin-4-amine (ELK-023A):

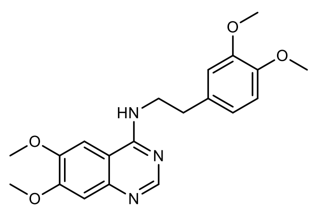

Synthesized according to general procedure (1) using 4-Chloro-6,7-dimethoxyquinazoline (1 g, 4.5 mmol) and 3,4-Dimethoxyphenethylamine (0.83 mL, 4.9 mmol). The product was obtained as an off-white solid (906 mg, 0.33 mmol, 55%).

^{1}H NMR (600 MHz, DMSO-*d$_6$*) δ 8.79 (s, 1H), 8.01 (s, 1H), 7.25 (s, 1H), 6.87 (s, 1H), 6.86 (s, 1H), 6.78 (dd, *J* = 8.1, 2.0 Hz, 1H), 3.95 (d, *J* = 10.9 Hz, 6H), 3.93 – 3.87 (m, 2H), 3.70 (d, *J* = 4.2 Hz, 6H), 2.95 (t, *J* = 7.4 Hz, 2H).

^{13}C NMR (151 MHz, DMSO-*d$_6$*) δ 158.9, 155.6, 149.7, 148.6, 147.4, 134.1, 131.2, 120.5, 112.6, 111.9, 106.6, 104.0, 99.5, 56.9, 56.3, 55.5, 55.4, 43.0, 33.8.

LCMS: calcd. for $C_{20}H_{23}N_3O_4$, 370.1689 [M+H$^+$], observed: 370.1884.

The analytical data is in accordance with that previously reported in the literature.[5]

6,7-dimethoxy-N-((thiophen-2-yl)methyl)quinazolin-4-amine (ELK-038A):

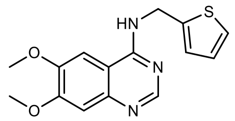

Synthesized according to general procedure (1) using 4-Chloro-6,7-dimethoxyquinazoline (0.8 g, 3.5 mmol) and 2-Thiophenemethylamine (0.40 mL, 3.9 mmol). The product was obtained as an off-white solid (383 mg, 1.27 mmol, 36%).

^{1}H NMR (600 MHz, DMSO-*d$_6$*) δ 10.75 (s, 1H), 8.84 (s, 1H), 8.12 (s, 1H), 7.42 (d, *J* = 5.0 Hz, 1H), 7.35 (s, 1H), 7.16 (d, *J* = 3.3 Hz, 1H), 6.98 (dd, *J* = 5.1, 3.5 Hz, 1H), 5.04 (d, *J* = 5.7 Hz, 2H), 3.94 (d, *J* = 4.0 Hz, 6H).

^{13}C NMR (151 MHz, DMSO-*d$_6$*) δ 158.7, 155.8, 149.8, 148.9, 140.0, 134.8, 126.8, 125.7, 106.7, 103.8, 99.8, 56.8, 56.3.

LCMS: calcd. for $C_{15}H_{15}N_3O_2S$, 302.0885 [M+H$^+$], observed: 302.1041.

N-(furan-2-ylmethyl)-6,7-dimethoxyquinazolin-4-amine (ELK-039B):

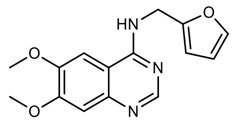

Synthesized according to general procedure (1) using 4-Chloro-6,7-dimethoxyquinazoline (0.8 g, 3.5 mmol) and Furfurylamine (2-aminomethylfuran) (0.35 mL, 3.9 mmol). The product was obtained as an off-white solid (102 mg, 0.35 mmol, 10%).

^{1}H NMR (600 MHz, DMSO-*d$_6$*) δ 8.84 (s, 1H), 8.01 (s, 1H), 7.63 (s, 1H), 7.29 (s, 1H), 6.44 (s, 1H), 4.92 (d, *J* = 5.6 Hz, 2H), 3.96 (s, 3H), 3.94 (s, 3H).

^{13}C NMR (151 MHz, DMSO-*d$_6$*) 159.0, 155.9, 150.5, 149.9, 148.9, 142.6, 134.9, 110.7, 108.2, 106.7, 103.7, 99.8, 56.7, 56.3, 37.8.

LCMS: calcd. for $C_{15}H_{15}N_3O_3$, 286.1113 [M+H$^+$], observed: 286.1277.

The analytical data is in accordance with that previously reported in the literature.[6]

6,7-dimethoxy-N-(2-(thiophen-2-yl)ethyl)quinazolin-4-amine (ELK-040C):

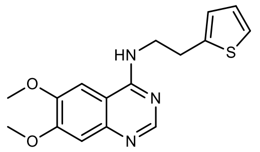

Synthesized according to general procedure (1) using 4-Chloro-6,7-dimethoxyquinazoline (0.8 g, 3.5 mmol) and 2-Thiopheneethylamine (0.48 mL, 3.9 mmol). The product was obtained as an off-white solid (203 mg, 0.64 mmol, 18%).

^{1}H NMR (600 MHz, DMSO-*d$_6$*) δ 10.17 (s, 1H), 8.79 (s, 1H), 8.05 (s, 1H), 7.36 (t, *J* = 3.5 Hz, 1H), 7.28 (s, 1H), 6.96 (d, *J* = 3.1 Hz, 2H), 3.95 (d, *J* = 4.5 Hz, 6H), 3.95 – 3.91 (m, 2H), 3.25 (t, *J* = 7.3 Hz, 2H).

^{13}C NMR (151 MHz, DMSO-*d$_6$*) δ 159.1, 155.7, 149.8, 149.0, 140.7, 134.8, 127.1, 125.5, 124.4, 106.7, 103.8, 100.0, 56.8, 56.3, 42.8, 28.5.

LCMS: calcd. for $C_{16}H_{17}N_3O_2S$, 316.1041 [M+H$^+$], observed 316.1041.

6-bromo-N-(thiophen-2-ylmethyl)quinazolin-4-amine (ELK-043C):

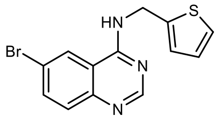

Synthesized according to general procedure (1) using 4-Chloro-6,7-dimethoxyquinazoline (0.8 g, 3.3 mmol) and 2-Thiophenemethylamine (0.37 mL, 3.6 mmol). The product was obtained as an off-white solid (884 mg, 2.76 mmol, 84%).

^{1}H NMR (600 MHz, DMSO-*d$_6$*) δ 10.45 (s, 1H), 8.87 (s, 1H), 8.84 (d, J = 2.1 Hz, 1H), 8.11 (dd, J = 8.9, 2.1 Hz, 1H), 7.80 (d, J = 8.9 Hz, 1H), 7.44 (dd, J = 5.1, 1.3 Hz, 1H), 7.16 (dd, J = 3.5, 1.1 Hz, 1H), 7.00 (dd, J = 5.1, 3.5 Hz, 1H), 5.04 (d, J = 5.5 Hz, 2H).

^{13}C NMR (151 MHz, DMSO-*d$_6$*) δ 158.9, 152.4, 140.2, 139.7, 137.8, 126.9, 126.8, 126.5, 125.8, 124.0, 120.0, 115.0, 39.5.

LCMS: calcd. for $C_{13}H_{10}BrN_3S$, 319.9779 [M+H$^+$], observed: 319.9886.

The analytical data is in accordance with that previously reported in the literature.[6]

6-bromo-N-(2-(thiophen-2-yl)ethyl)quinazolin-4-amine (ELK-046C):

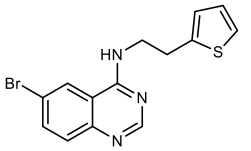

Synthesized according to general procedure (1) using 4-Chloro-6,7-dimethoxyquinazoline (0.8 g, 3.3 mmol) and 2-Thiopheneethylamine (0.44 mL, 3.6 mmol). The product was obtained as an off-white solid (374 mg, 1.11 mmol, 34%).

^{1}H NMR (600 MHz, DMSO-*d$_6$*) δ 8.93 (s, 1H), 8.88 (d, J = 2.0 Hz, 1H), 8.19 (dd, J = 8.9, 2.1 Hz, 1H), 7.82 (d, J = 8.8 Hz, 1H), 7.39 – 7.35 (m, 1H), 6.97 (s, 1H), 6.96 (s, 1H), 3.95 (td, J = 7.2, 5.5 Hz, 2H), 3.25 (t, J = 7.2 Hz, 2H).

LCMS: calcd. for $C_{14}H_{12}BrN_3S$, 334.9935 [M+H$^+$], observed: 334.0041.

The analytical data is in accordance with that previously reported in the literature.[7]

N-(thiophen-2-ylmethyl)quinazolin-4-amine (ELK-047A):

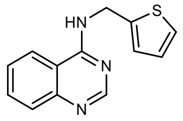

Synthesized according to general procedure (1) using 4-chloroquinazoline (0.3 g, 2 mmol) and 2-Thiophenemethylamine (0.23 mL, 2.2 mmol). The product was obtained as a yellow solid (349 mg, 1.44 mmol, 71%).

^{1}H NMR (600 MHz, DMSO-*d₆*) δ 11.14 (s, 1H), 8.98 (s, 1H), 8.68 (d, *J* = 8.3 Hz, 1H), 8.05 (t, *J* = 7.4 Hz, 1H), 7.94 (d, *J* = 8.3 Hz, 1H), 7.78 (t, *J* = 7.5 Hz, 1H), 7.45 (dd, *J* = 5.1, 1.2 Hz, 1H), 7.18 (d, *J* = 3.1 Hz, 1H), 7.00 (dd, *J* = 5.1, 3.5 Hz, 1H), 5.09 (d, *J* = 5.8 Hz, 2H).

^{13}C NMR (151 MHz, DMSO-d_6) δ 160.2, 151.1, 139.3, 137.9, 136.0, 128.5, 127.1, 126.8, 125.9, 124.5, 119.5, 113.0, 40.1.

LCMS: calcd. for $C_{13}H_{11}N_3S$, 242.0674 [M+H$^+$], observed: 242.0794.

The analytical data is in accordance with that previously reported in the literature.[7]

6-iodo-N-(thiophen-2-ylmethyl)quinazolin-4-amine (ELK-050A):

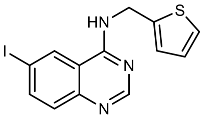

Synthesized according to general procedure (1) using 4-Chloro-6-iodoquinazoline (0.5 g, 1.72 mmol) and 2-Thiophenemethylamine (0.19 mL, 1.9 mmol). The product was obtained as a yellow solid (333 mg, 0.90 mmol, 53%).

^{1}H NMR (600 MHz, DMSO-d_6) δ 10.78 (s, 1H), 9.00 (d, *J* = 1.9 Hz, 1H), 8.99 (s, 1H), 8.31 (dd, *J* = 8.7, 1.7 Hz, 1H), 7.65 (d, *J* = 8.7 Hz, 1H), 7.47 (dd, *J* = 5.1, 1.3 Hz, 1H), 7.18 (dd, *J* = 3.5, 1.2 Hz, 1H), 7.01 (dd, *J* = 5.1, 3.5 Hz, 1H), 5.08 (d, *J* = 5.6 Hz, 2H).

^{13}C NMR (151 MHz, DMSO-d_6) δ 159.6, 151.5, 149.7, 142.9, 138.6, 137.6, 126.8, 122.2, 120.6, 114.6, 110.7, 108.7, 38.3.

LCMS: calcd. for $C_{13}H_{10}IN_3S$, 367.9640 [M+H$^+$], observed: 367.9739.

6-iodo-N-(furan-2-ylmethyl)quinazolin-4-amine (ELK-051A):

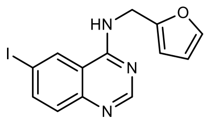

Synthesized according to general procedure (1) using 4-Chloro-6-iodoquinazoline (0.5 g, 1.72 mmol) and Furfurylamine (2-aminomethylfuran) (0.17 mL, 1.9 mmol). The product was obtained as a yellow solid (109 mg, 0.31 mmol, 18%).

^{1}H NMR (600 MHz, DMSO-*d$_6$*) δ 10.62 (s, 1H), 9.02 (d, *J* = 1.8 Hz, 1H), 8.97 (s, 1H), 8.32 (dd, *J* = 8.7, 1.7 Hz, 1H), 7.67 (d, *J* = 8.7 Hz, 1H), 7.65 (dd, *J* = 1.9, 0.9 Hz, 1H), 6.48 – 6.43 (m, 2H), 4.93 (d, *J* = 5.4 Hz, 2H).

^{13}C NMR (151 MHz, DMSO-*d$_6$*) δ 159.3, 151.2, 149.7, 144.0, 142.8, 137.5, 132.7, 121.3, 114.7, 110.7, 108.6, 93.9, 38.3.

LCMS: calcd. for $C_{13}H_{10}IN_3O$, 351.9869 [M+H$^+$], observed: 351.9982.

6-iodo-N-(2-(thiophen-2-yl)ethyl)quinazolin-4-amine (ELK-052A):

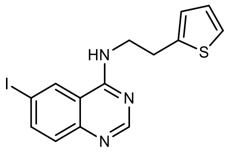

Synthesized according to general procedure (1) using 4-Chloro-6-iodoquinazoline (0.5 g, 1.72 mmol) and 2-Thiopheneethylamine (0.23 mL, 1.9 mmol). The product was obtained as a yellow solid (308 mg, 0.80 mmol, 45%).

^{1}H NMR (600 MHz, DMSO-*d$_6$*) δ 10.44 (s, 1H), 9.00 (d, *J* = 1.7 Hz, 1H), 8.92 (s, 1H), 8.31 (dd, *J* = 8.7, 1.7 Hz, 1H), 7.66 (d, *J* = 8.8 Hz, 1H), 7.39 – 7.35 (m, 1H), 6.99 – 6.94 (m, 2H), 3.98 – 3.91 (m, 2H), 3.25 (t, *J* = 7.2 Hz, 2H).

^{13}C NMR (151 MHz, DMSO-*d$_6$*) δ 159.3, 151.0, 143.9, 140.5, 137.1, 132.7, 127.1, 125.7, 124.5, 121.2, 114.7, 93.8, 43.2, 28.0.

LCMS: calcd. for $C_{14}H_{12}IN_3S$, 381.9797 [M+H$^+$], observed: 381.9911.

NMR Spectra

AP2-1 – ^{1}H NMR (600 MHz, DMSO-*d*6)

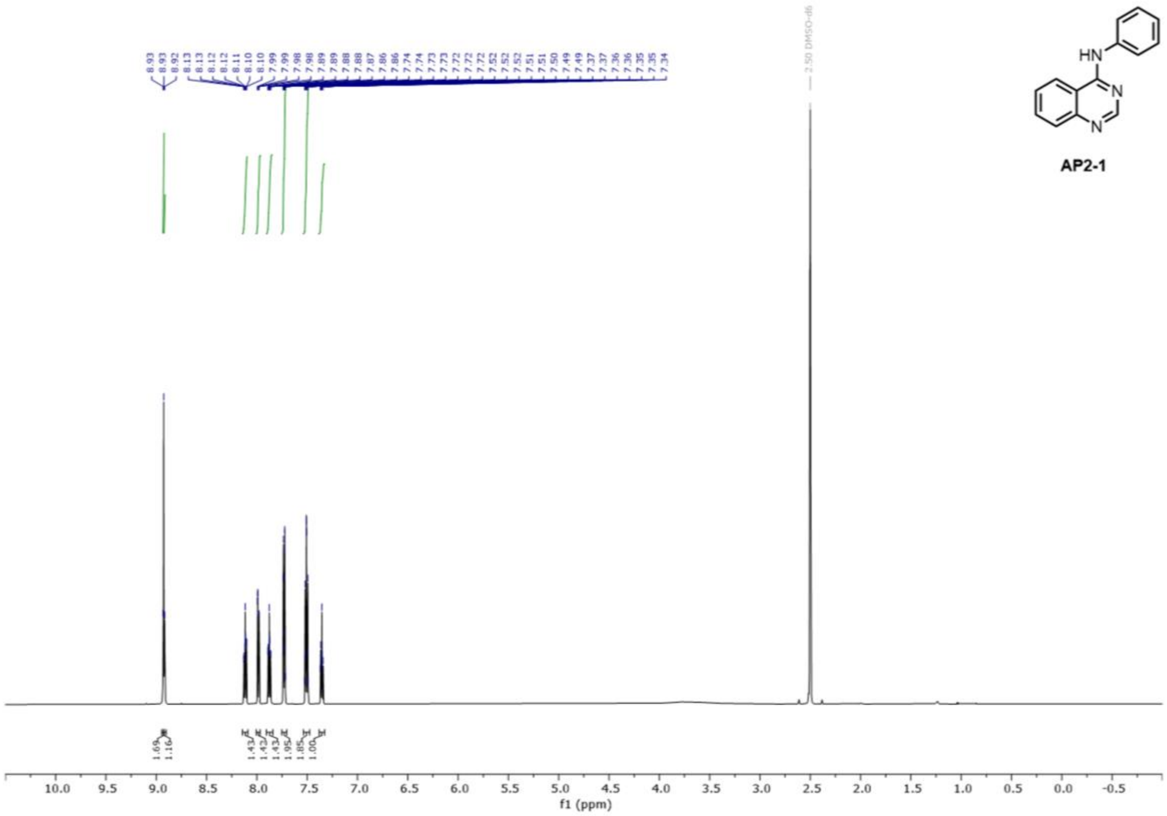

AP2-1 – ^{13}C NMR (151 MHz, DMSO-*d*6)

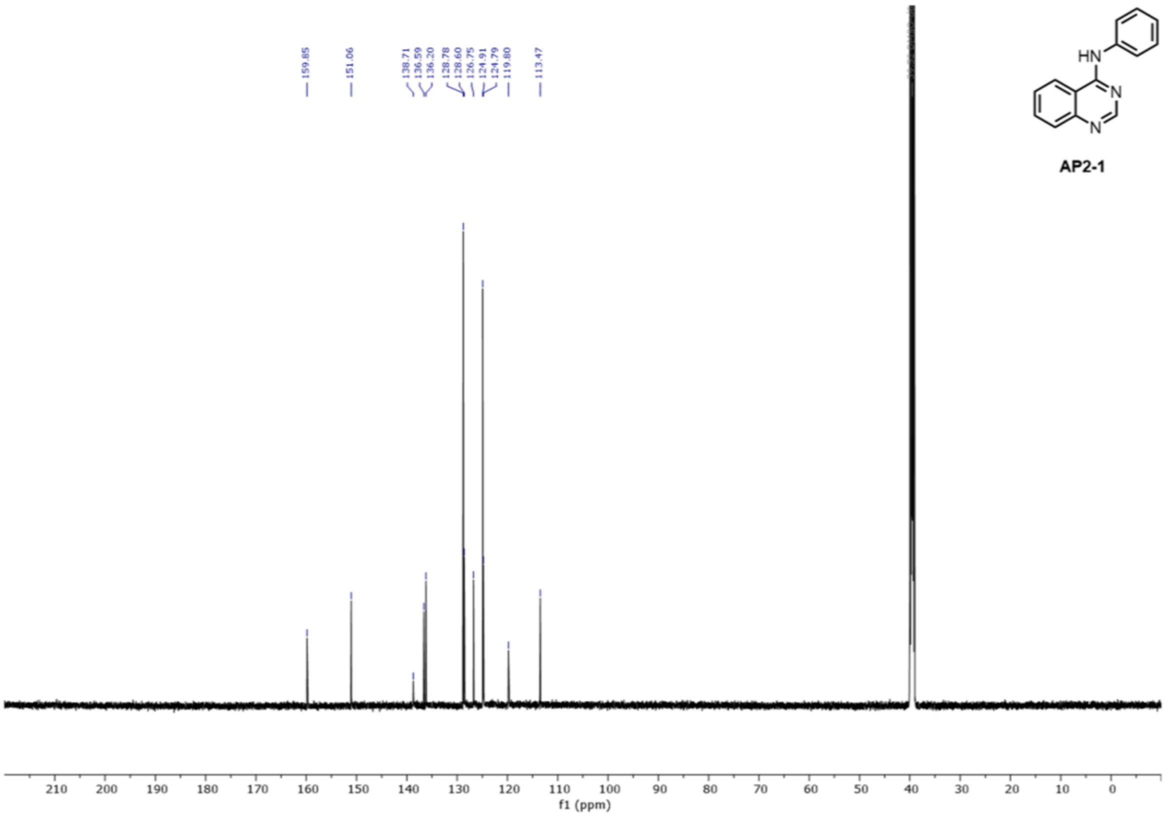

AP2-2 – ^{1}H NMR (600 MHz, DMSO-d_6)

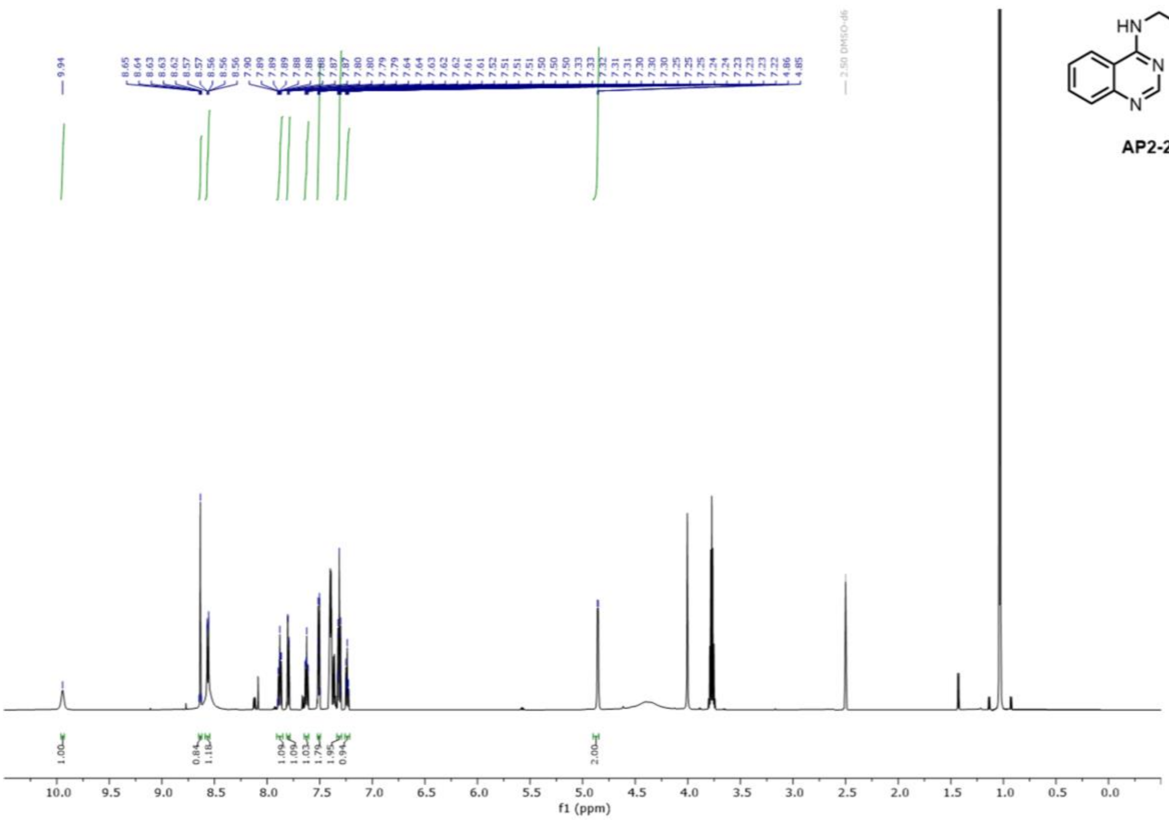

AP2-2 – ^{13}C NMR (151 MHz, DMSO-d_6)

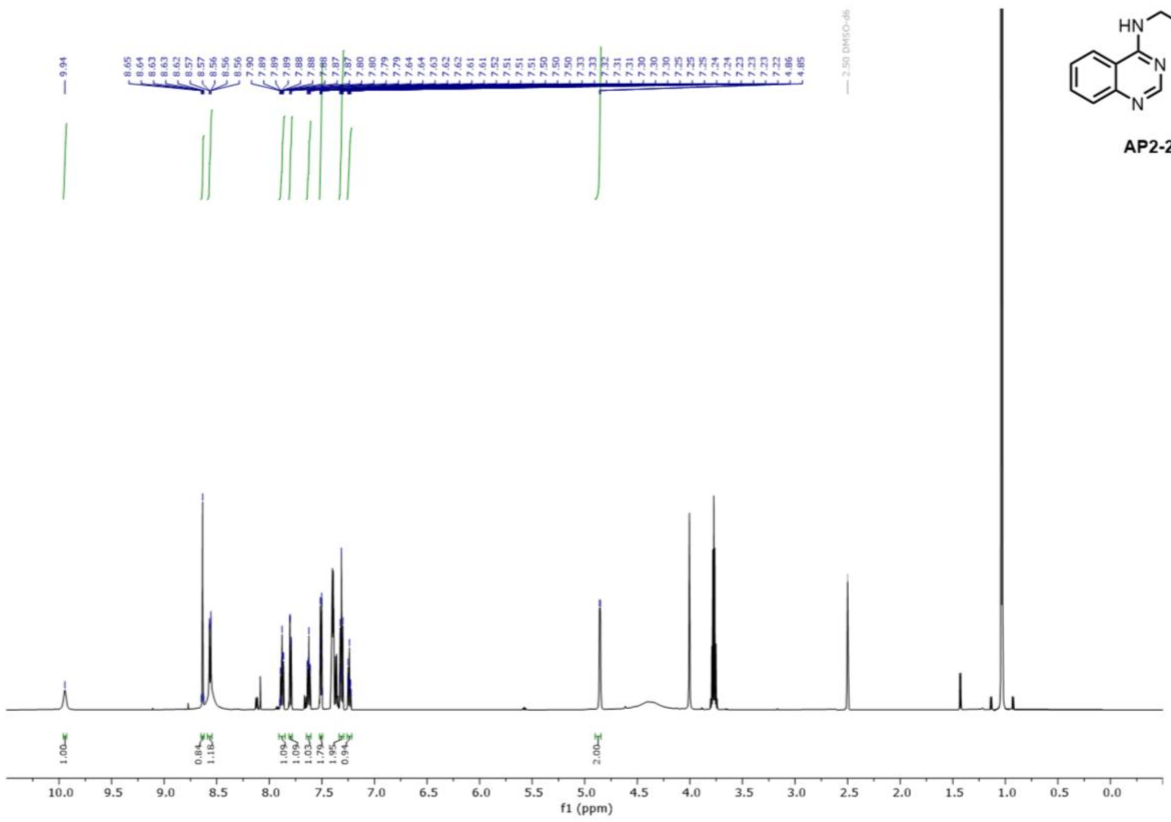

3 – ^{1}H NMR (600 MHz, DMSO-d_6)

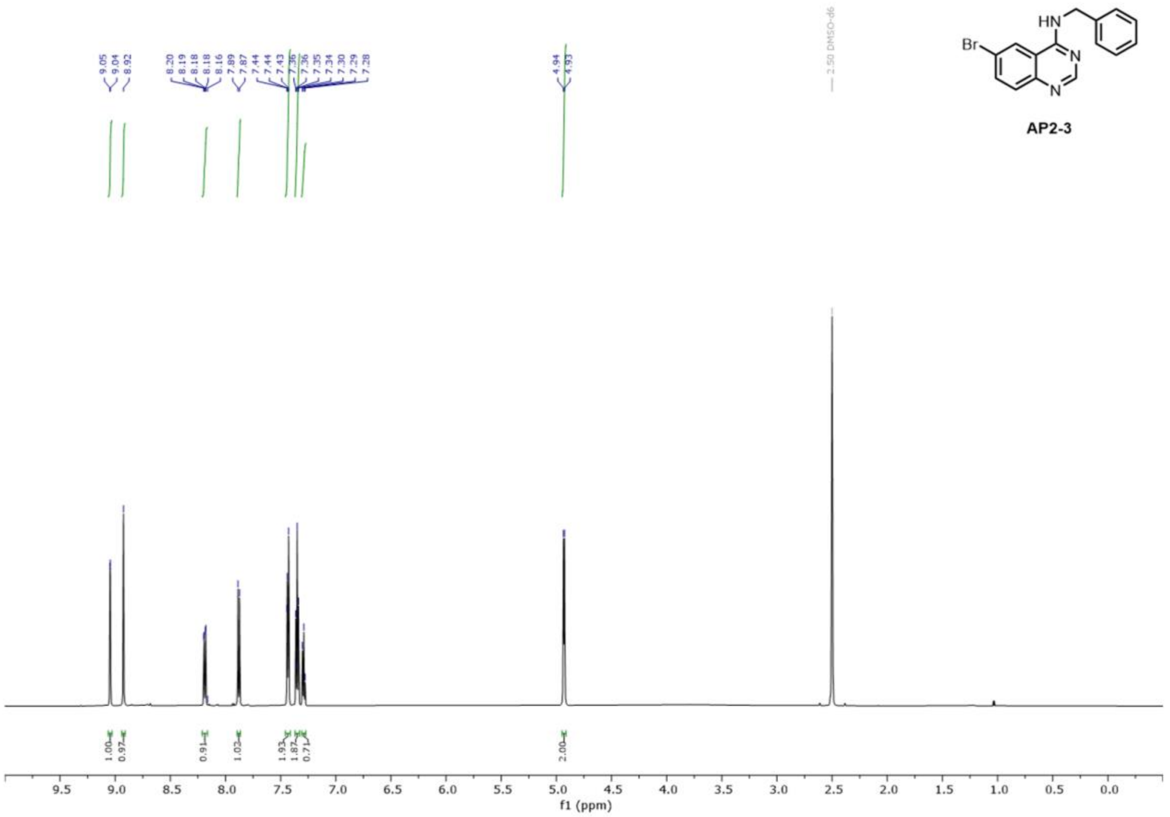

3 – ^{13}C NMR (151 MHz, DMSO-d_6)

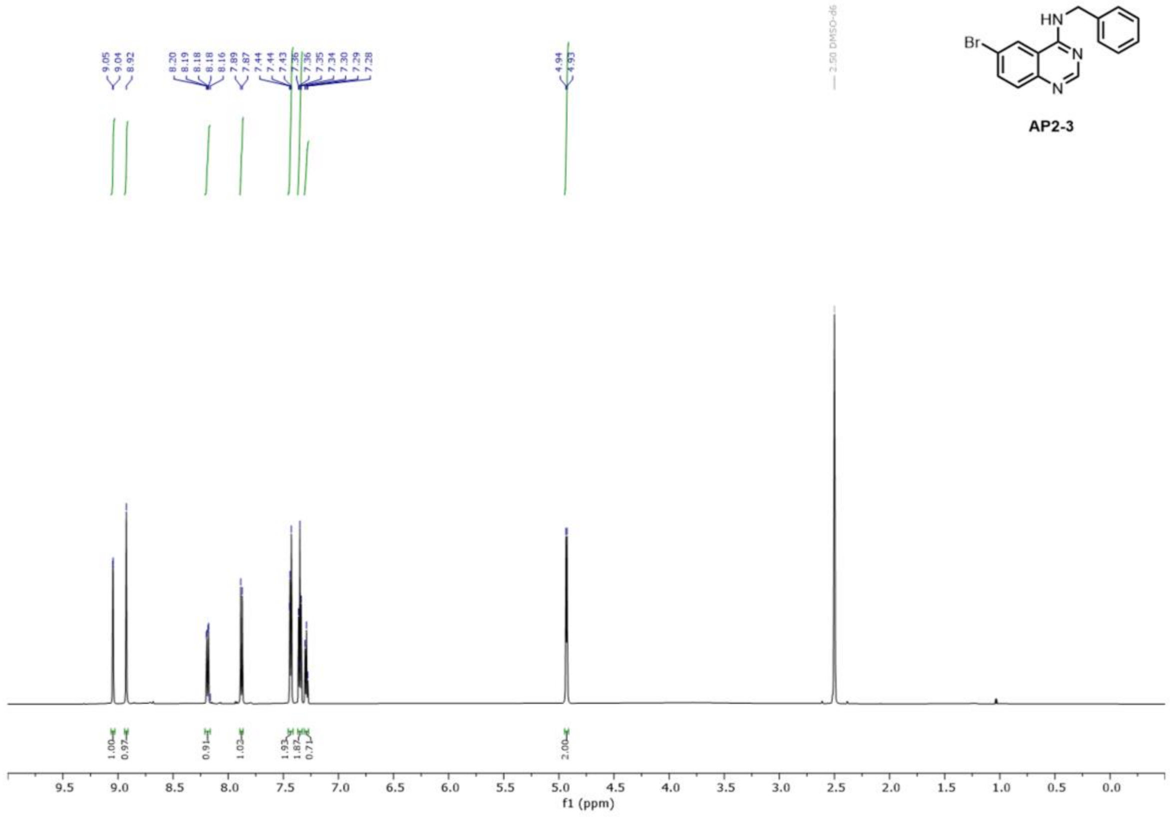

AP2-7 – ^{1}H NMR (600 MHz, DMSO-d_6)

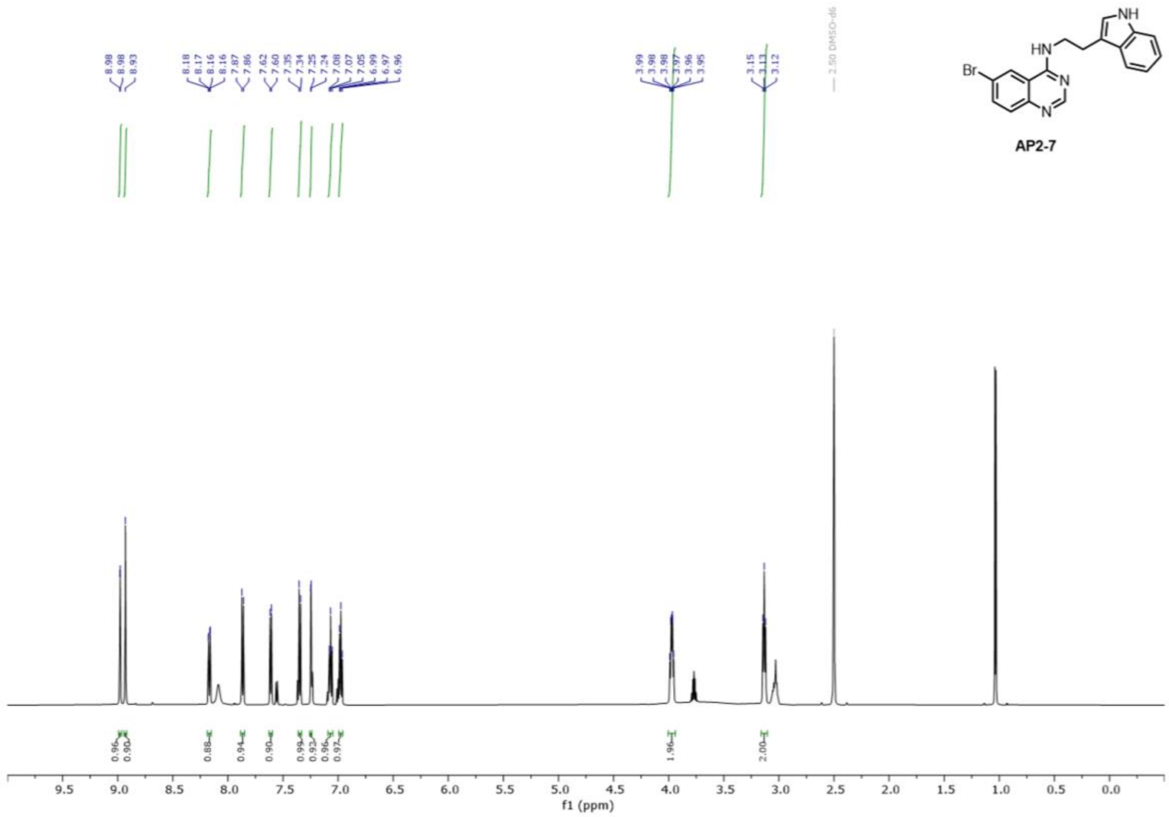

AP2-7 – ^{13}C NMR (151 MHz, DMSO-d_6)

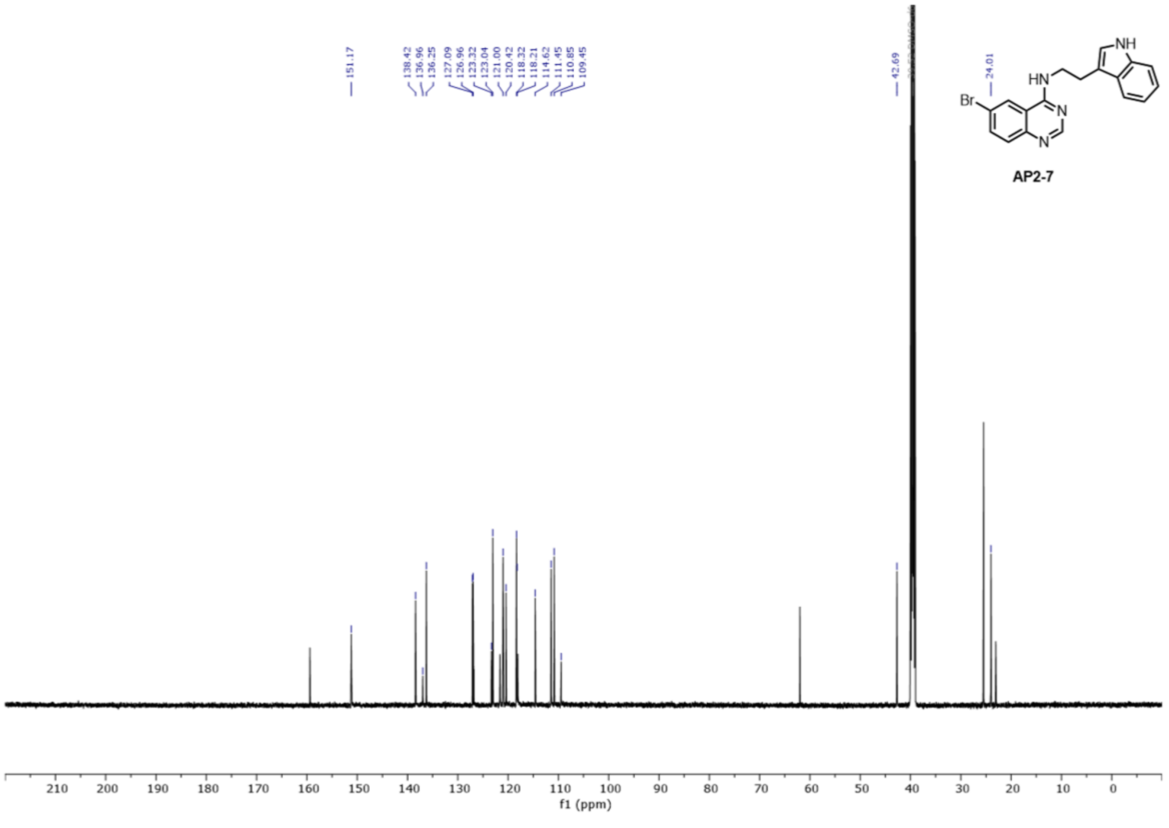

AP2-8 – ^{1}H NMR (600 MHz, DMSO-*d*$_6$)

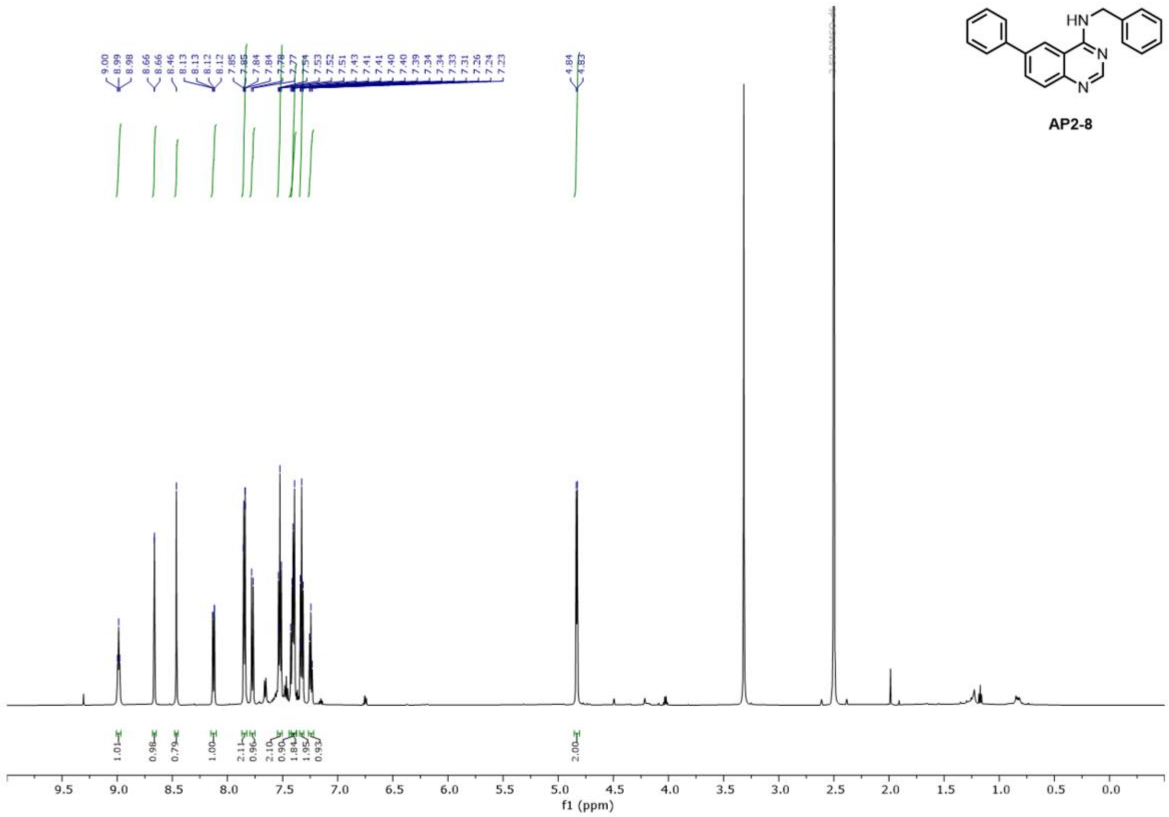

AP2-8 – ^{13}C NMR (151 MHz, DMSO-*d*$_6$)

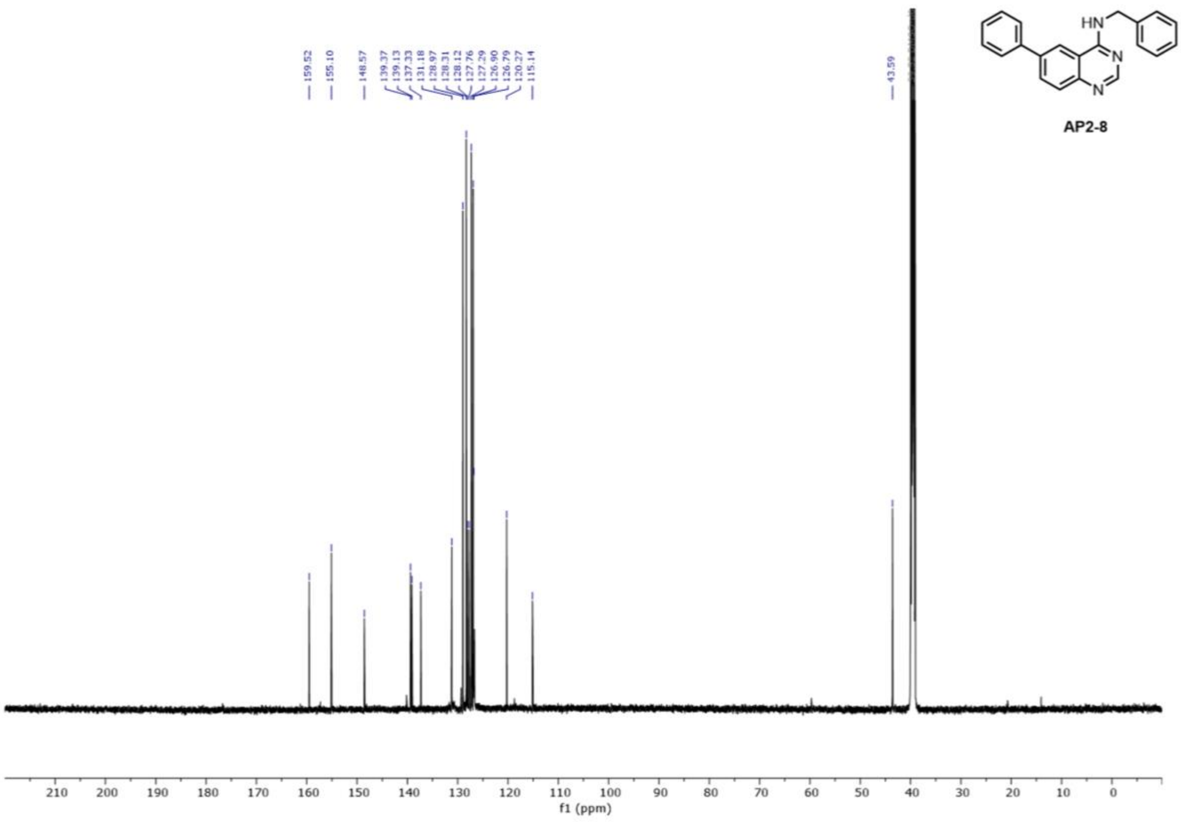

AP2-81 – ^{1}H NMR (600 MHz, DMSO-d_6)

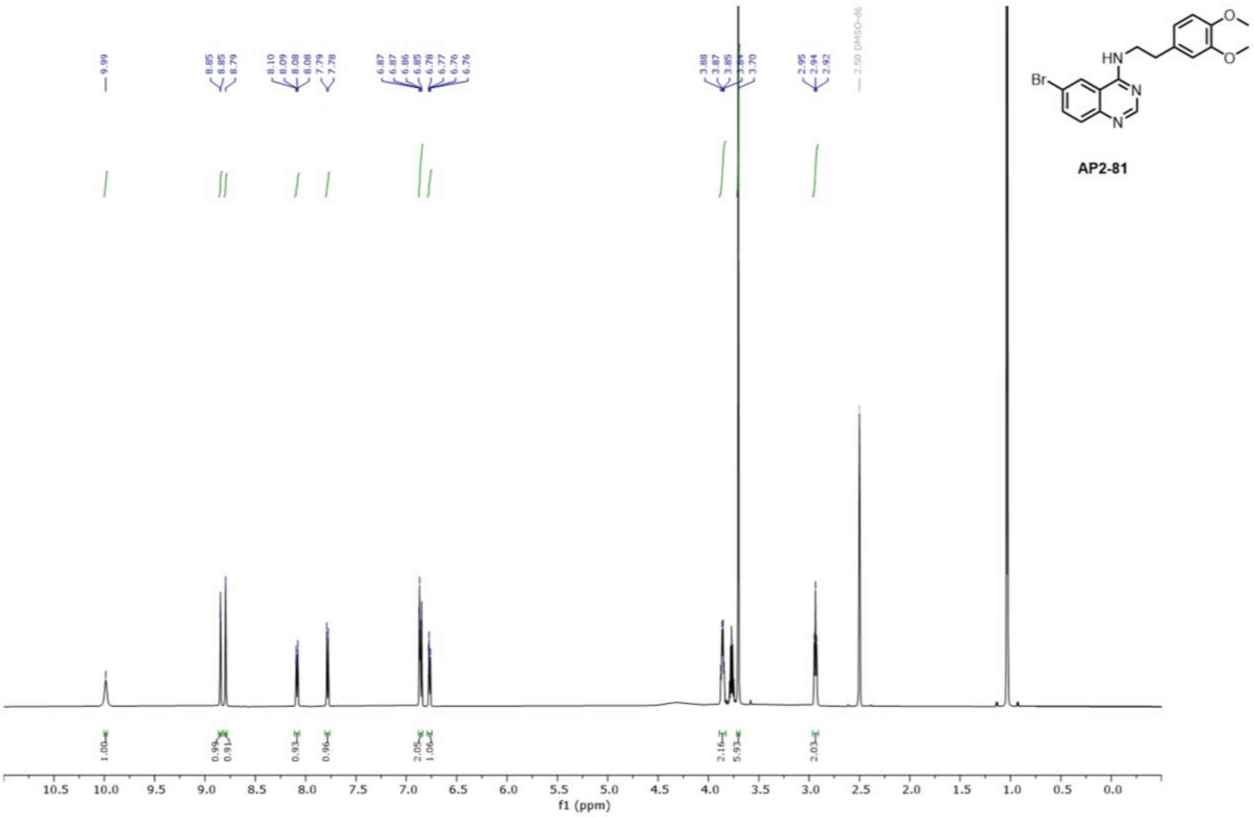

AP2-81 – ^{13}C NMR (151 MHz, DMSO-d_6)

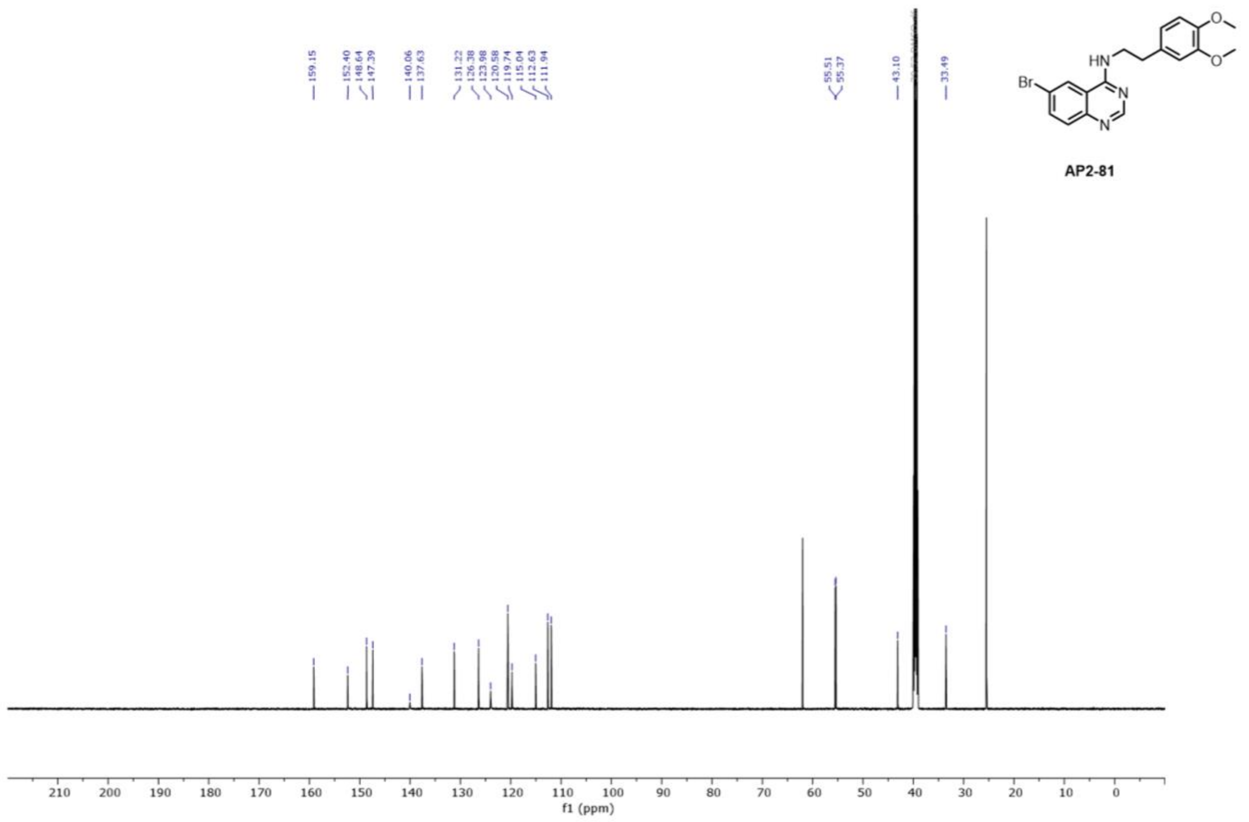

AP2-82 – ^{1}H NMR (600 MHz, DMSO-*d6*)

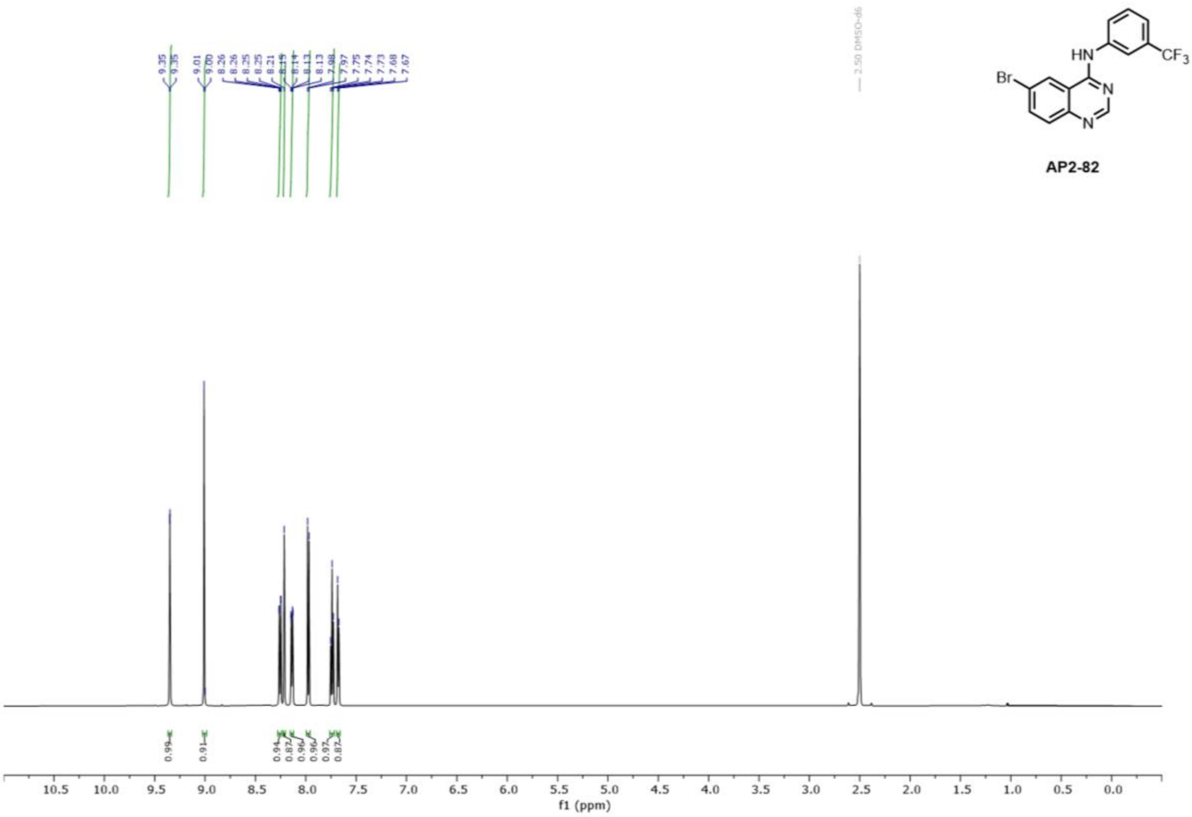

AP2-82 – ^{13}C NMR (151 MHz, DMSO-*d6*)

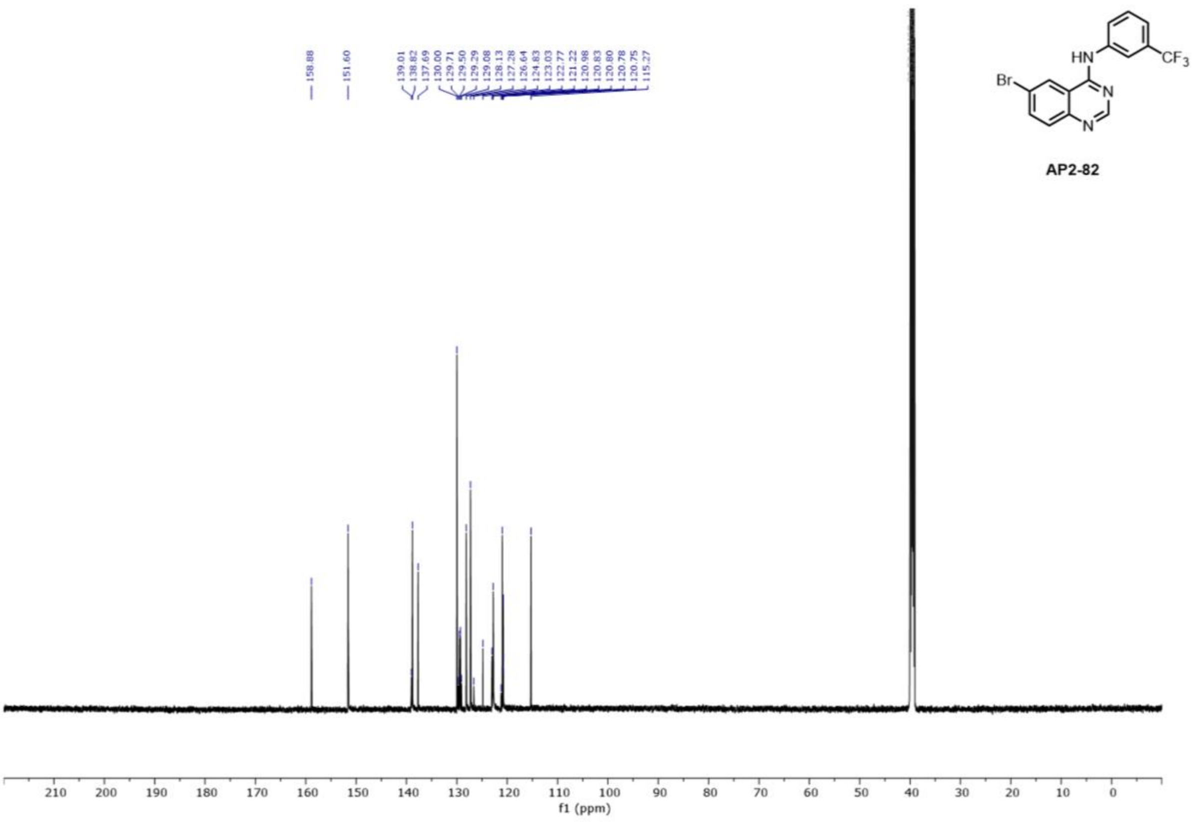

AP2-83 – ^{1}H NMR (600 MHz, DMSO-d_6)

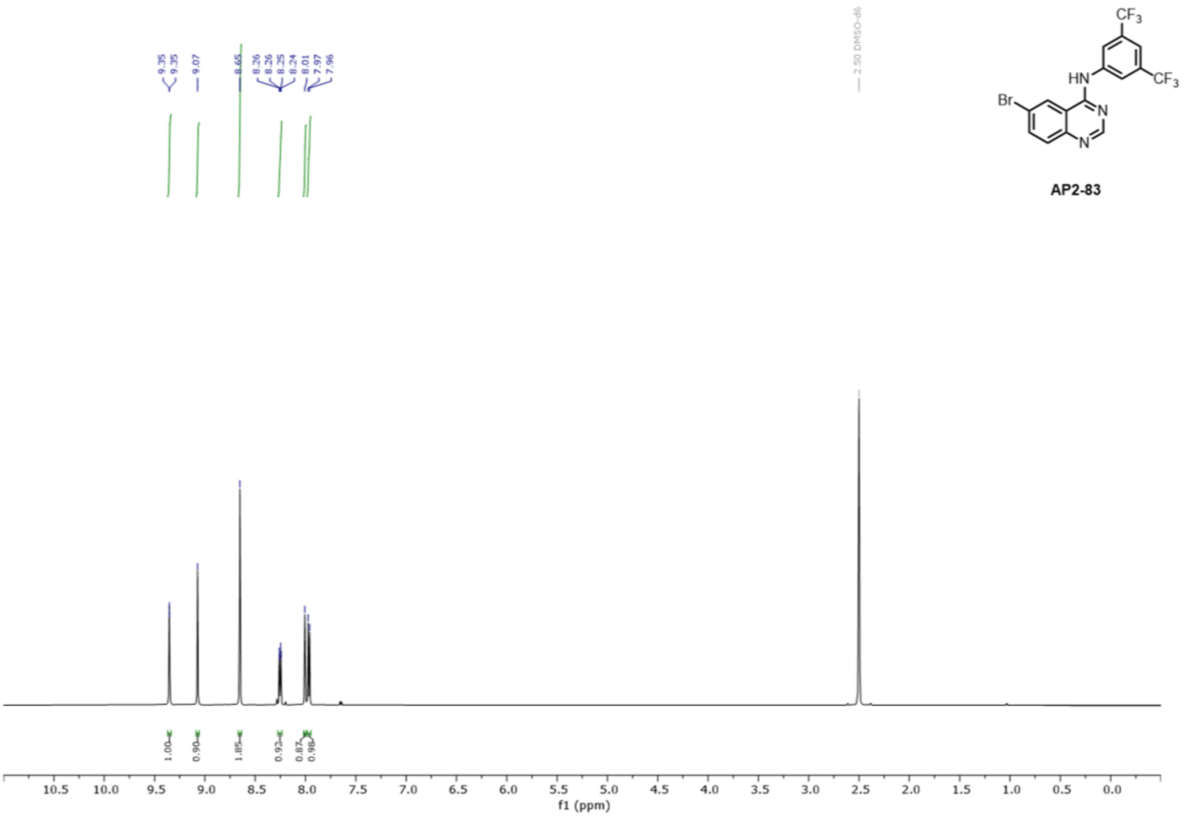

AP2-83 – ^{13}C NMR (151 MHz, DMSO-d_6)

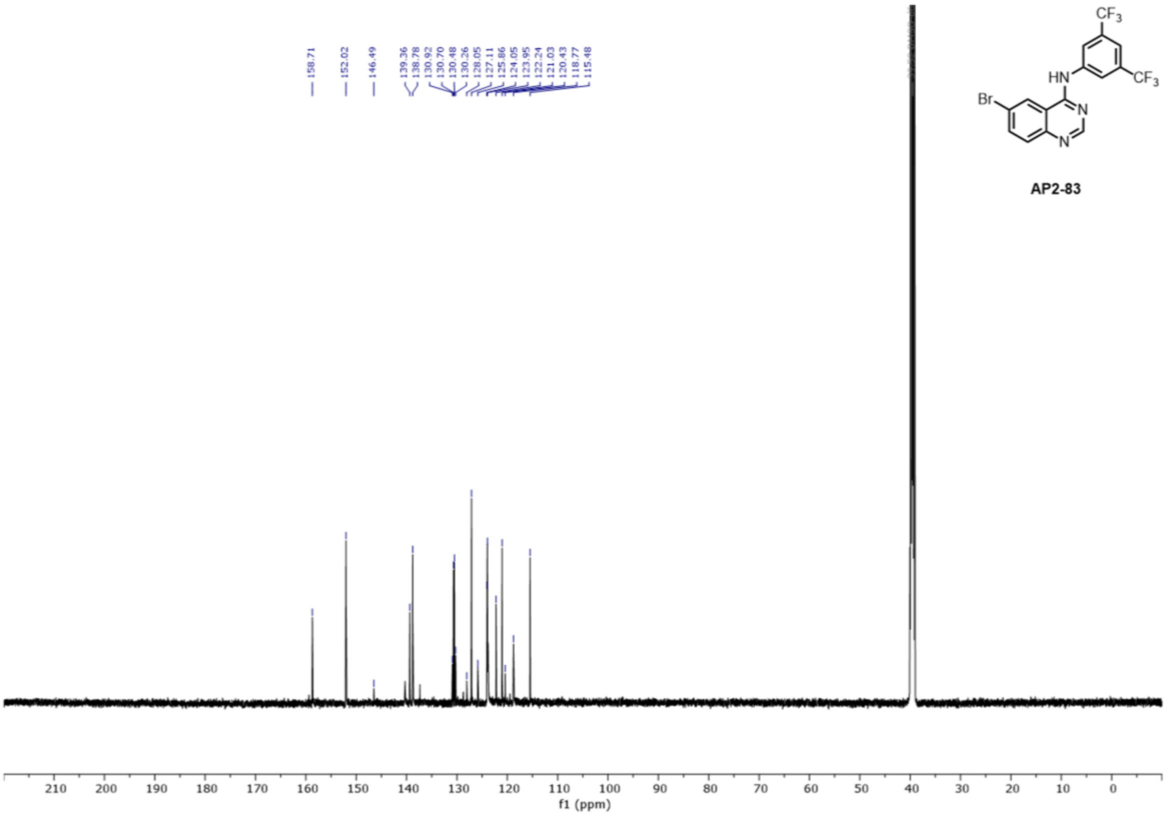

AP2-84 – ^{1}H NMR (600 MHz, DMSO-d_6)

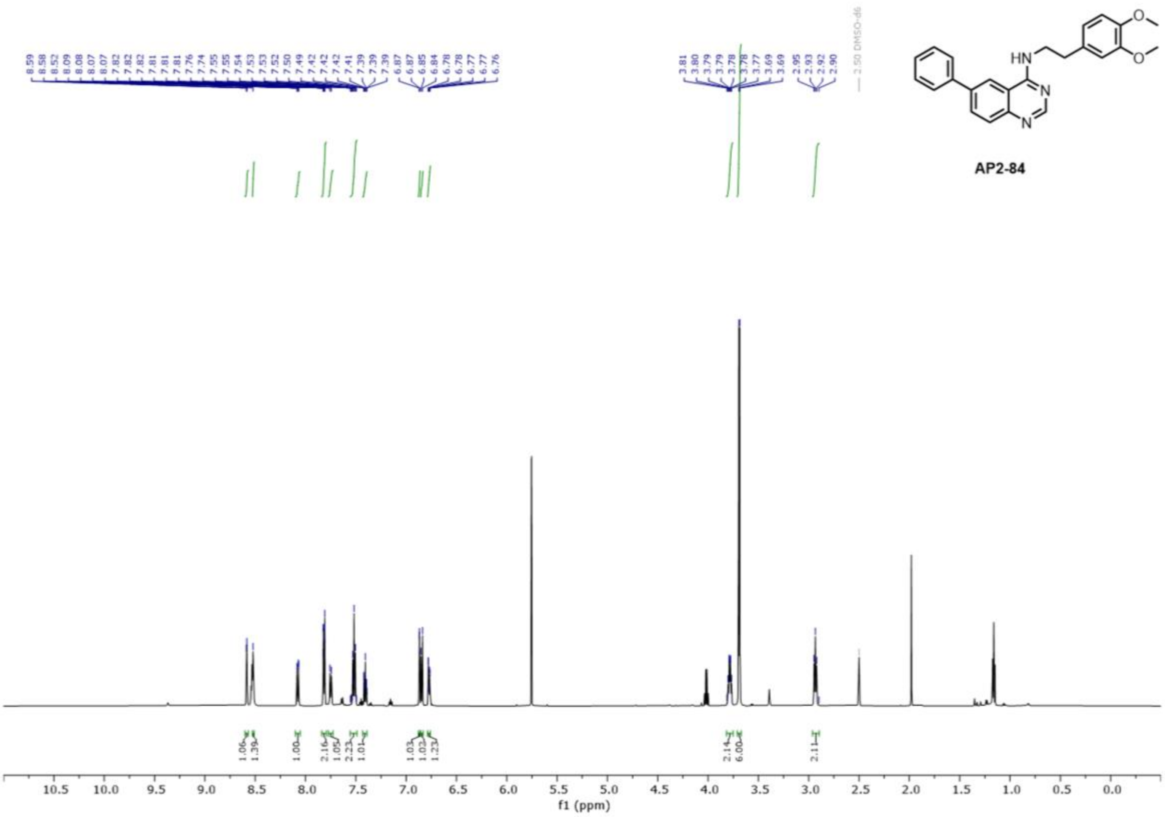

AP2-84 – ^{13}C NMR (151 MHz, DMSO-d_6)

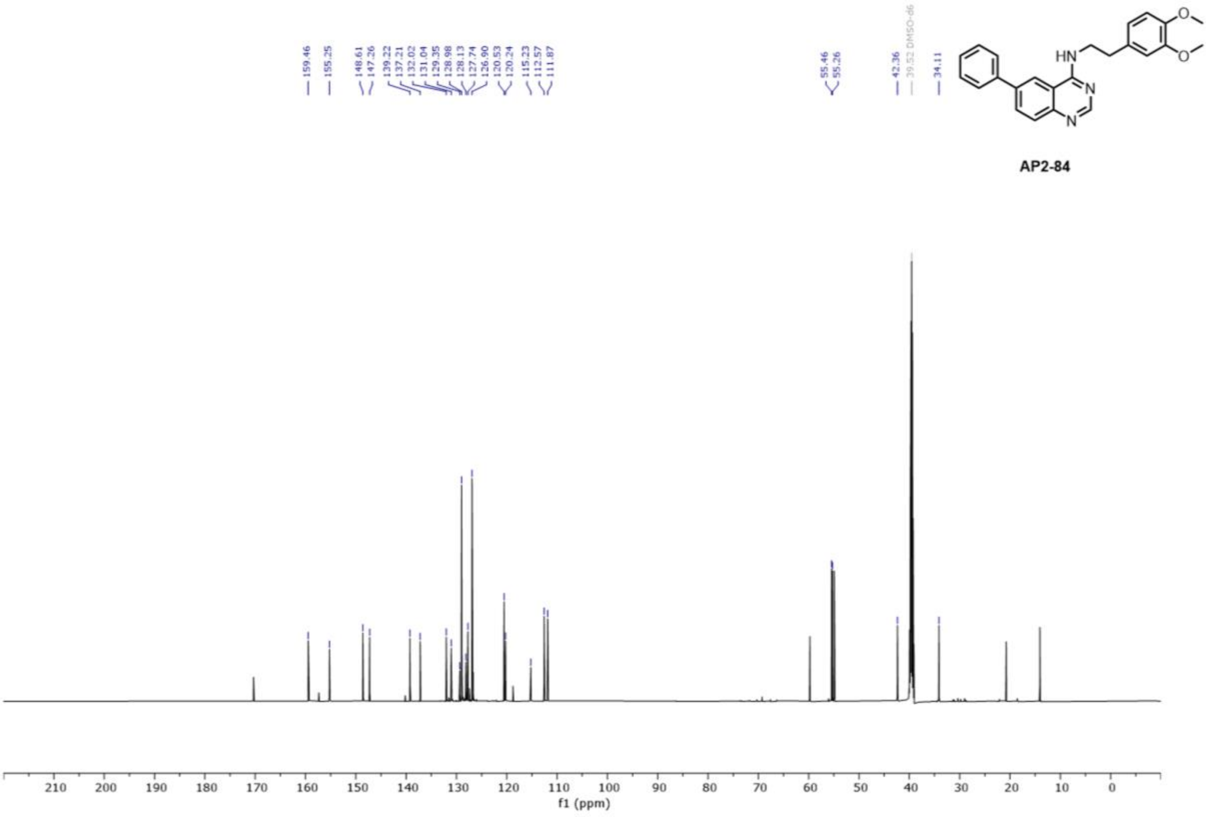

AP2-86 – ^{1}H NMR (600 MHz, DMSO-*d*$_6$)

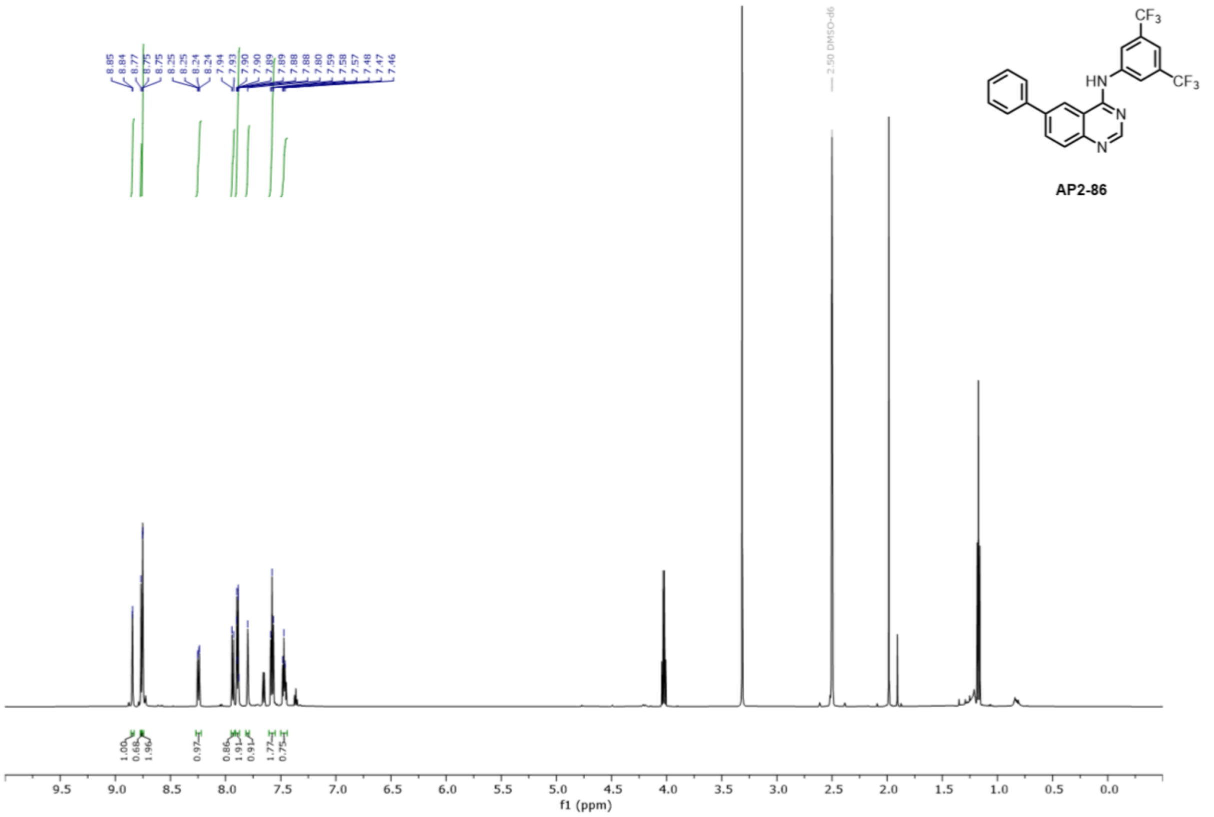

AP2-86 – ^{13}C NMR (151 MHz, DMSO-*d*$_6$)

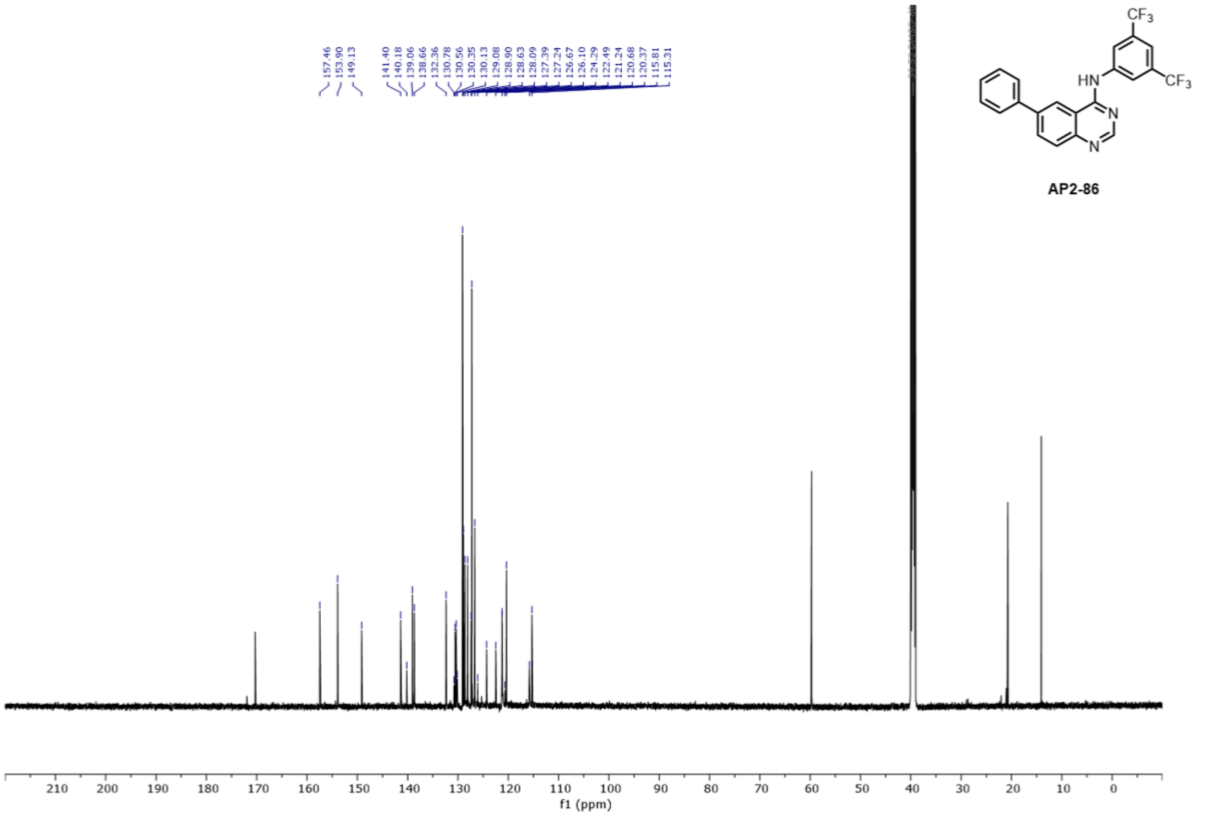

AP2-89 – ^{1}H NMR (600 MHz, DMSO-d_6)

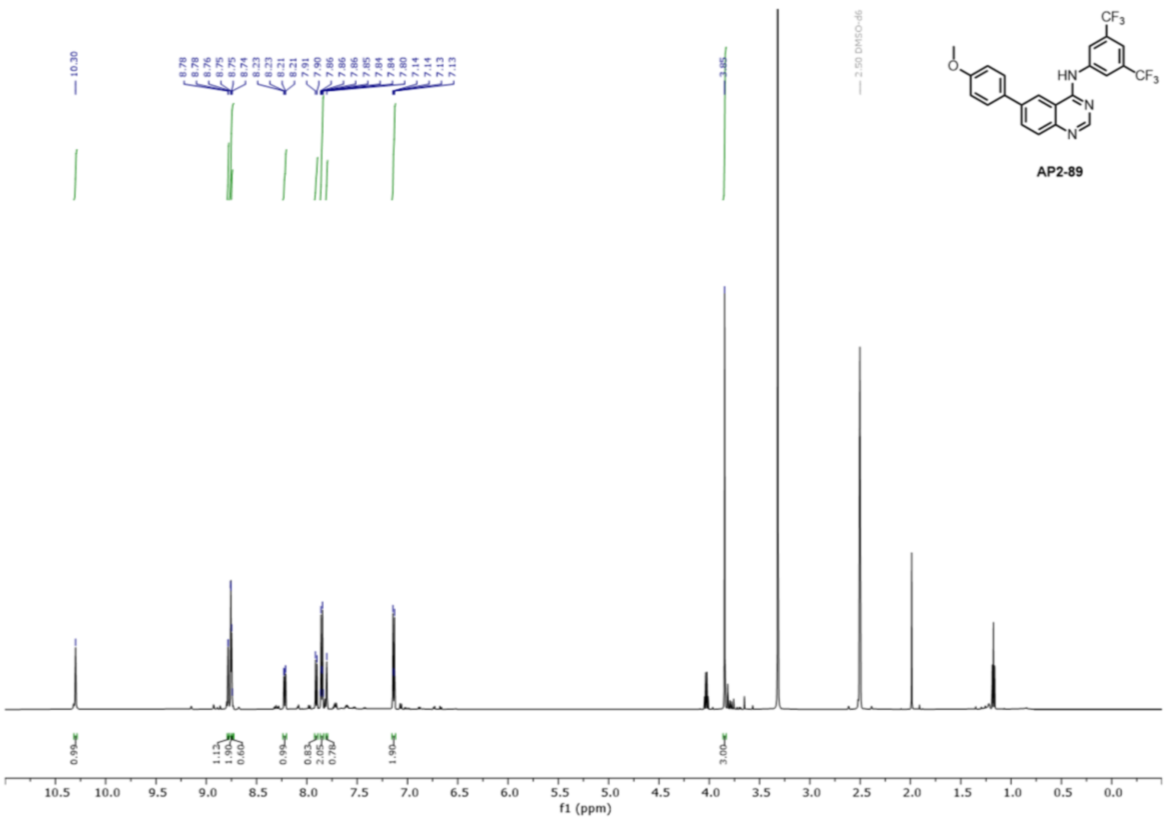

AP2-89 – ^{13}C NMR (151 MHz, DMSO-d_6)

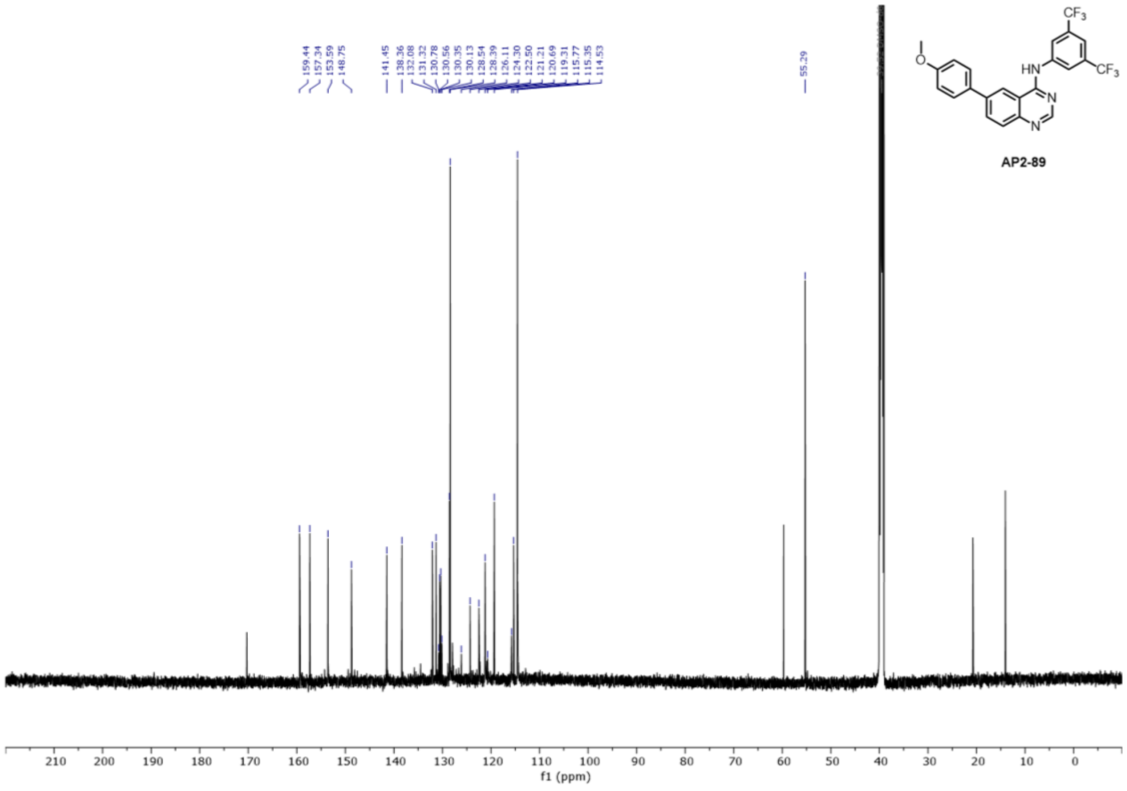

AP2-94 – ^{1}H NMR (600 MHz, DMSO-d_6)

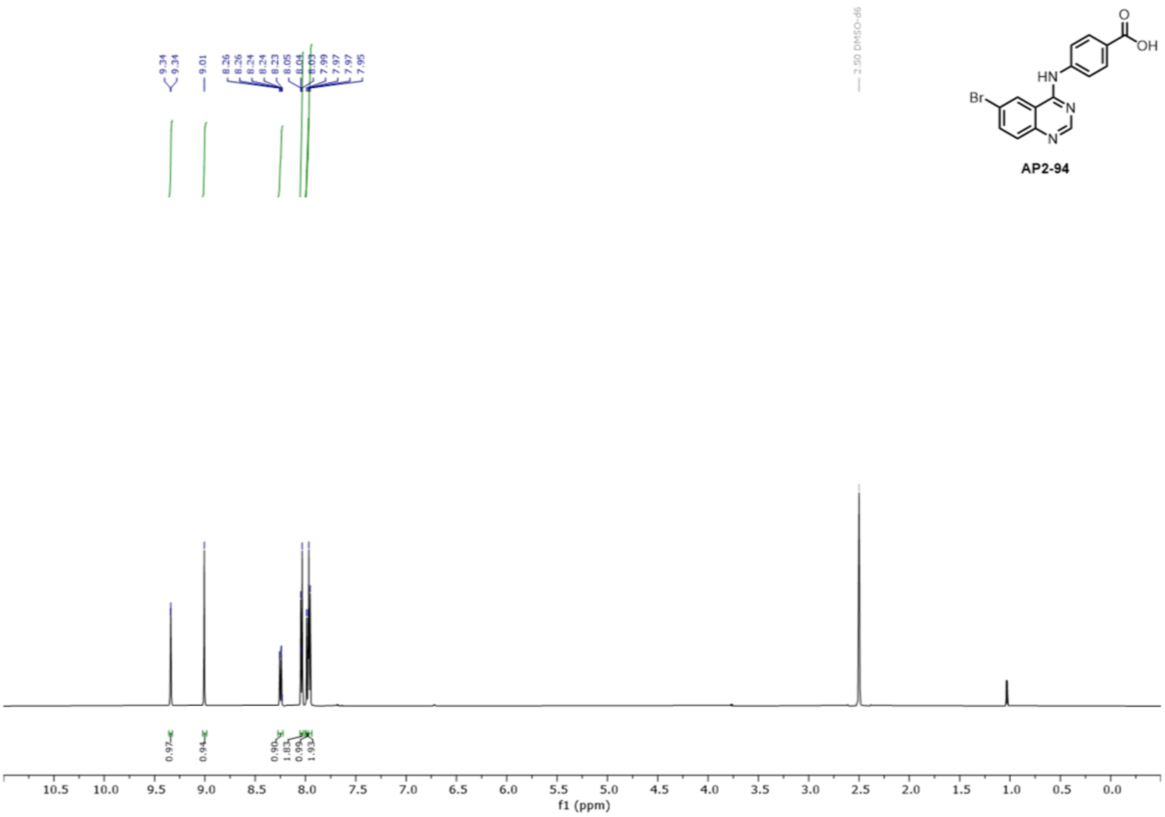

AP2-94 – ^{13}C NMR (151 MHz, DMSO-d_6)

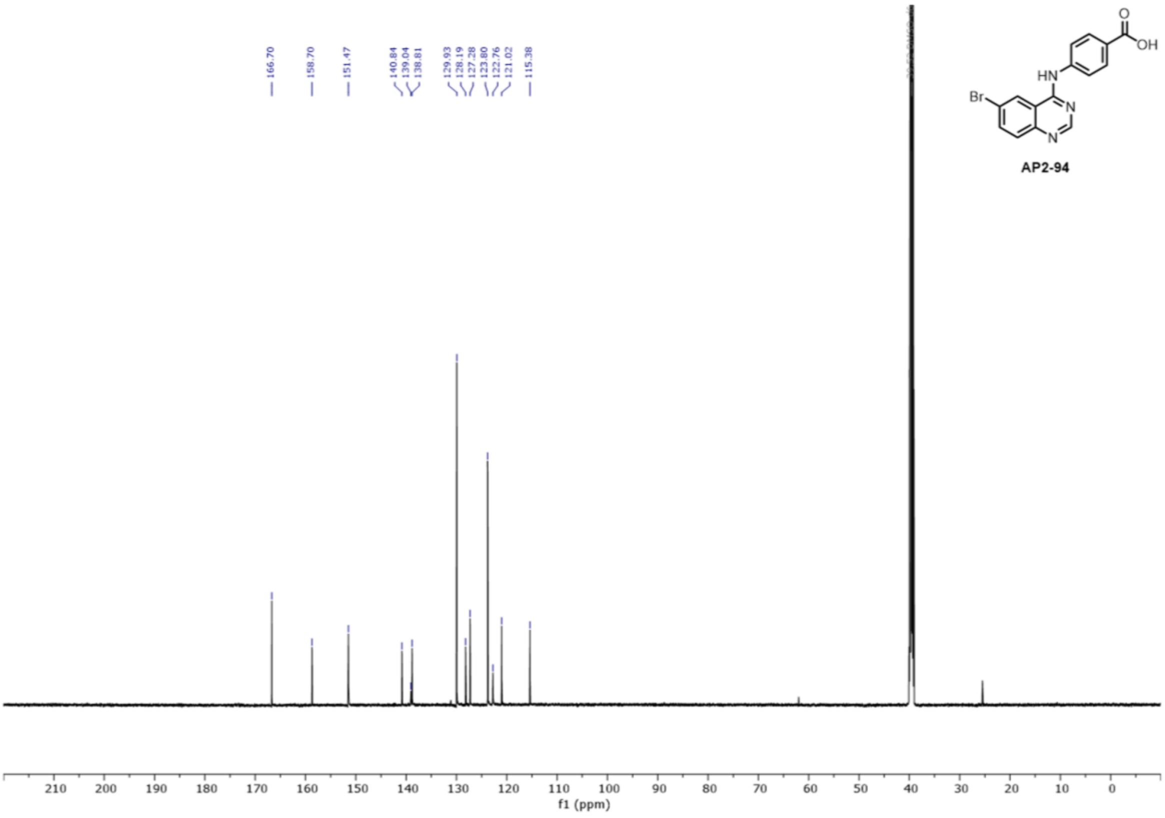

AP2-99 – ^{1}H NMR (600 MHz, DMSO-*d6*)

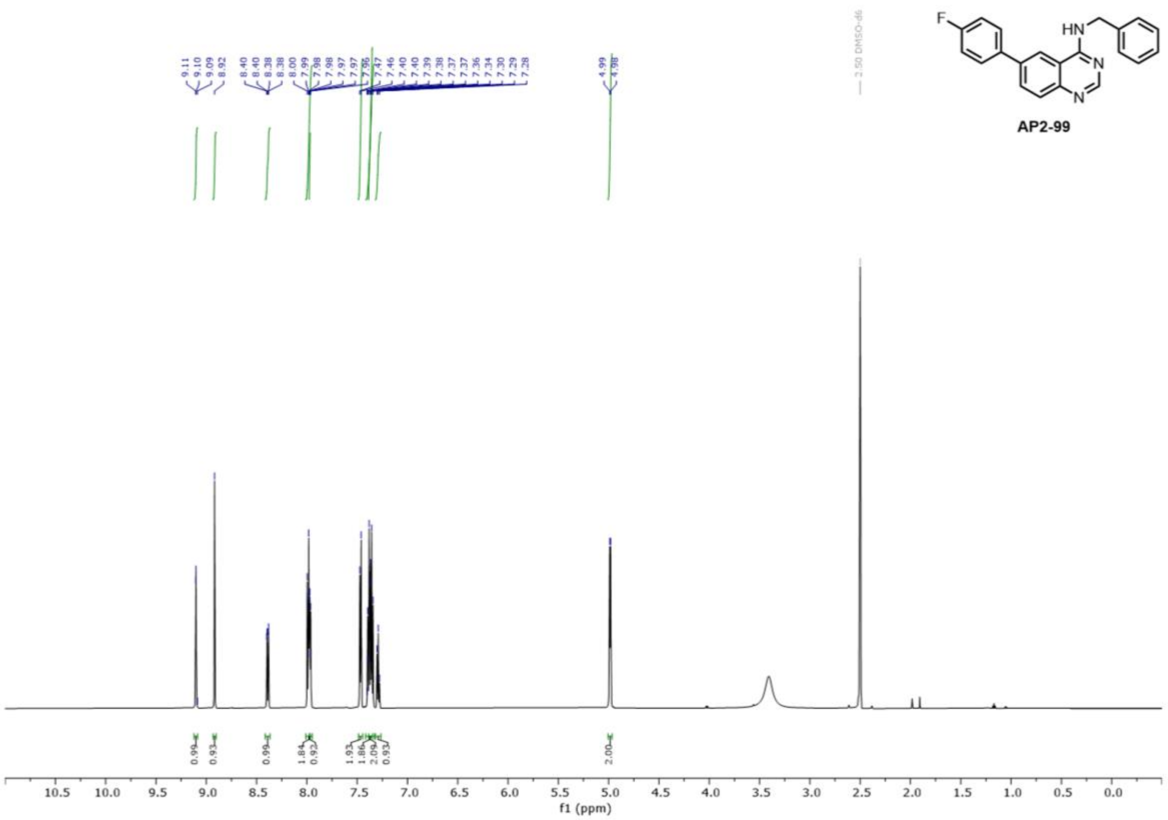

AP2-99 – ^{13}C NMR (151 MHz, DMSO-*d6*)

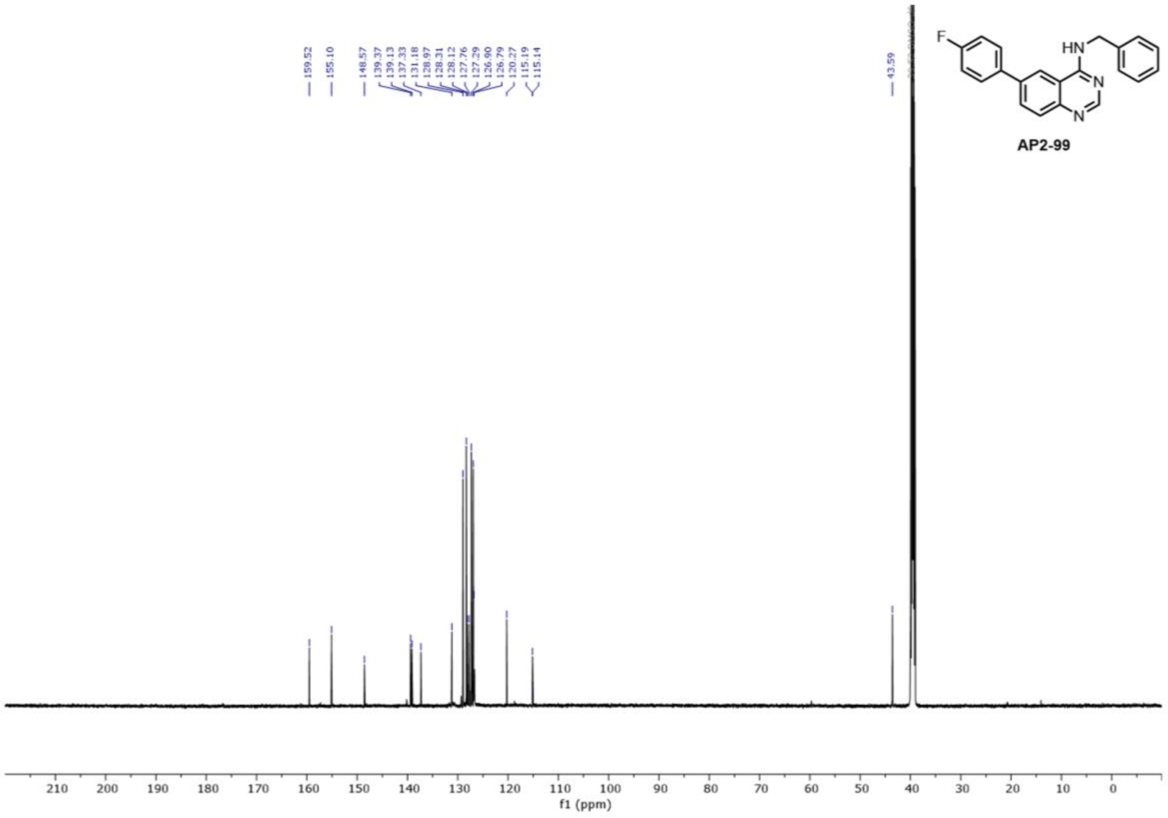

AP2-100 – ^{1}H NMR (600 MHz, DMSO-*d6*)

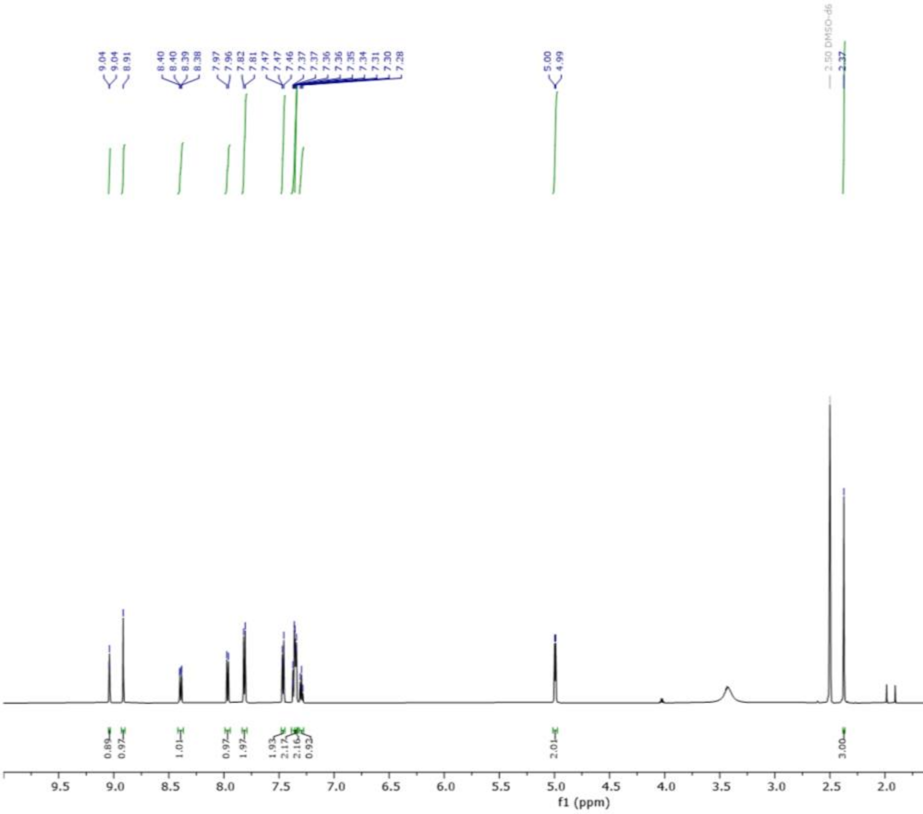

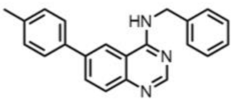

AP2-100

AP2-100 – ^{13}C NMR (151 MHz, DMSO-*d6*)

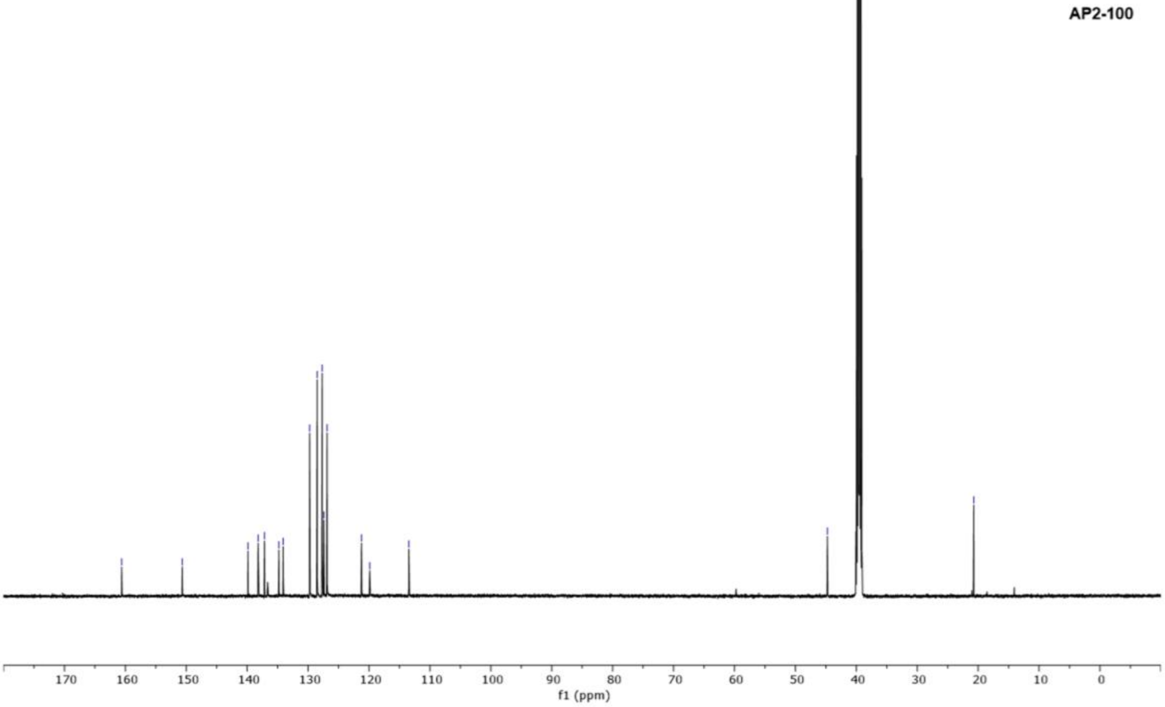

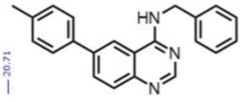

AP2-100

AP2-101 – ^{1}H NMR (600 MHz, DMSO-d_6)

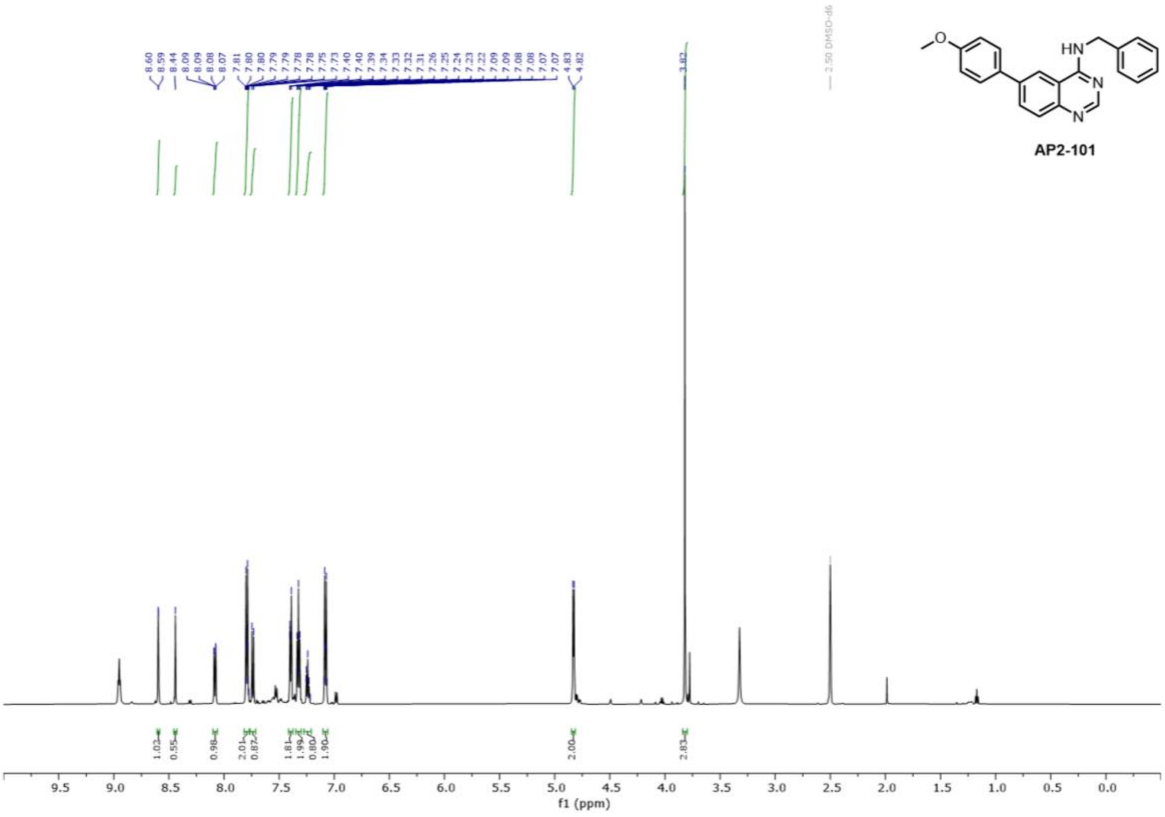

AP2-101 – ^{13}C NMR (151 MHz, DMSO-d_6)

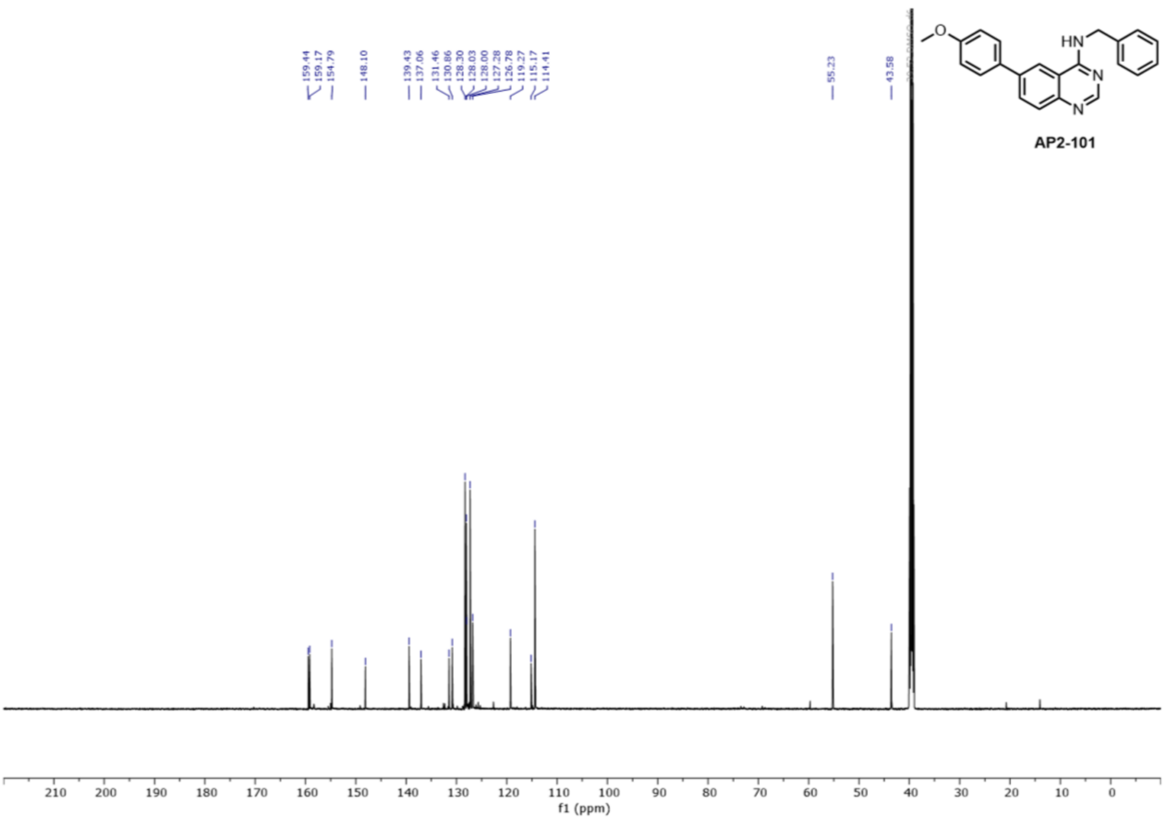

AP3-11 – ^{1}H NMR (600 MHz, DMSO-d_6)

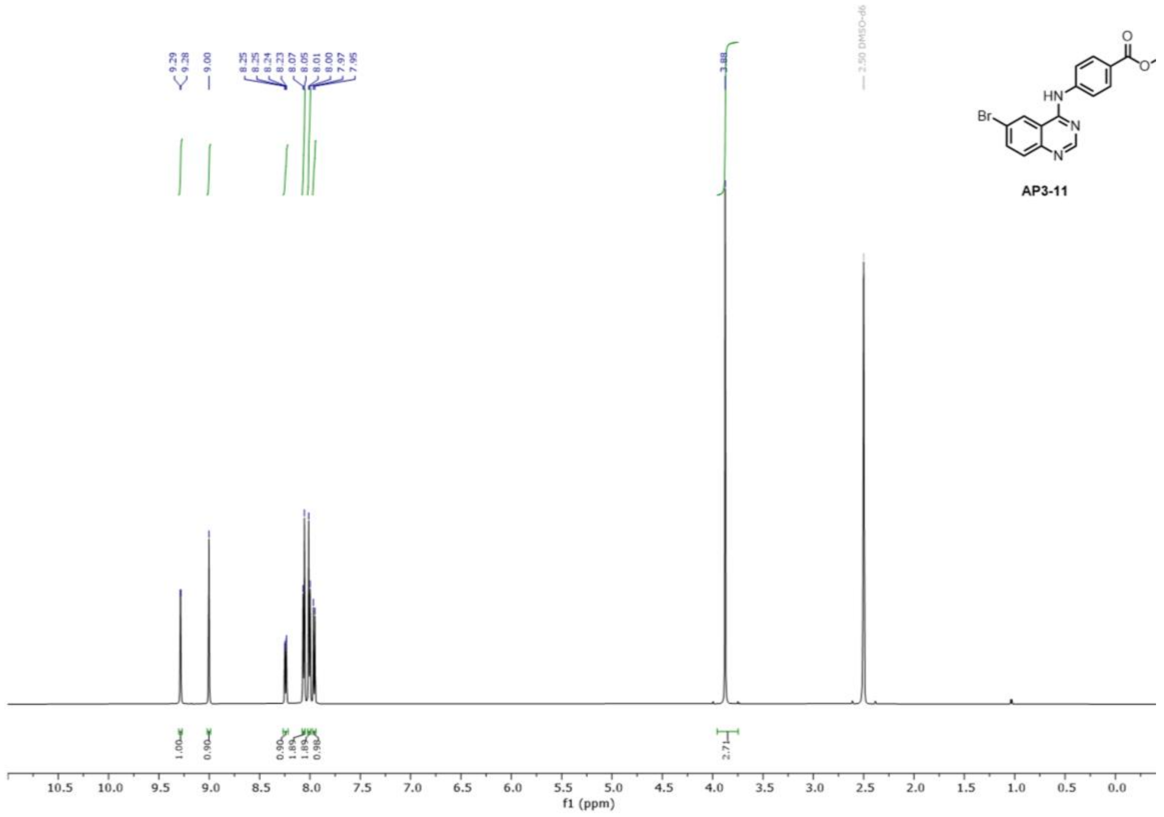

AP3-11 – ^{13}C NMR (151 MHz, DMSO-d_6)

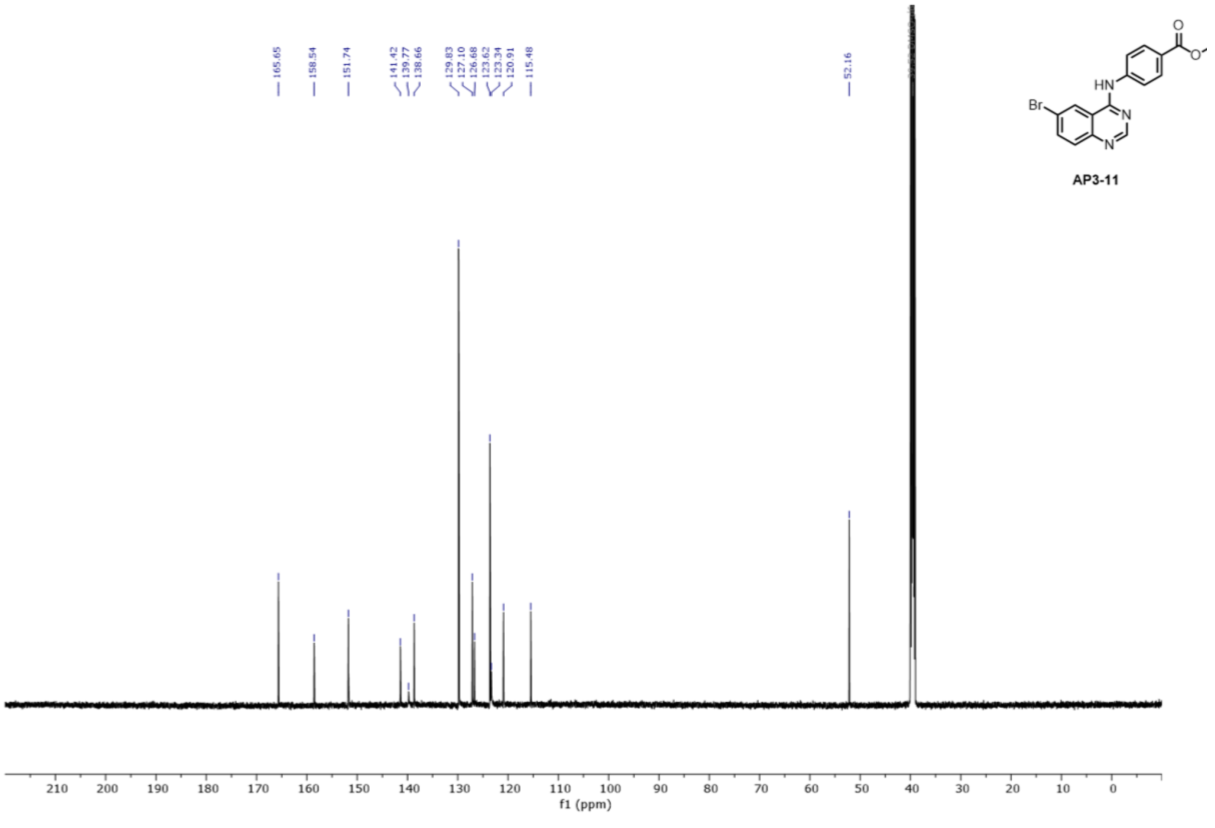

AP3-12 – ^{1}H NMR (600 MHz, DMSO-d_6)

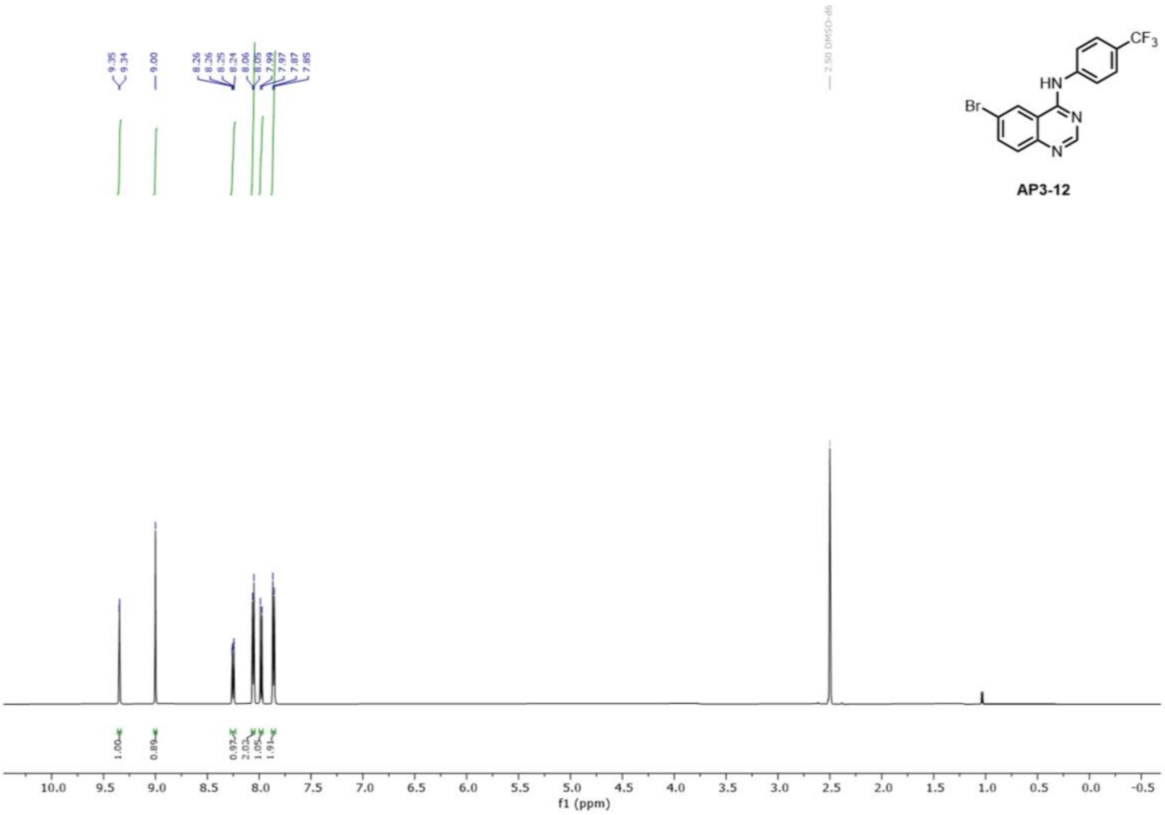

AP3-12– ^{13}C NMR (151 MHz, DMSO-d_6)

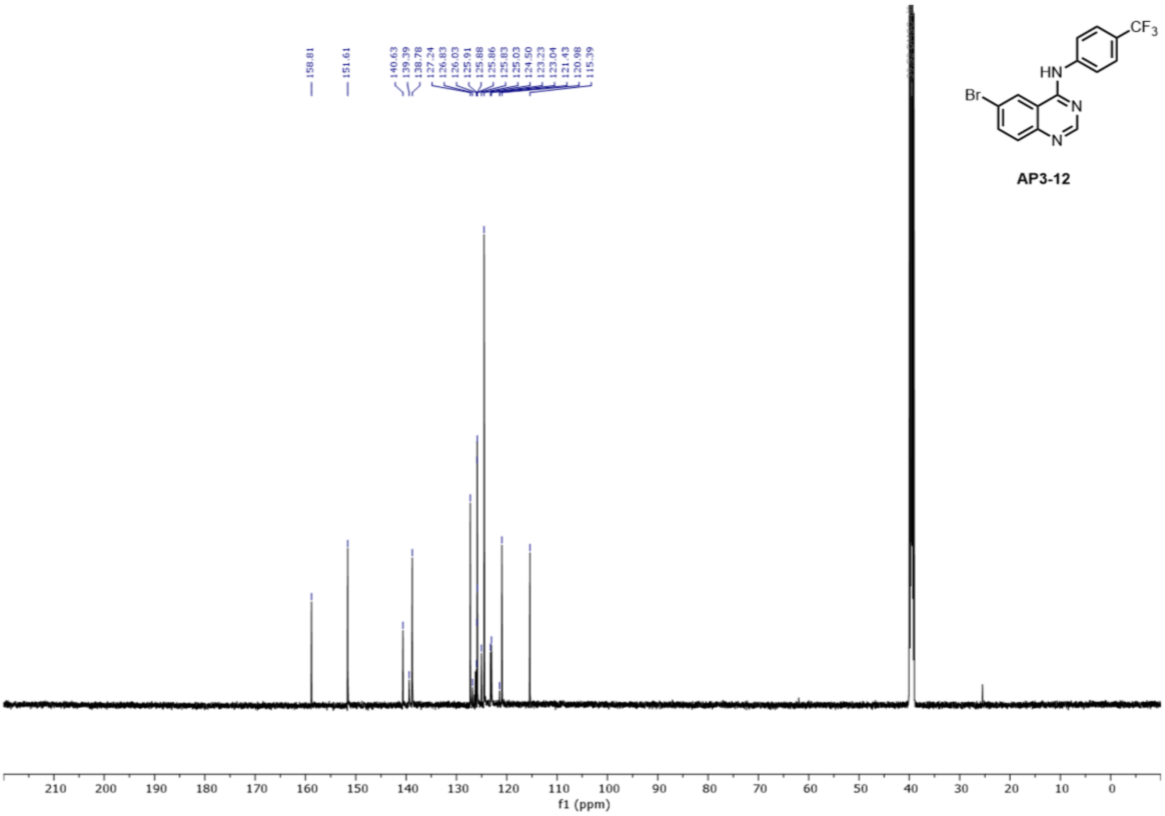

AP4-2 – ^{1}H NMR (600 MHz, DMSO-d_6)

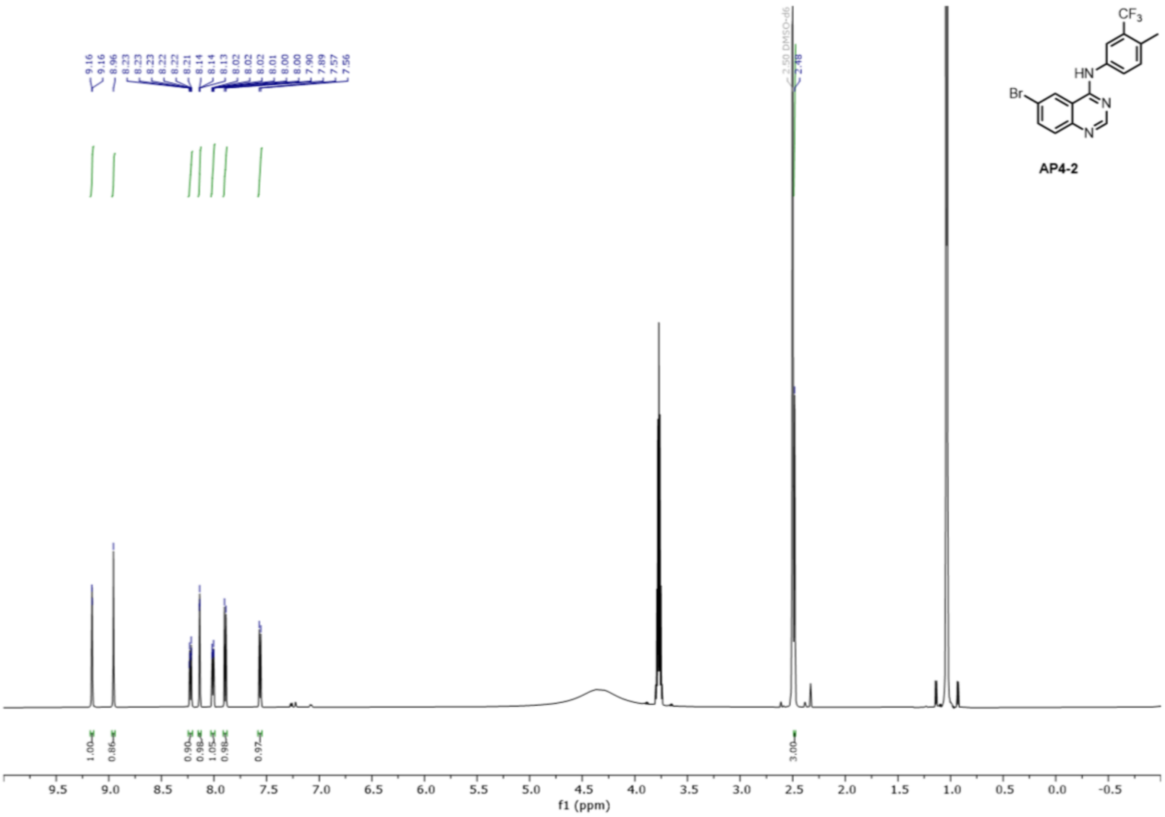

AP4-2 – ^{13}C NMR (151 MHz, DMSO-d_6)

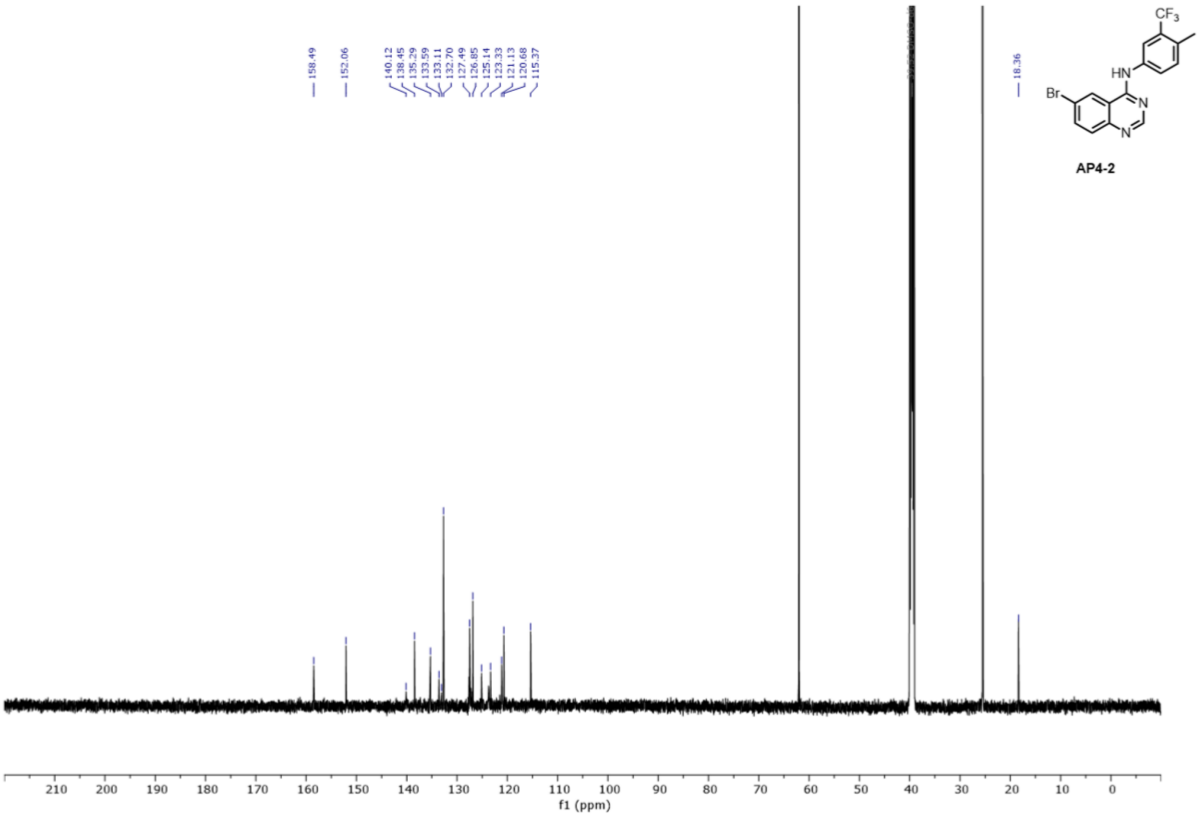

AP4-3 – ^{1}H NMR (600 MHz, DMSO-d_6)

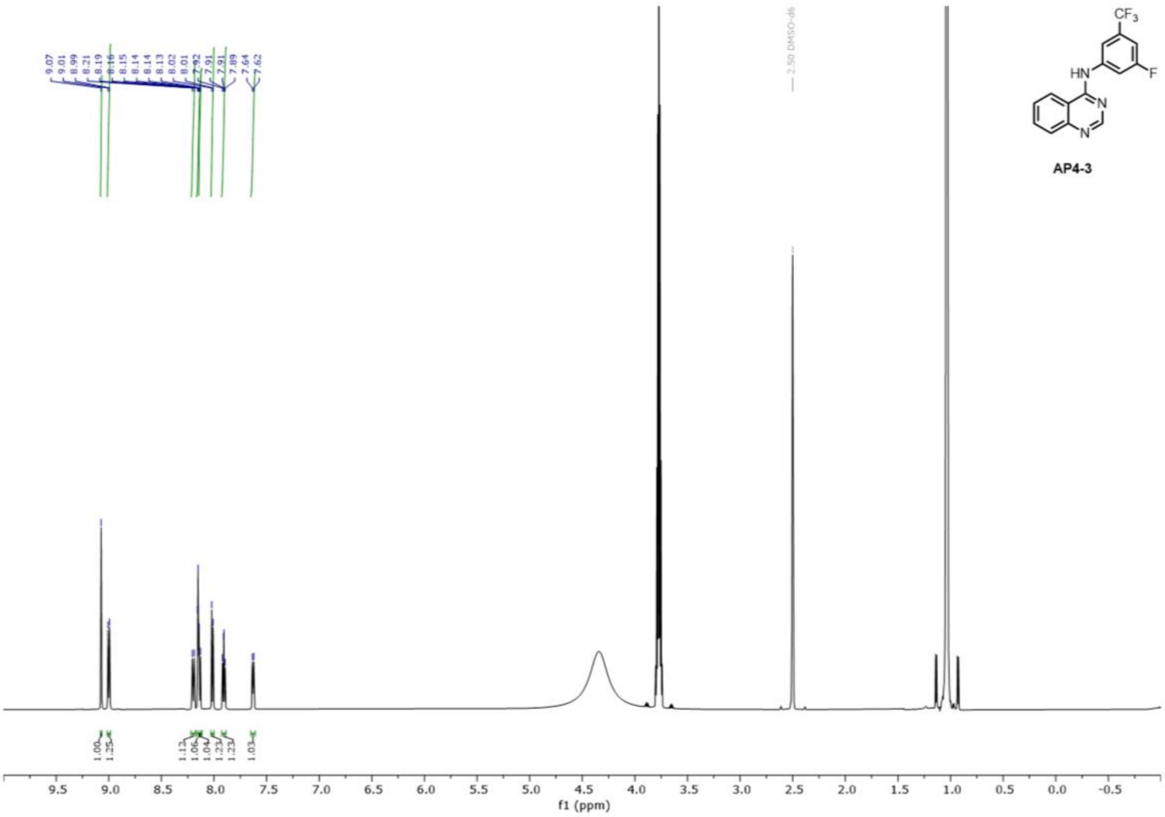

AP4-3 – ^{13}C NMR (151 MHz, DMSO-d_6)

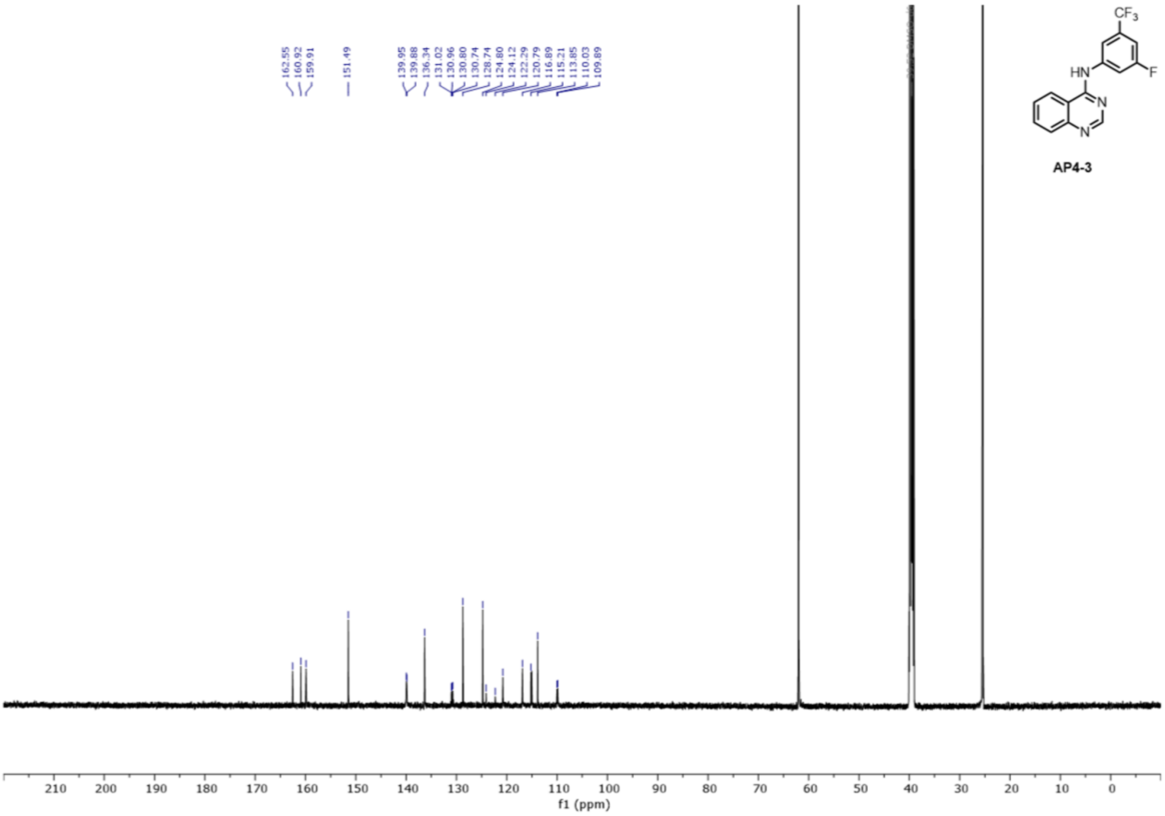

AP4-5 – ^{1}H NMR (600 MHz, DMSO-*d*$_6$)

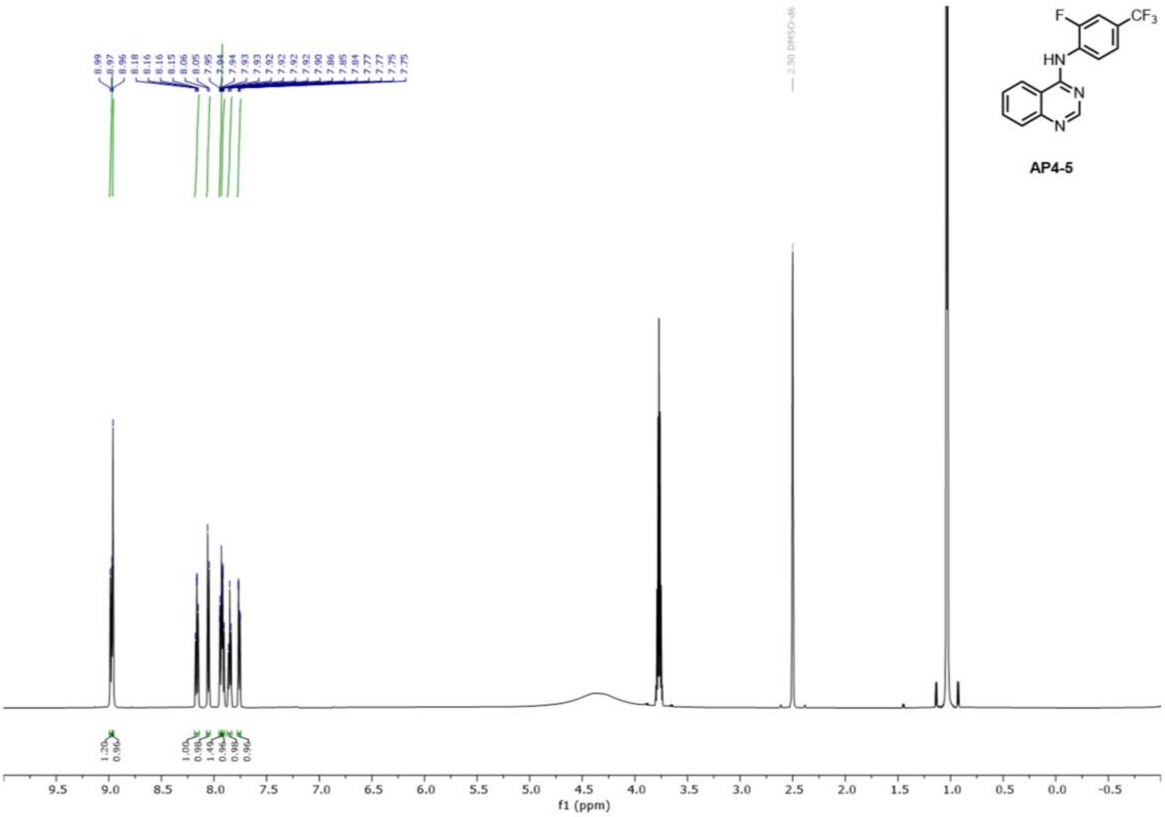

AP4-5 – ^{13}C NMR (151 MHz, DMSO-*d*$_6$)

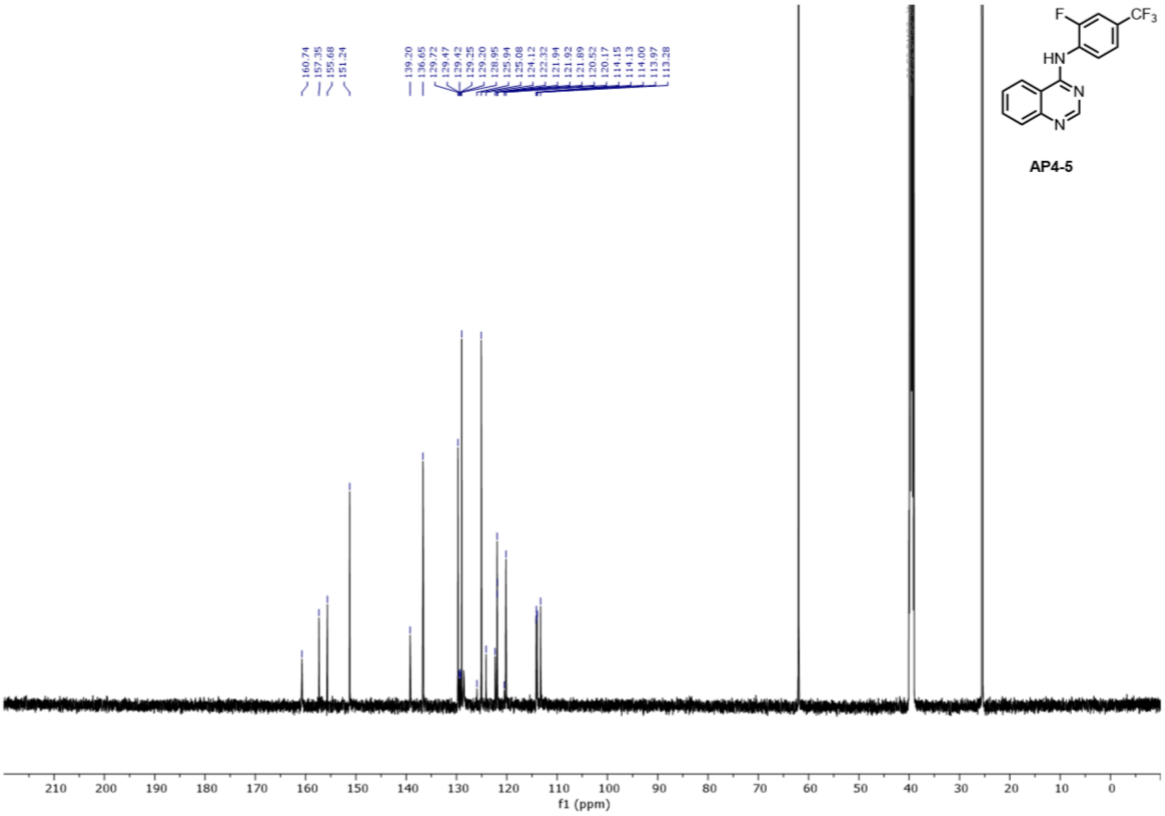

AP4-6 – ^{1}H NMR (600 MHz, DMSO-d_6)

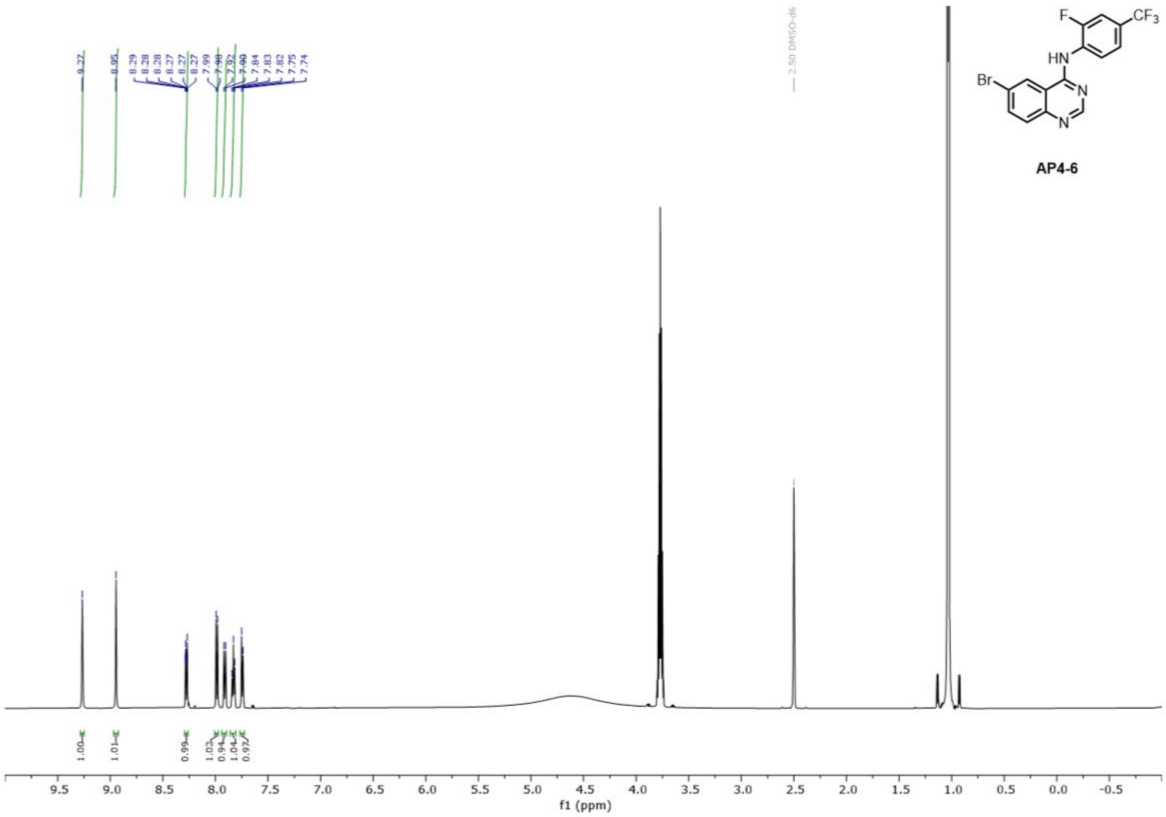

AP4-6 – ^{13}C NMR (151 MHz, DMSO-d_6)

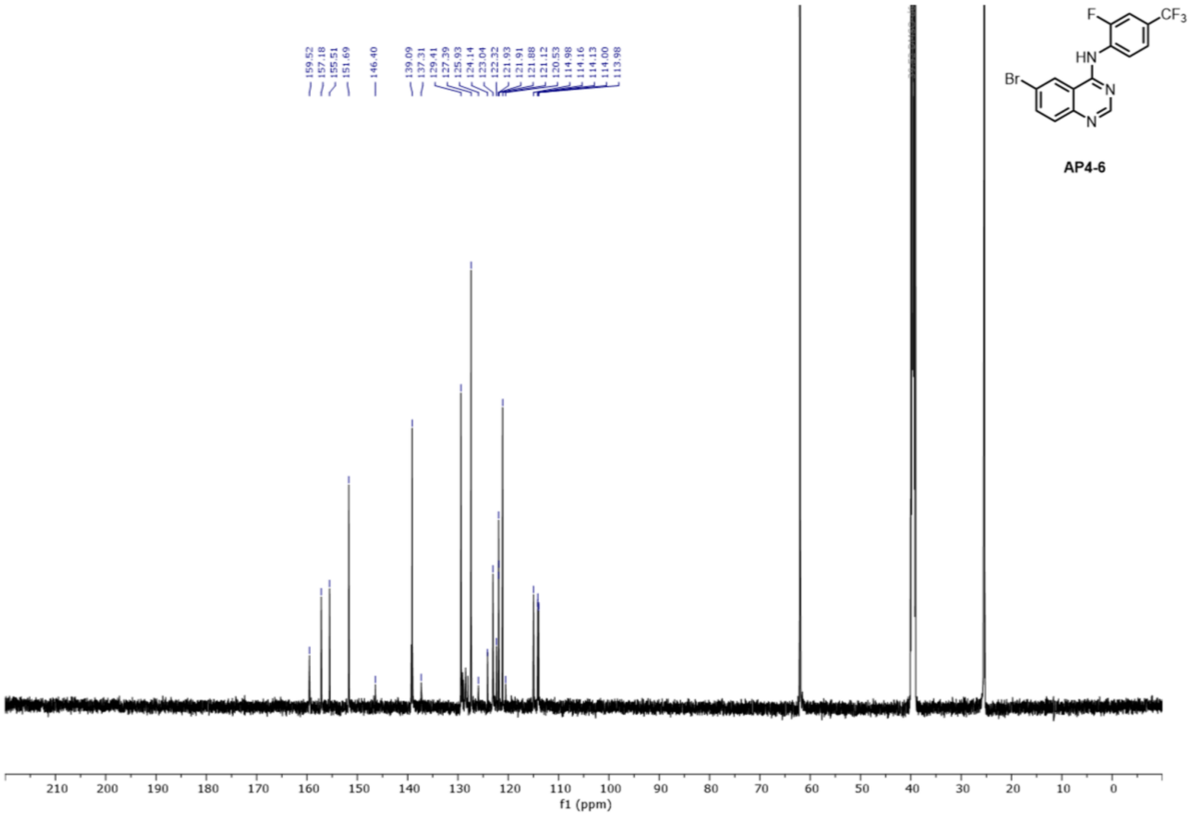

AP4-41 – ^{1}H NMR (600 MHz, DMSO-d_6)

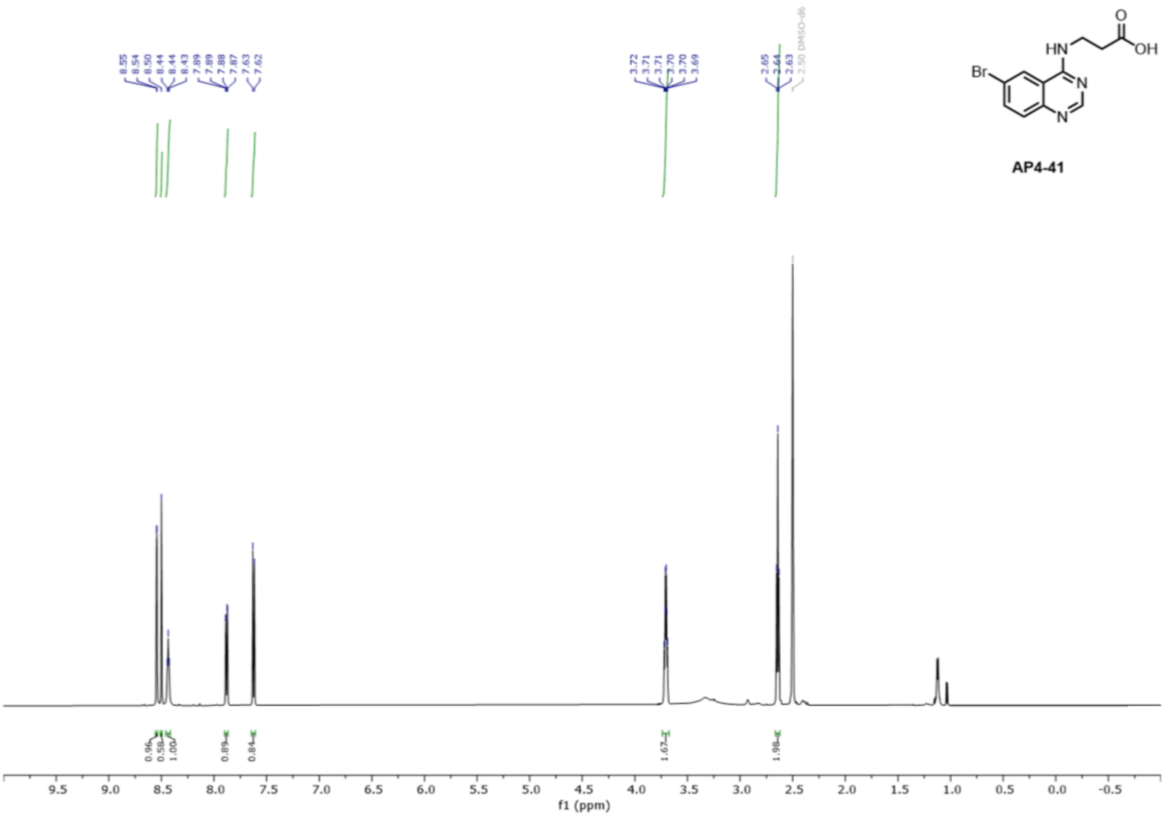

AP4-41 – ^{13}C NMR (151 MHz, DMSO-d_6)

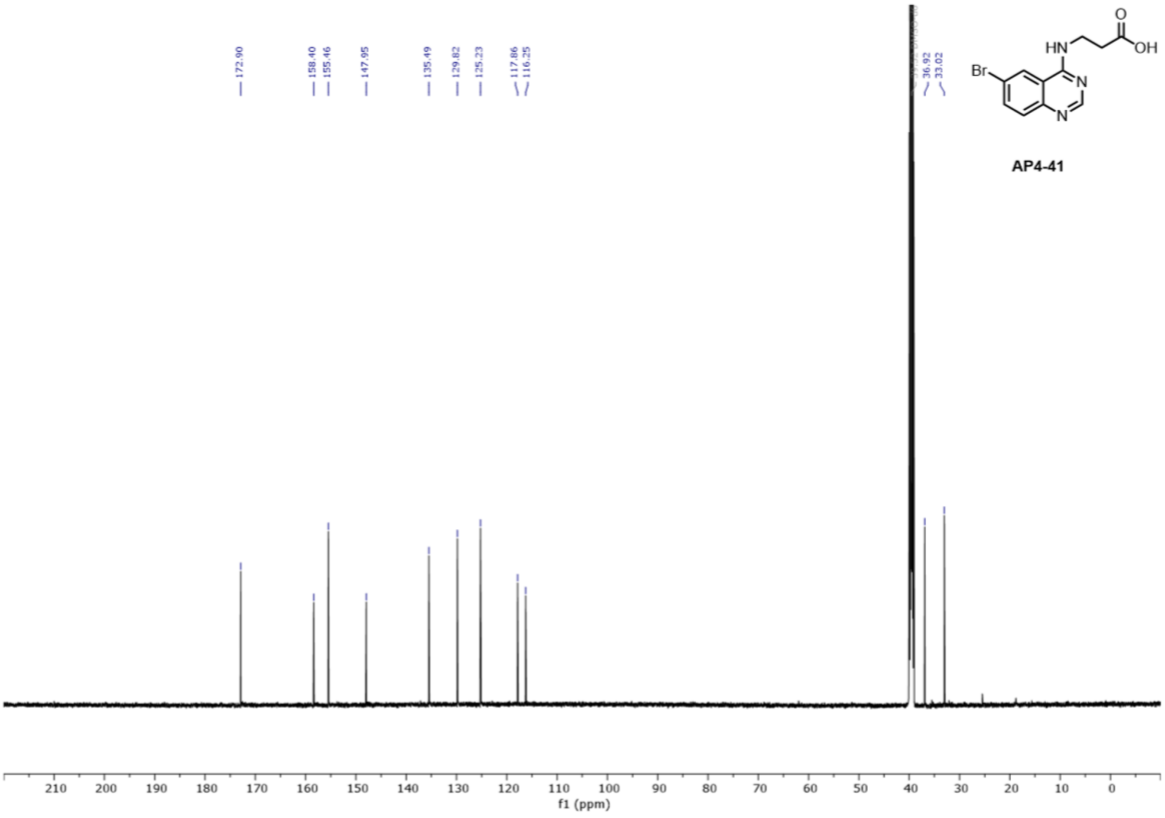

AP4-42 – ^{1}H NMR (600 MHz, DMSO-*d6*)

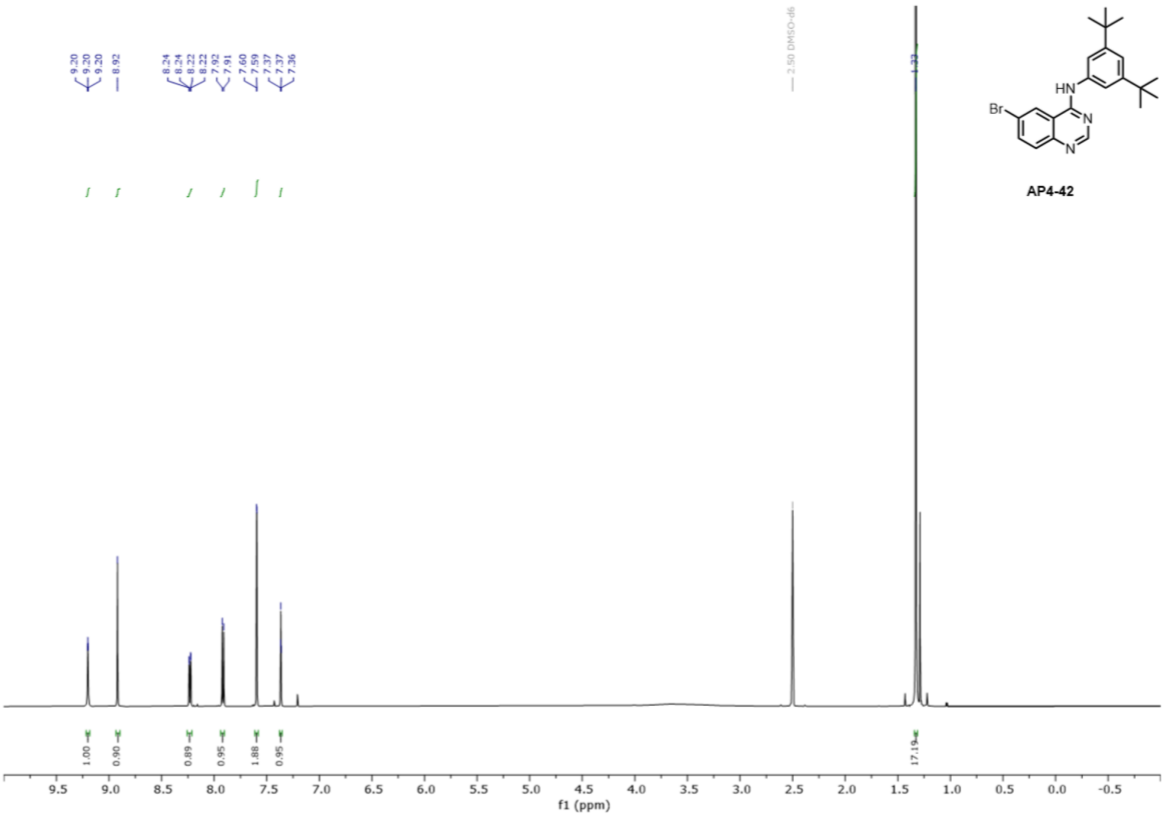

AP4-42 – ^{13}C NMR (151 MHz, DMSO-*d6*)

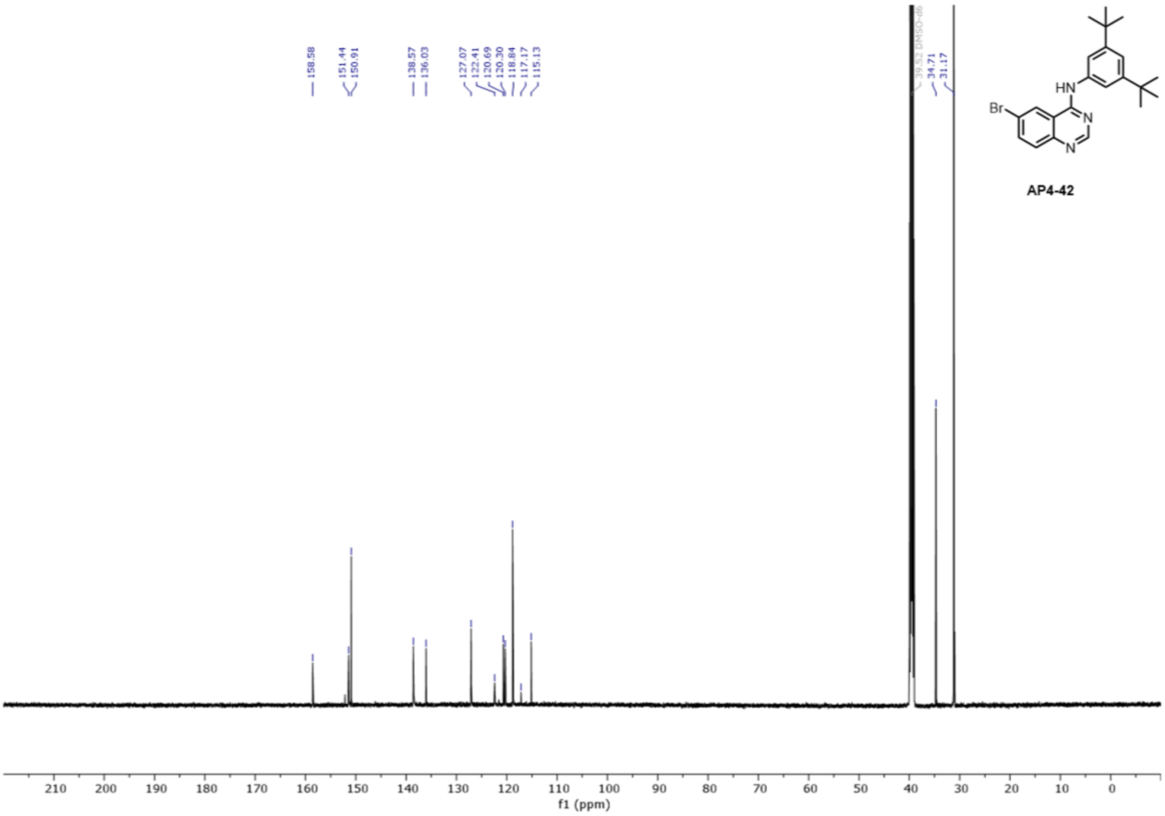

AP4-43 – ^{1}H NMR (600 MHz, DMSO-*d6*)

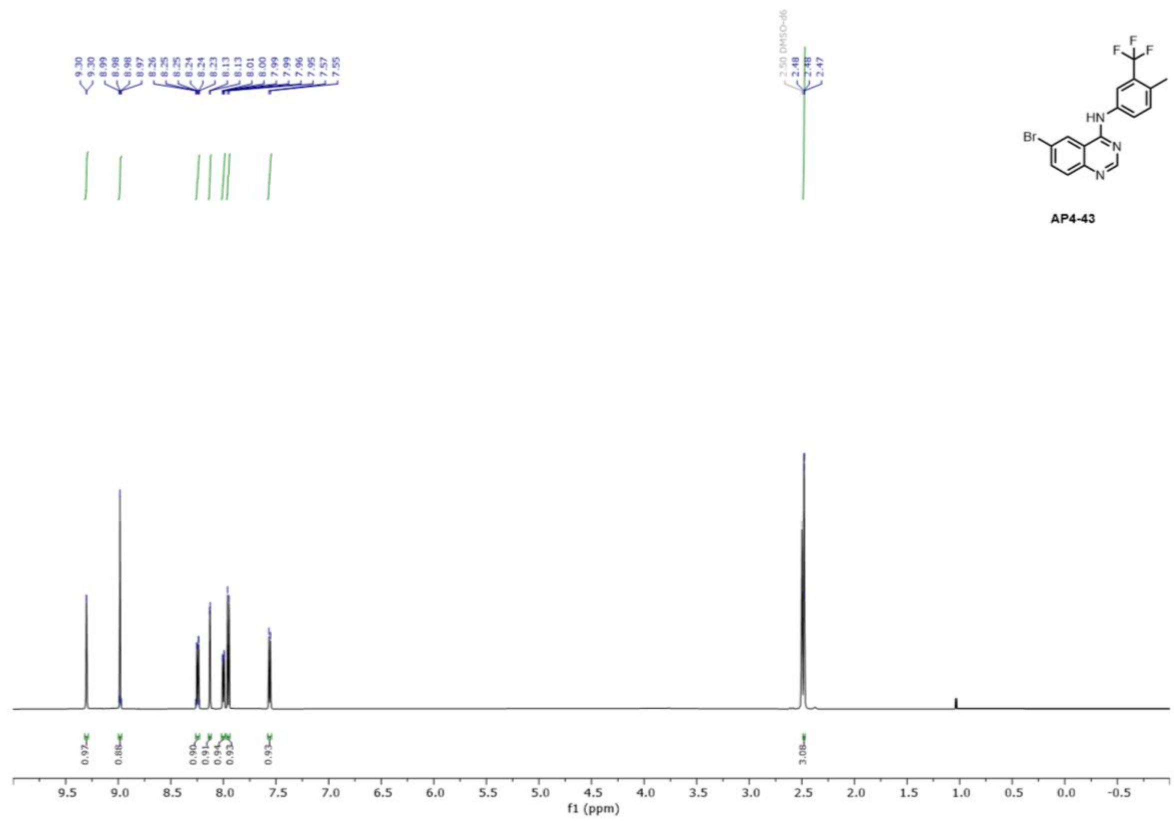

AP4-43 – ^{13}C NMR (151 MHz, DMSO-*d6*)

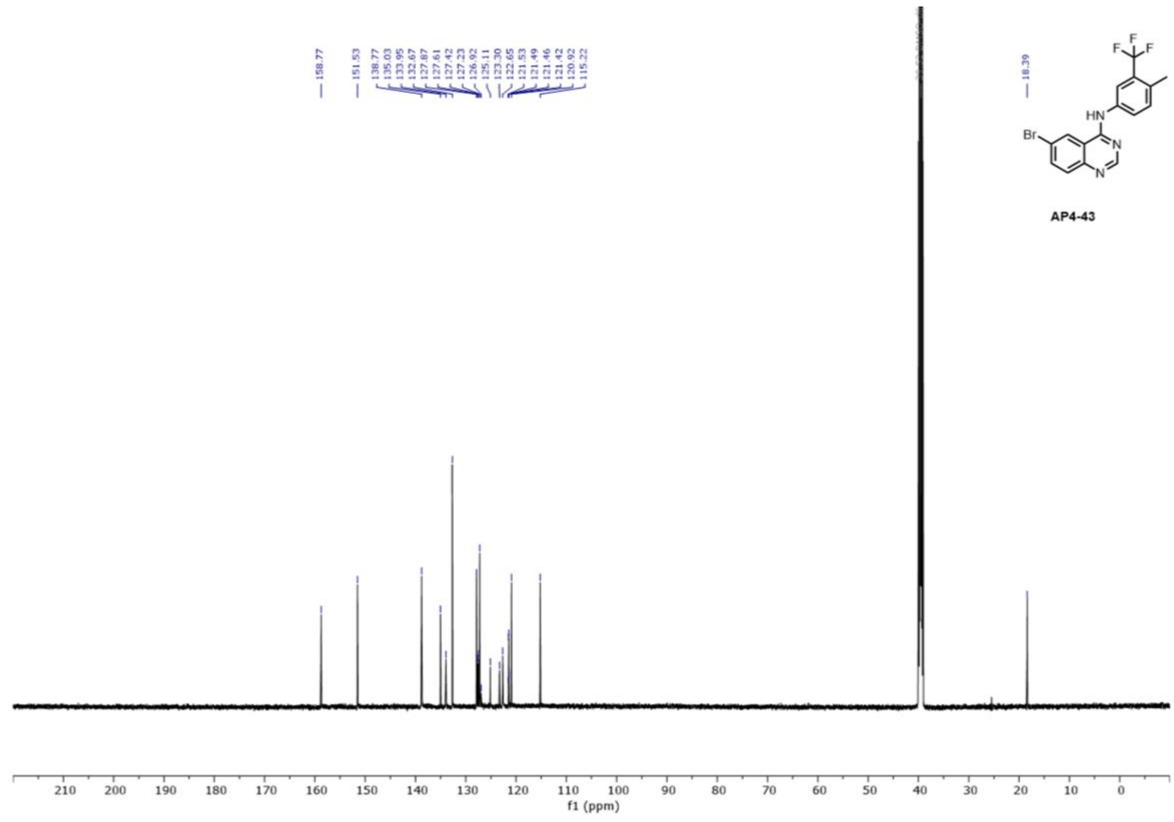

AP4-44 – ^{1}H NMR (600 MHz, DMSO-*d6*)

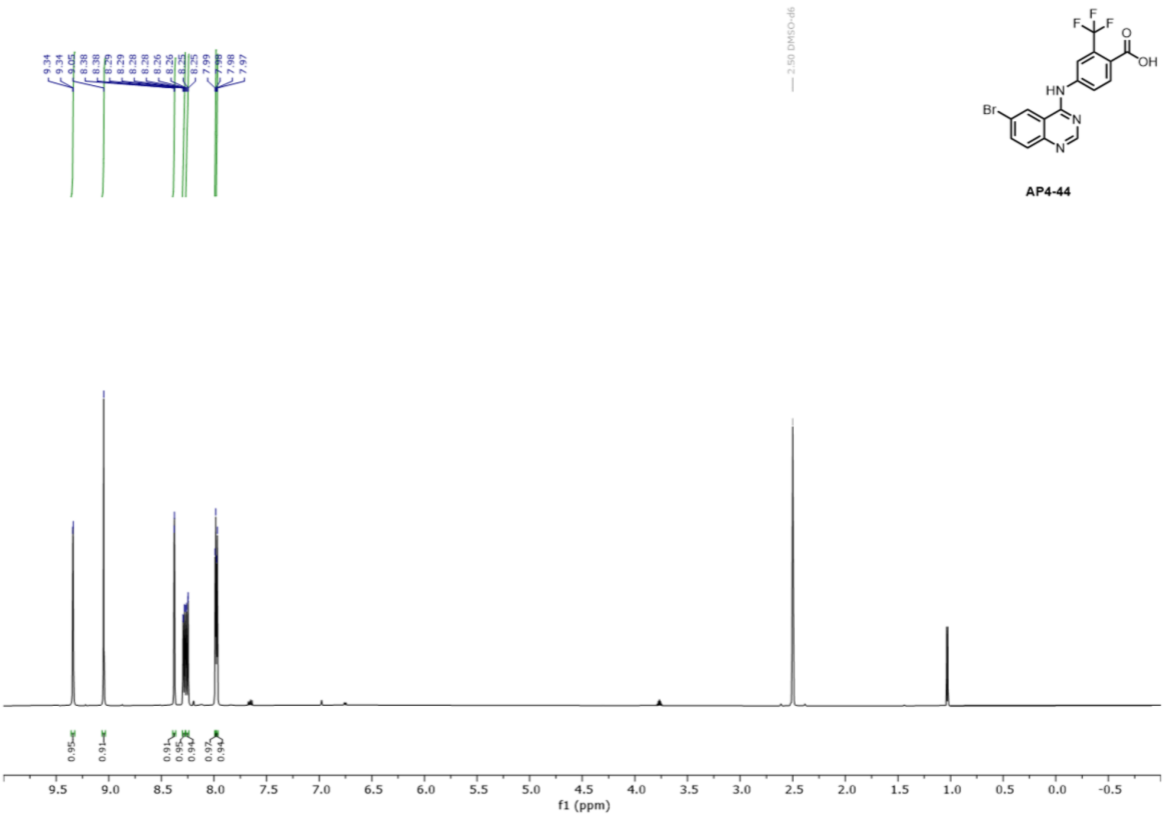

AP4-44 – ^{13}C NMR (151 MHz, DMSO-*d6*)

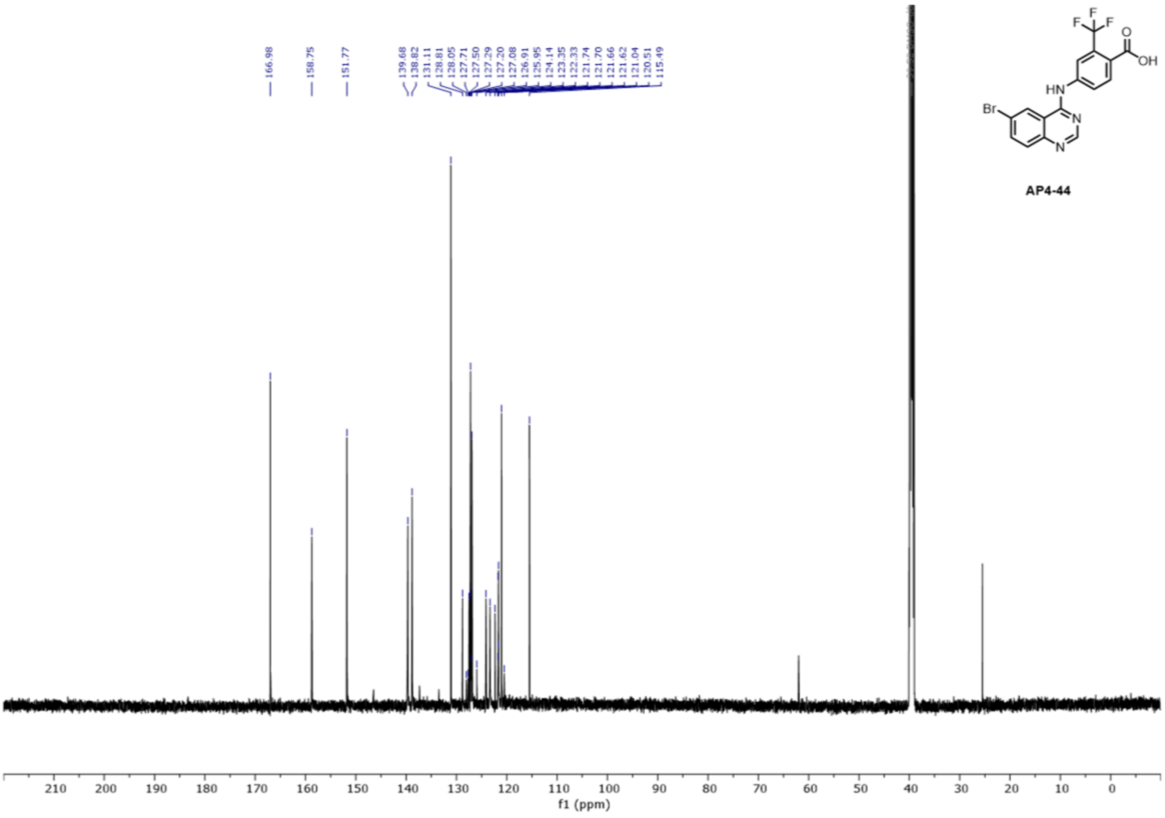

ELK-014A – ^{1}H NMR (600 MHz, DMSO-d_6)

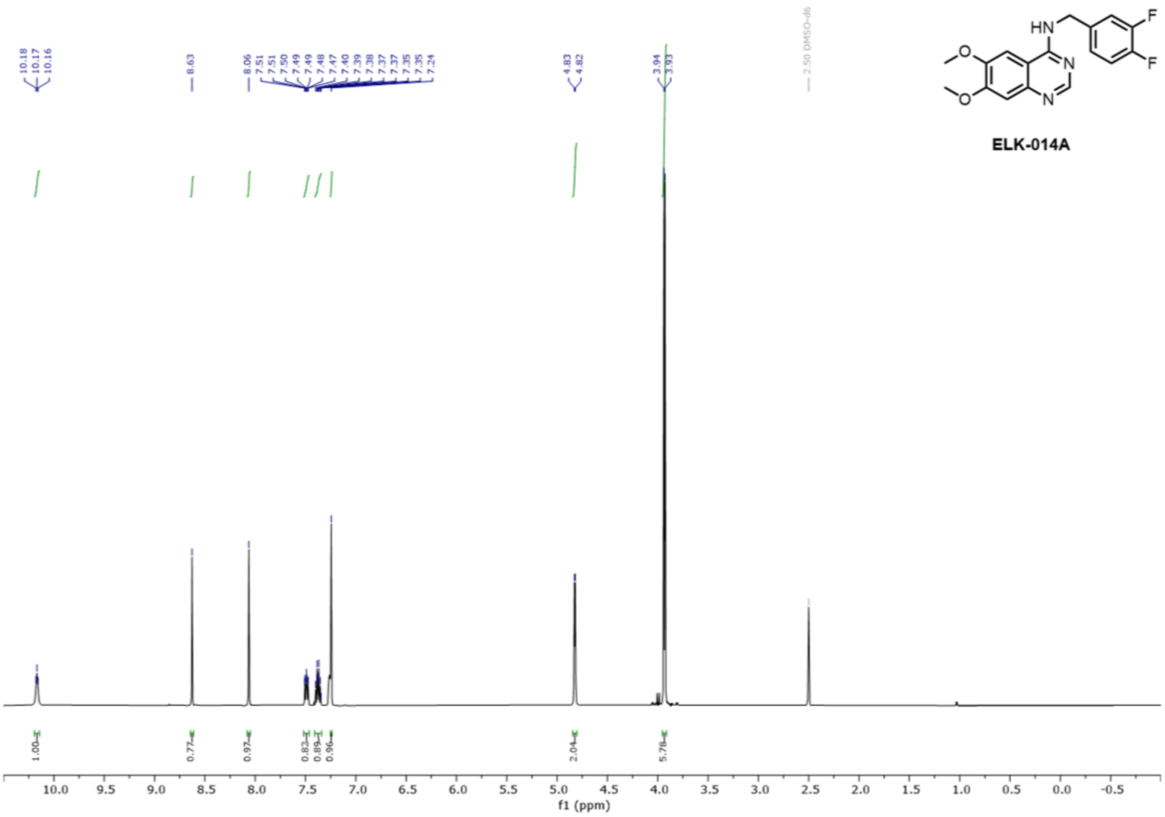

ELK-014A – ^{13}C NMR (151 MHz, DMSO-d_6)

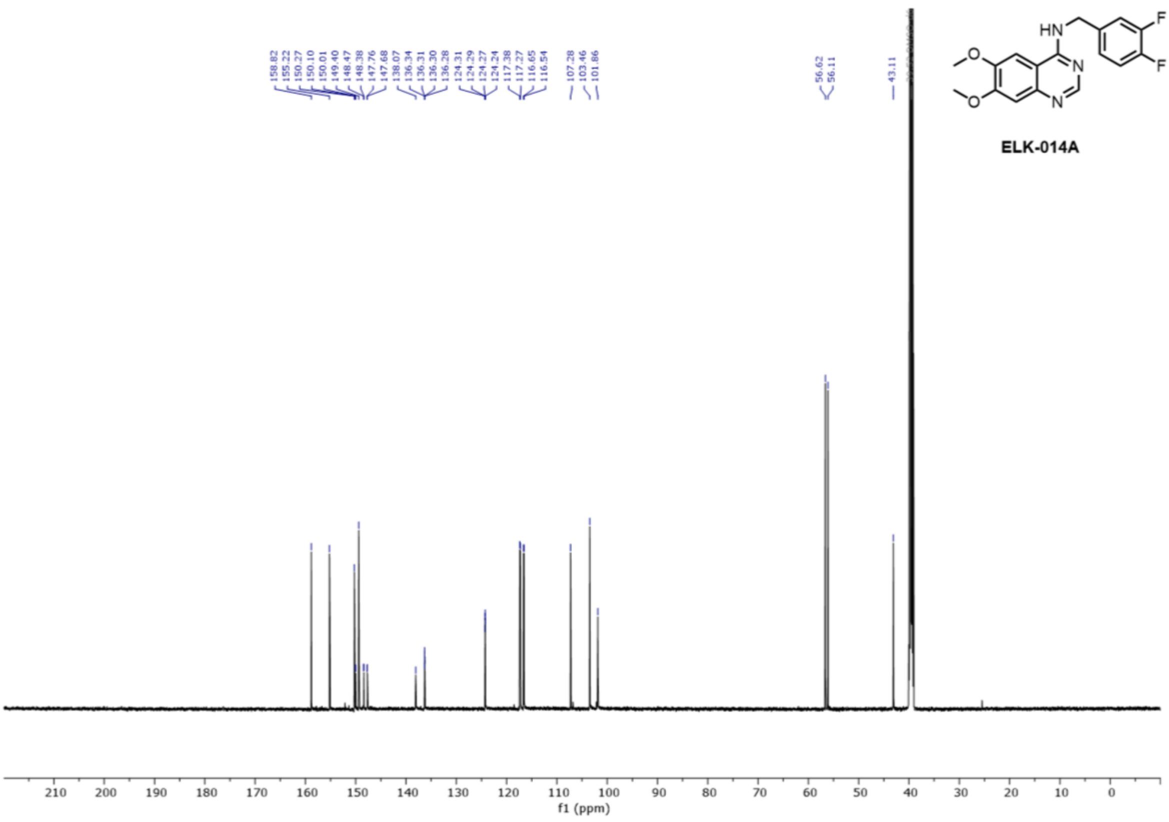

ELK-015A – ^{1}H NMR (600 MHz, DMSO-*d6*)

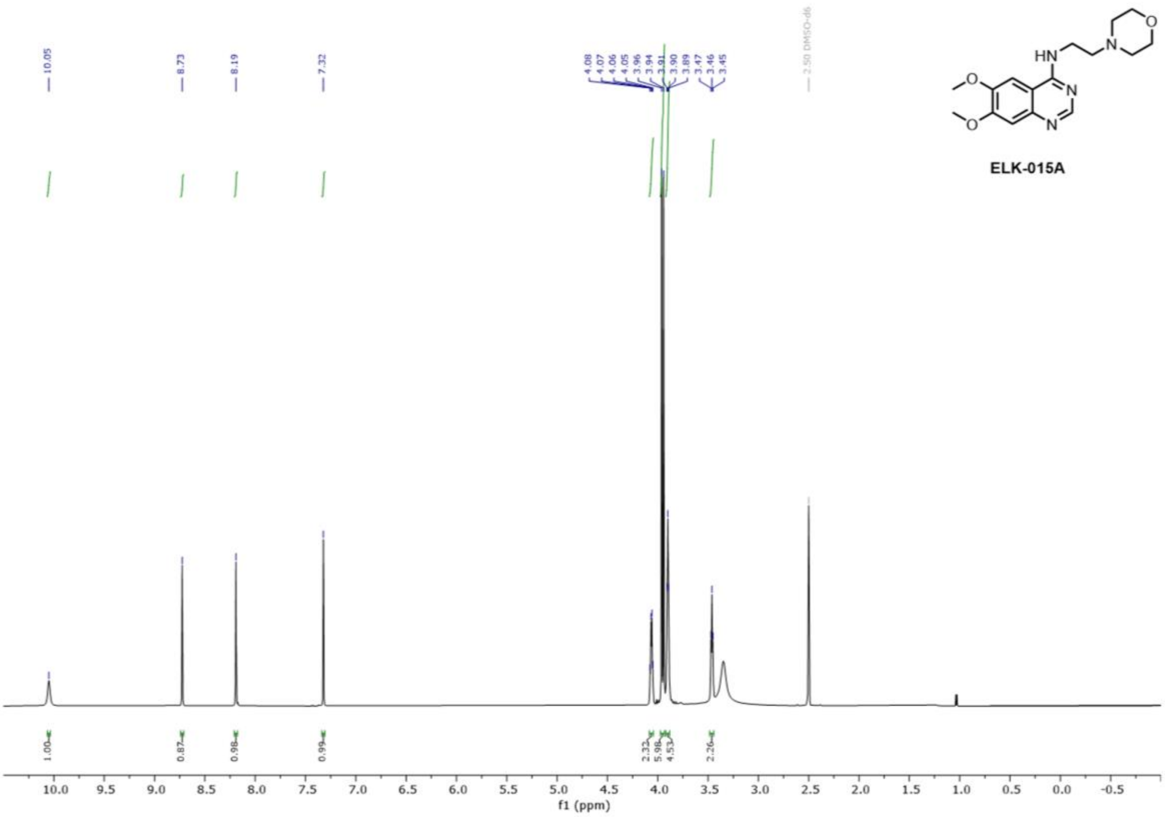

ELK-015A – ^{13}C NMR (151 MHz, DMSO-*d6*)

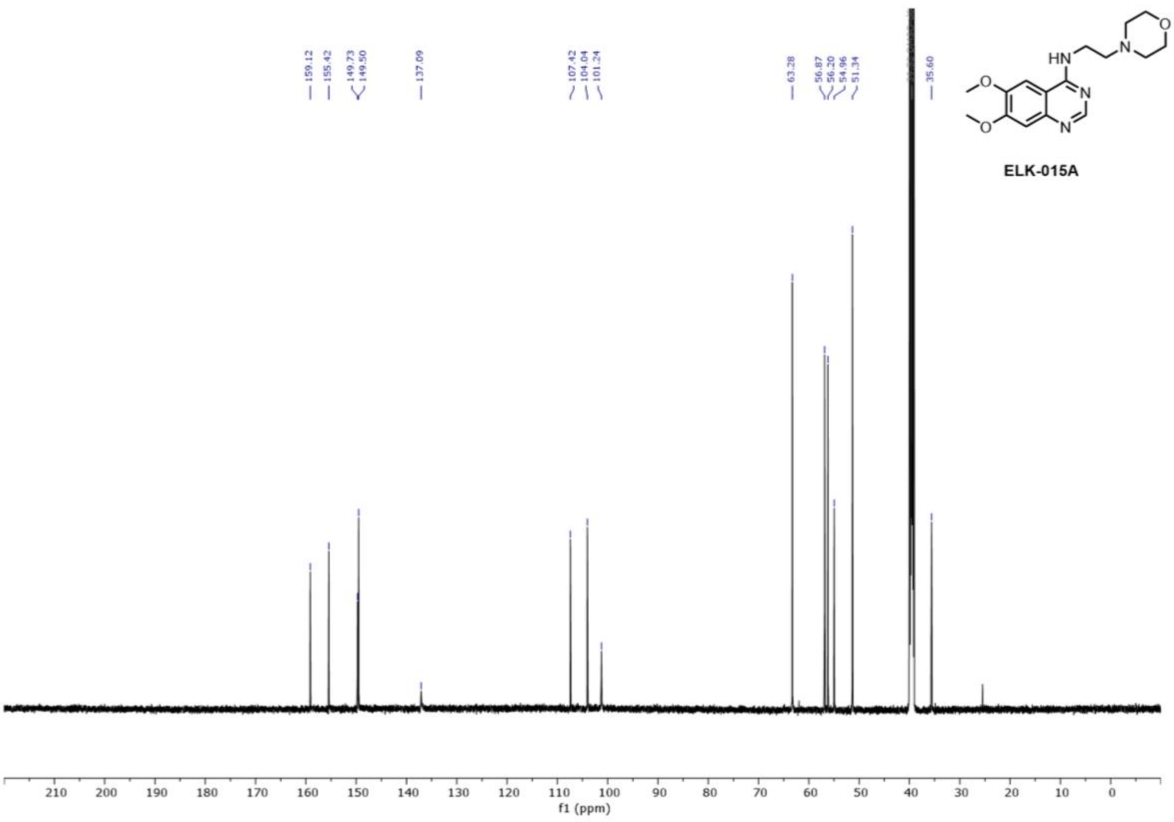

ELK-016A – ^{1}H NMR (600 MHz, DMSO-*d*$_6$)

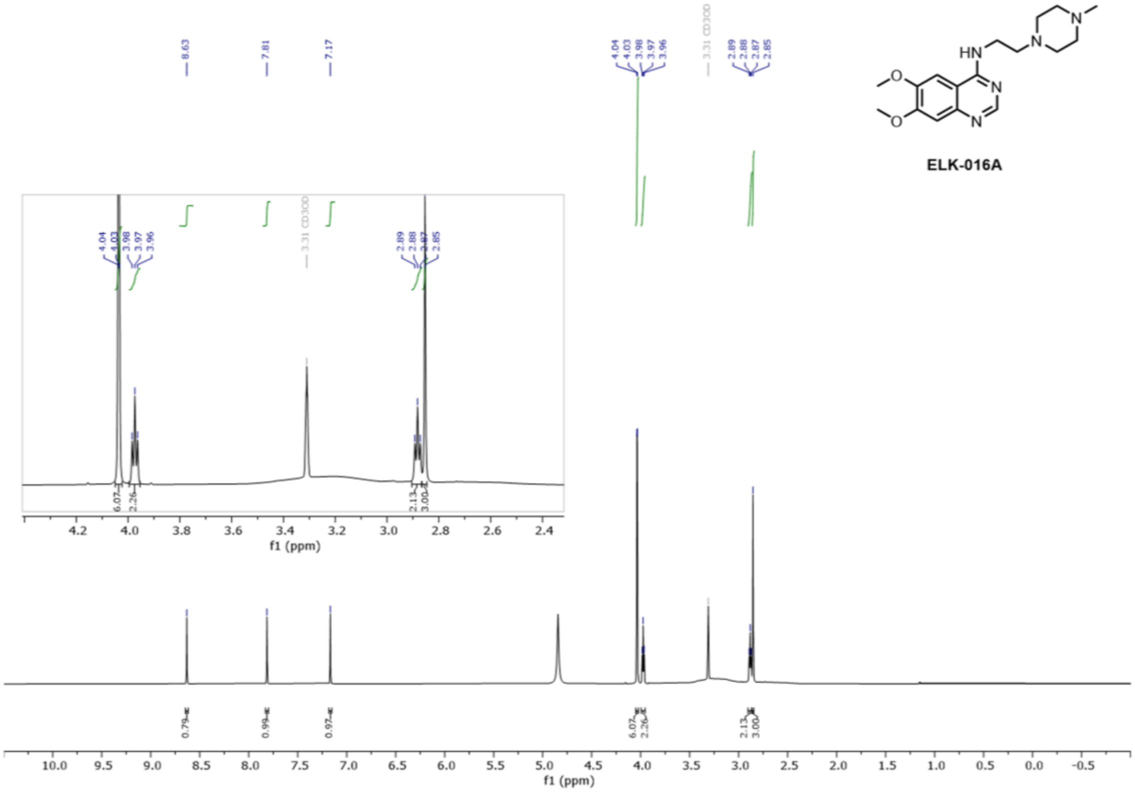

ELK-016A – ^{13}C NMR (151 MHz, DMSO-*d*$_6$)

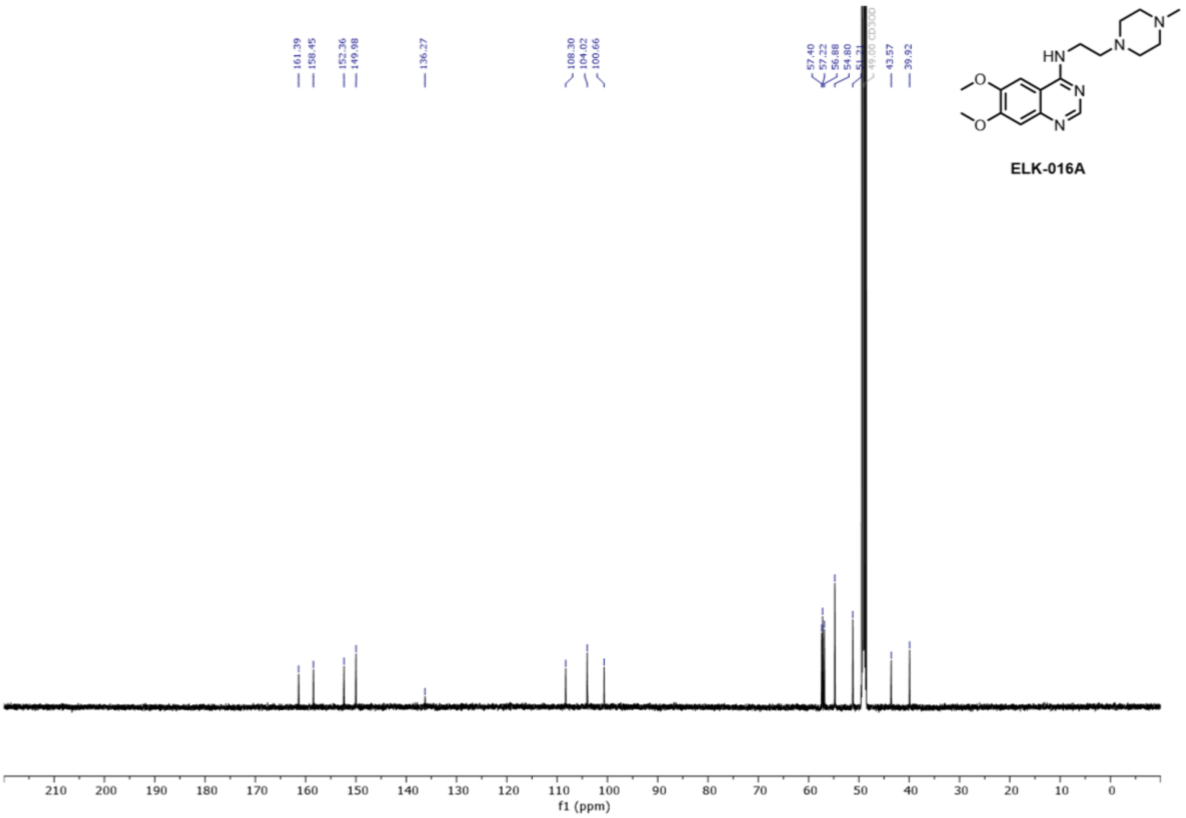

ELK-019A – ^{1}H NMR (600 MHz, DMSO-*d6*)

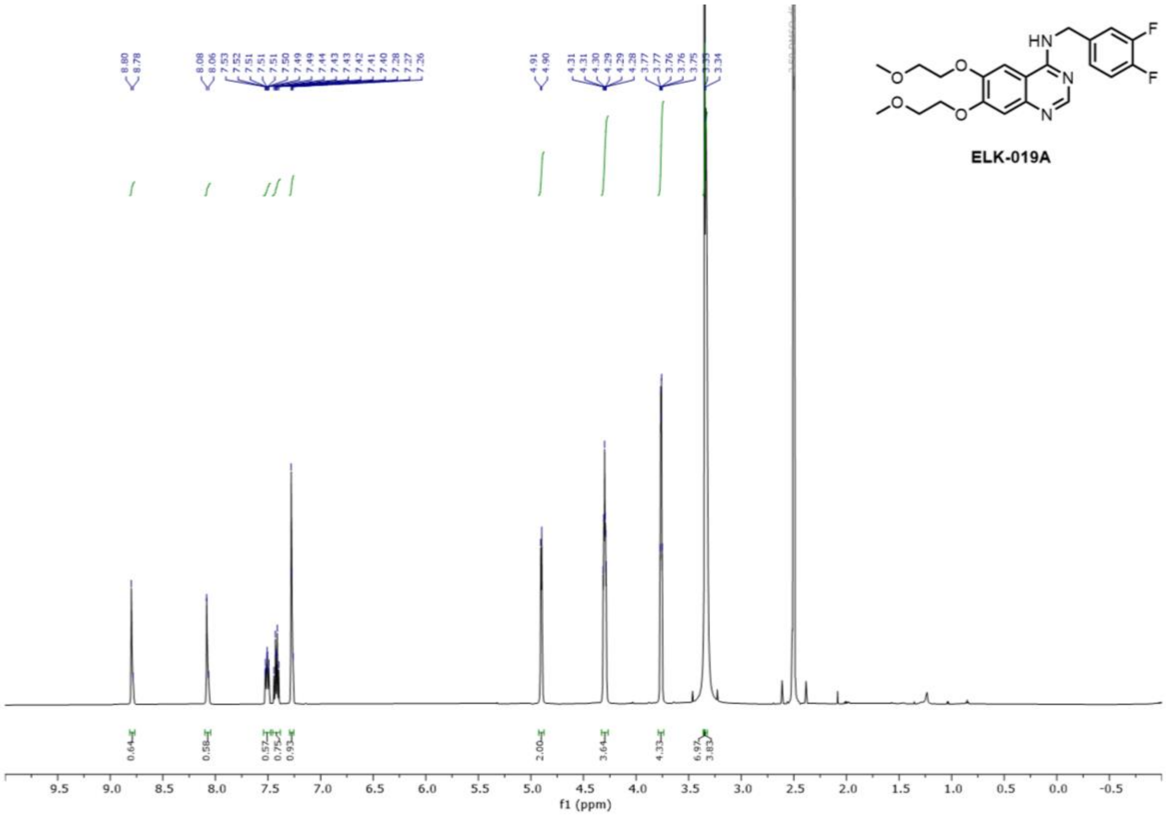

ELK-019A – ^{13}C NMR (151 MHz, DMSO-*d6*)

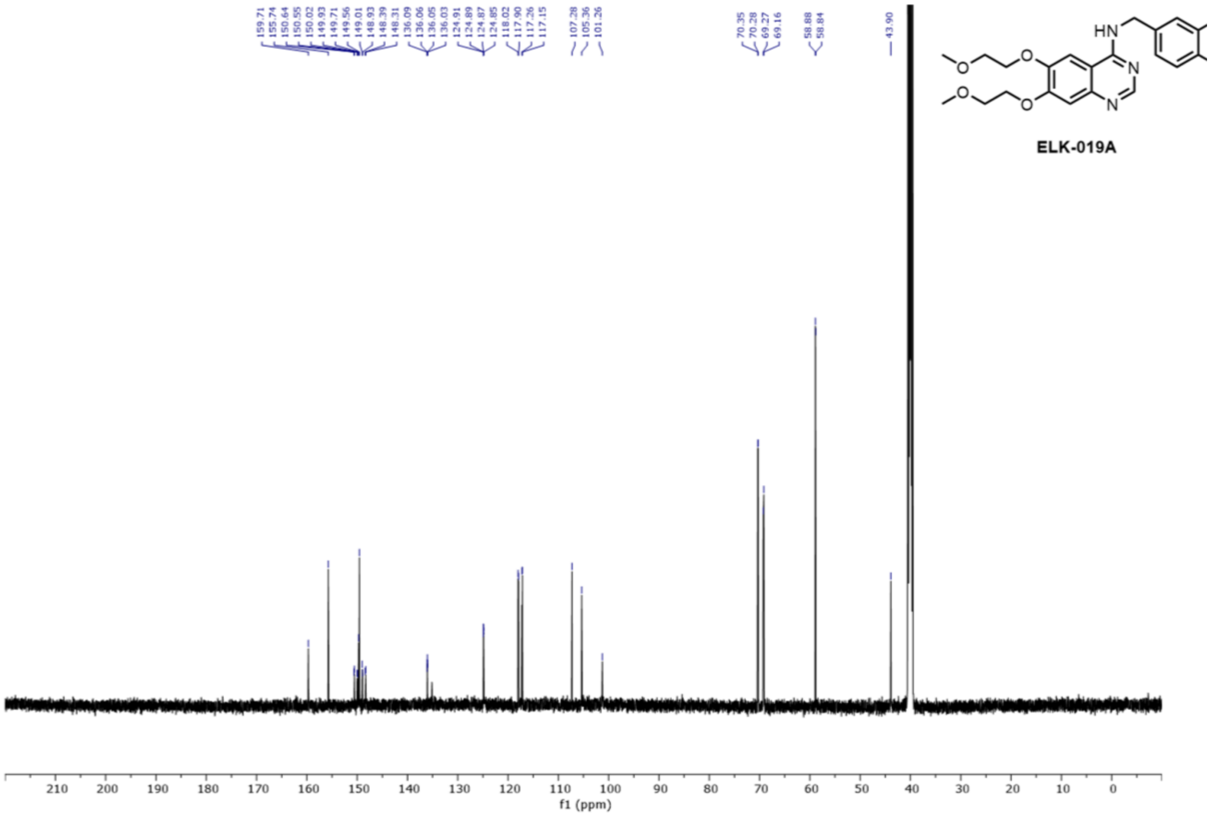

ELK-021A – ^{1}H NMR (600 MHz, DMSO-*d6*)

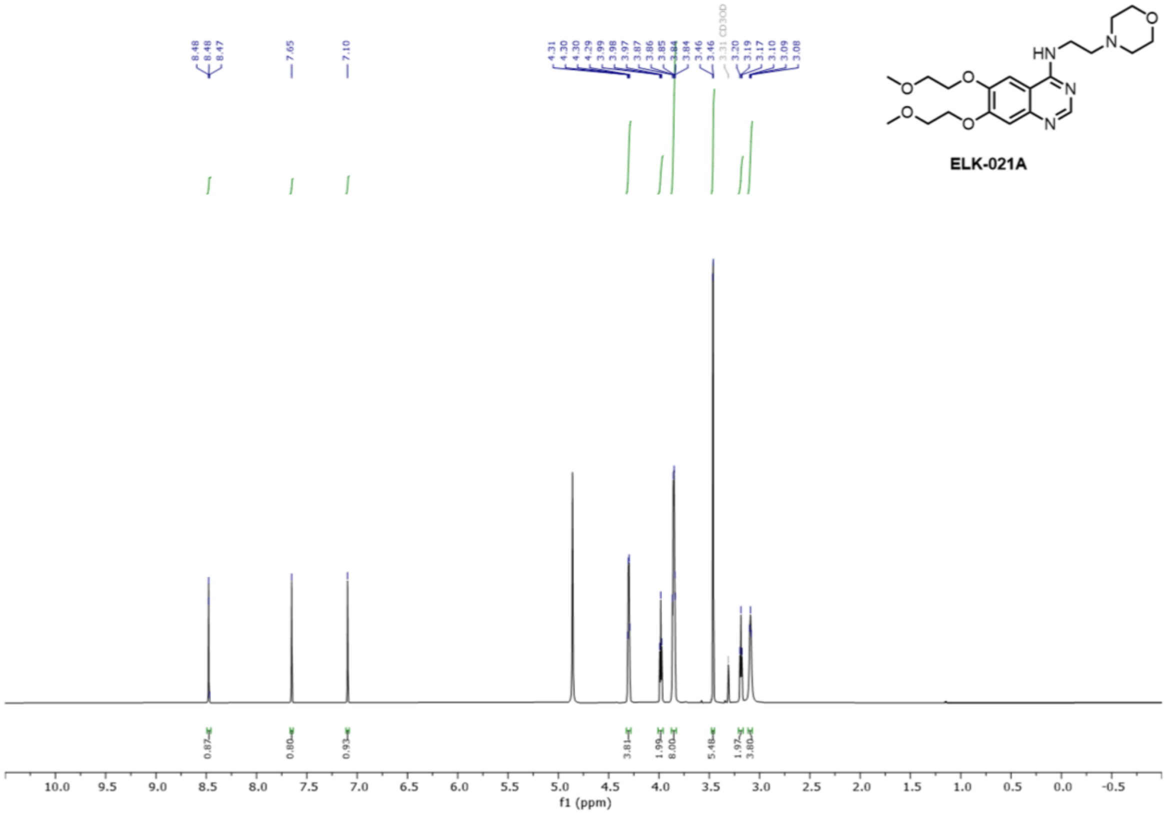

ELK-021A – ^{13}C NMR (151 MHz, DMSO-*d6*)

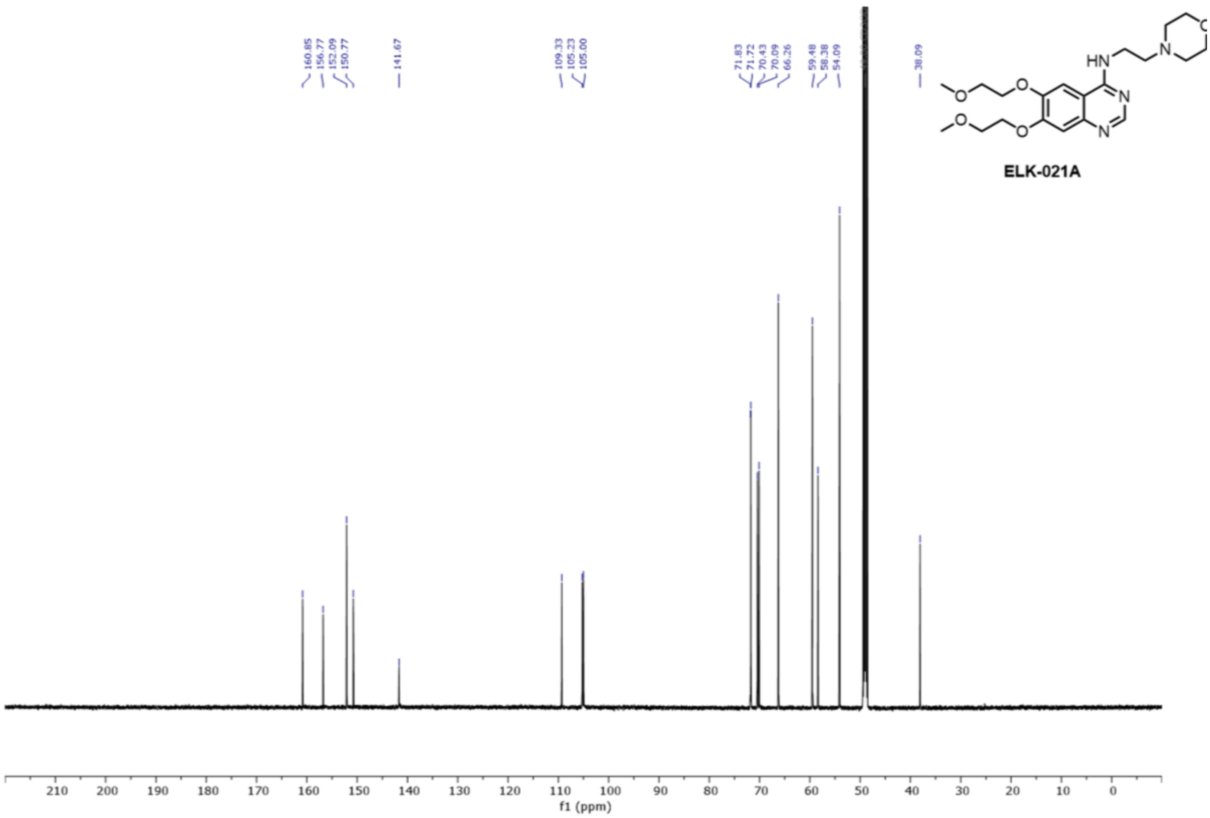

ELK-022A – ^{1}H NMR (600 MHz, DMSO-d_6)

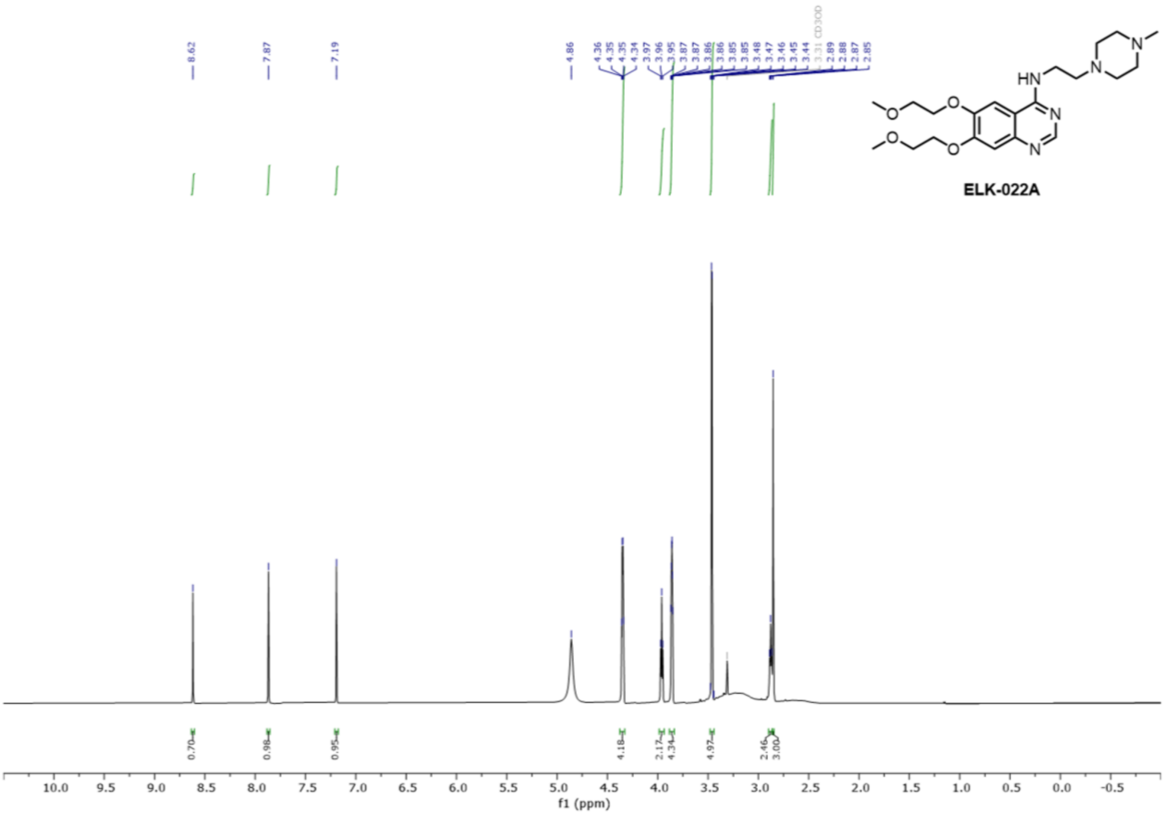

ELK-022A – ^{13}C NMR (151 MHz, DMSO-d_6)

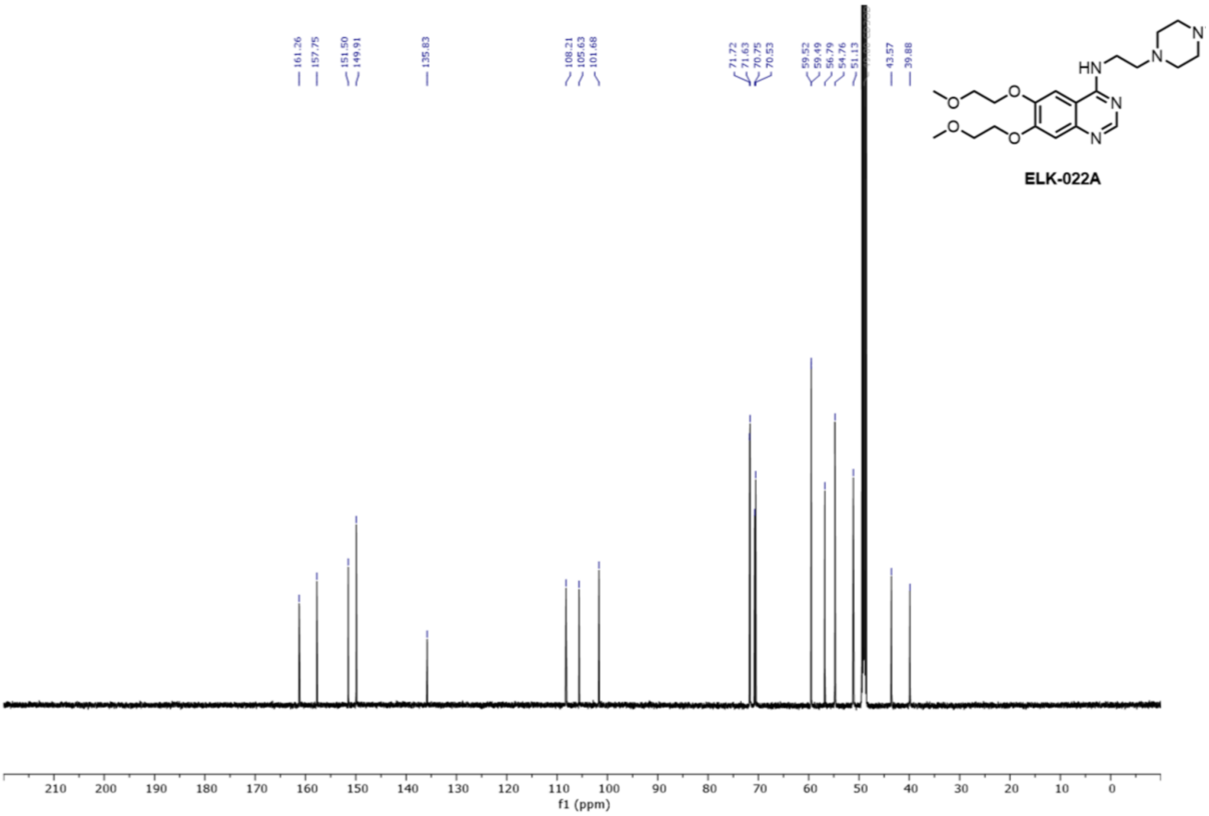

ELK-023A – ^{1}H NMR (600 MHz, DMSO-d_6)

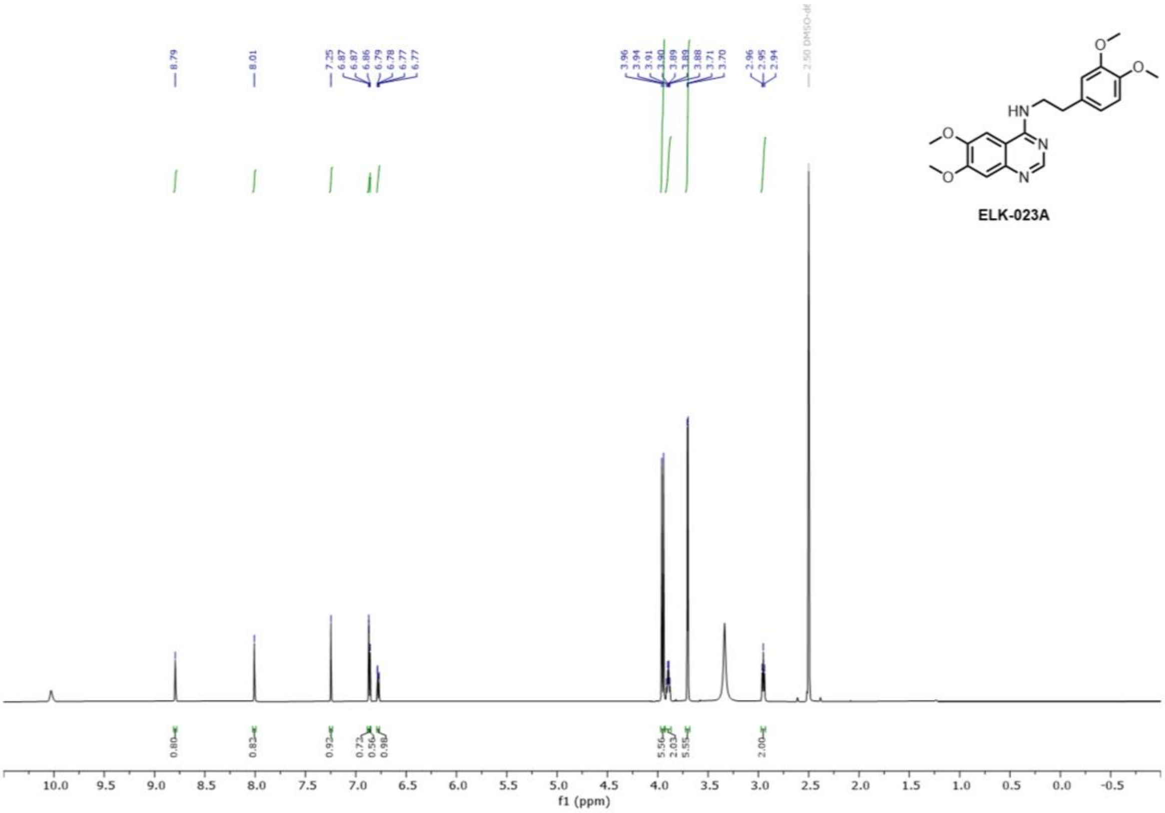

ELK-023A – ^{13}C NMR (151 MHz, DMSO-d_6)

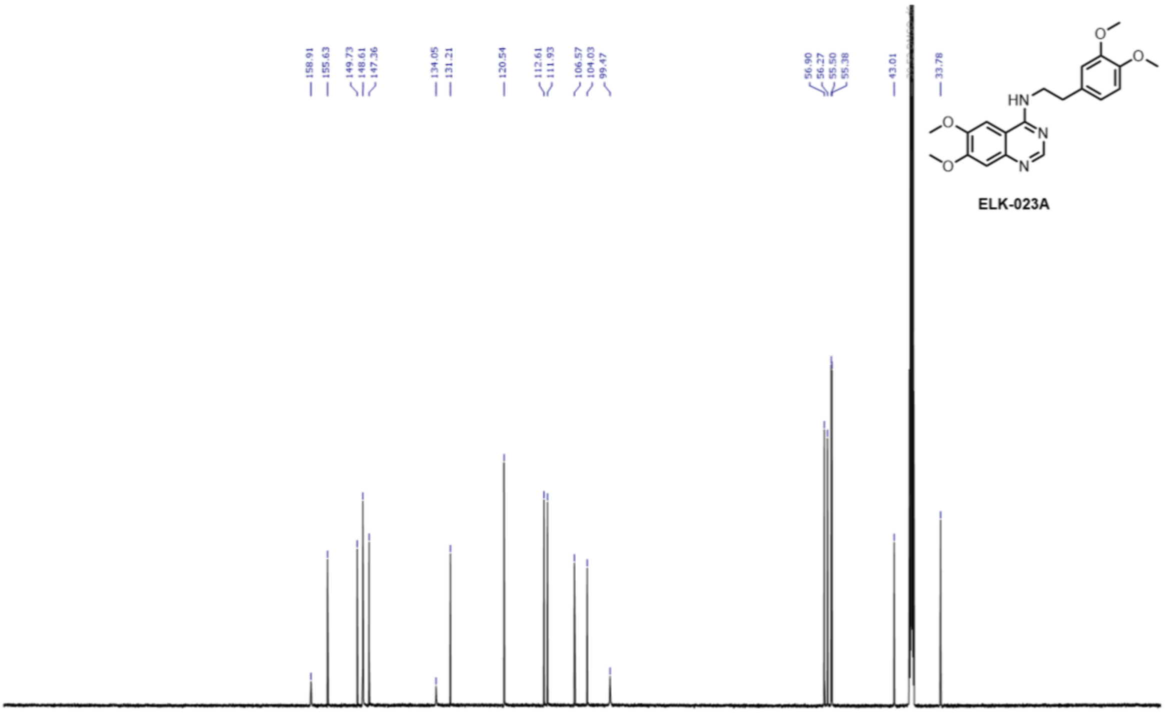

ELK-038A – ^{1}H NMR (600 MHz, DMSO-*d*$_6$)

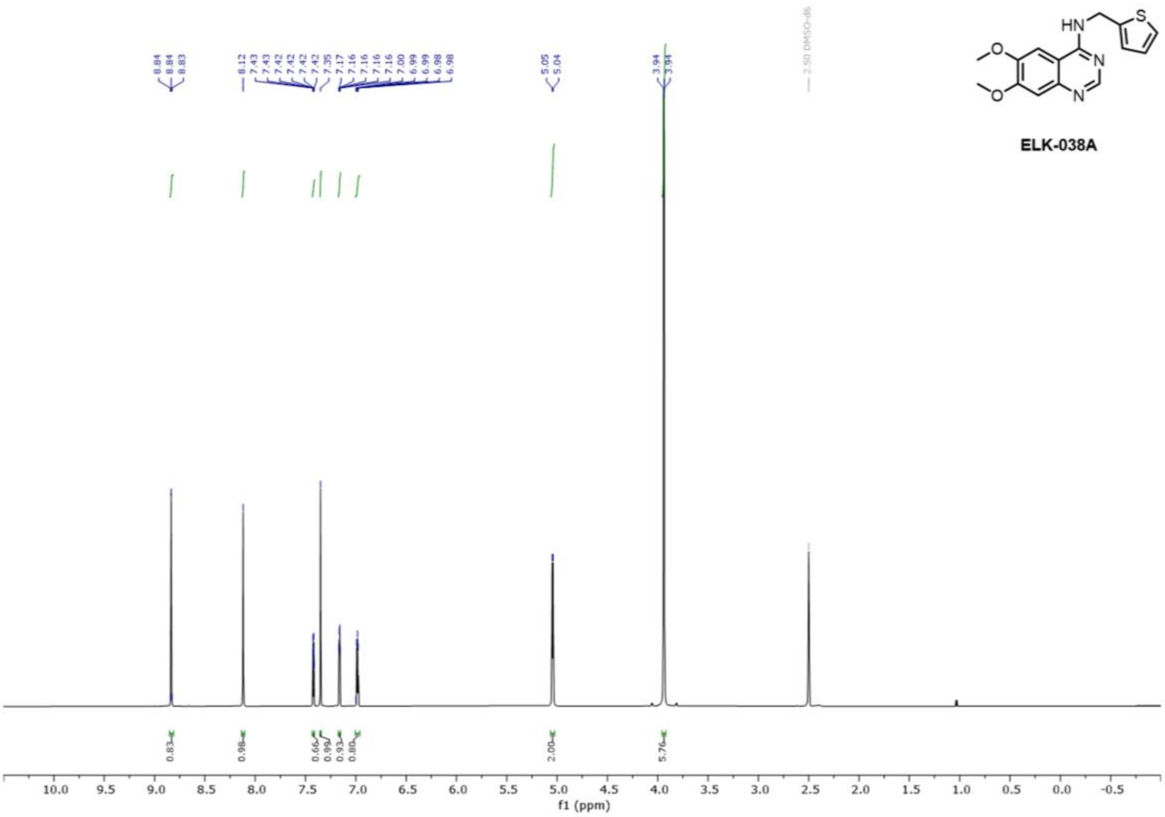

ELK-038A – ^{13}C NMR (151 MHz, DMSO-*d*$_6$)

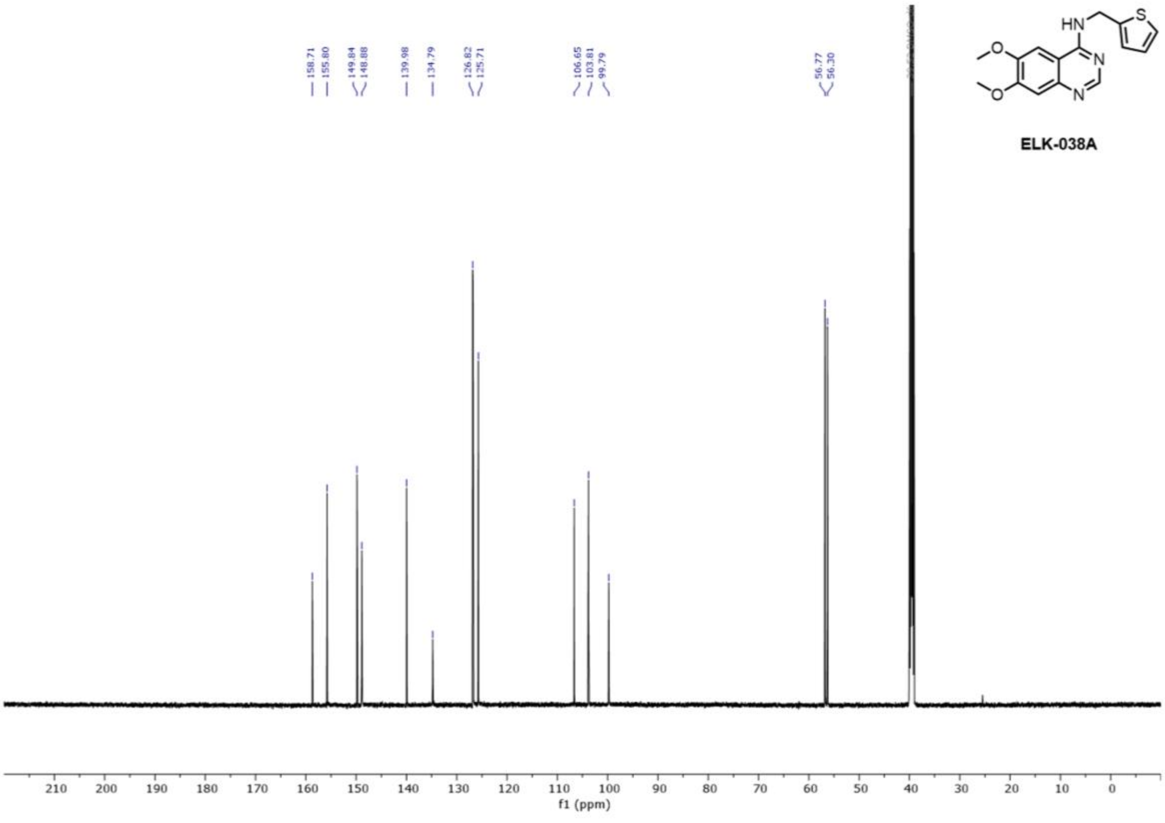

ELK-039B – ^{1}H NMR (600 MHz, DMSO-*d*$_6$)

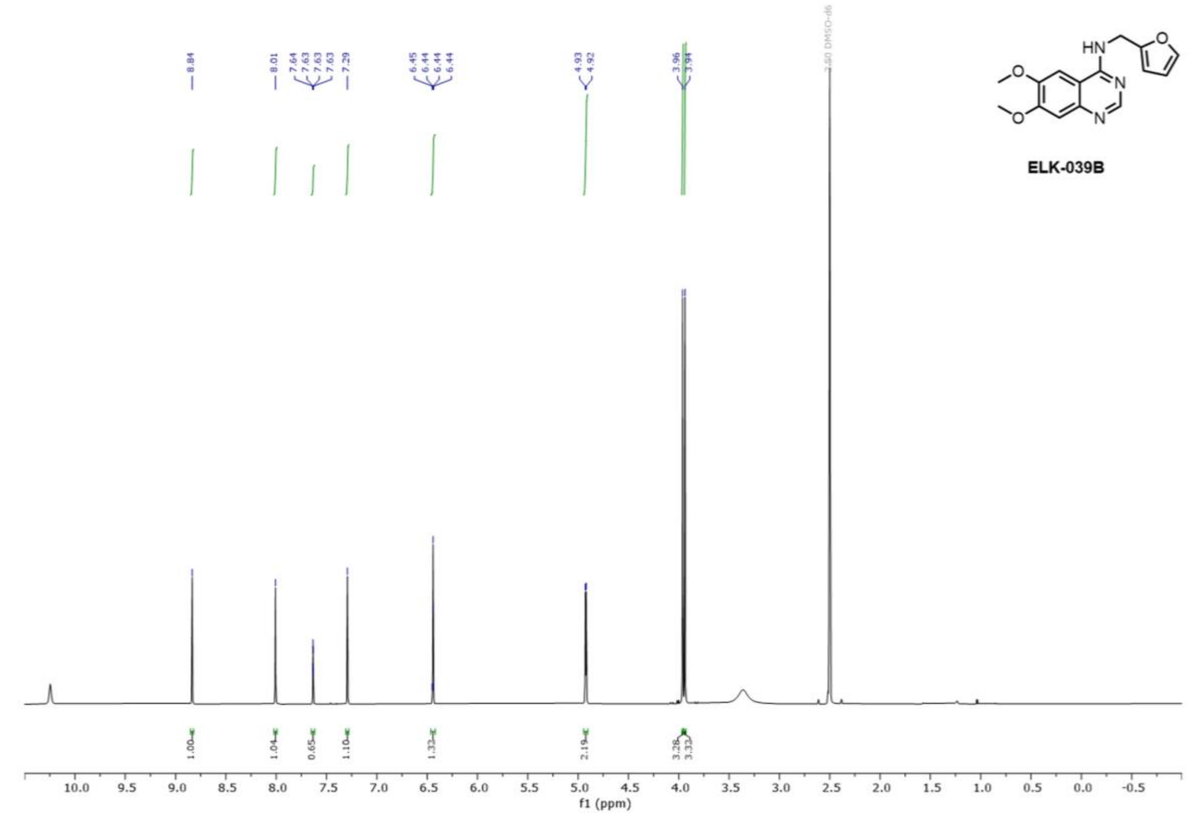

ELK-039B – ^{13}C NMR (151 MHz, DMSO-*d*$_6$)

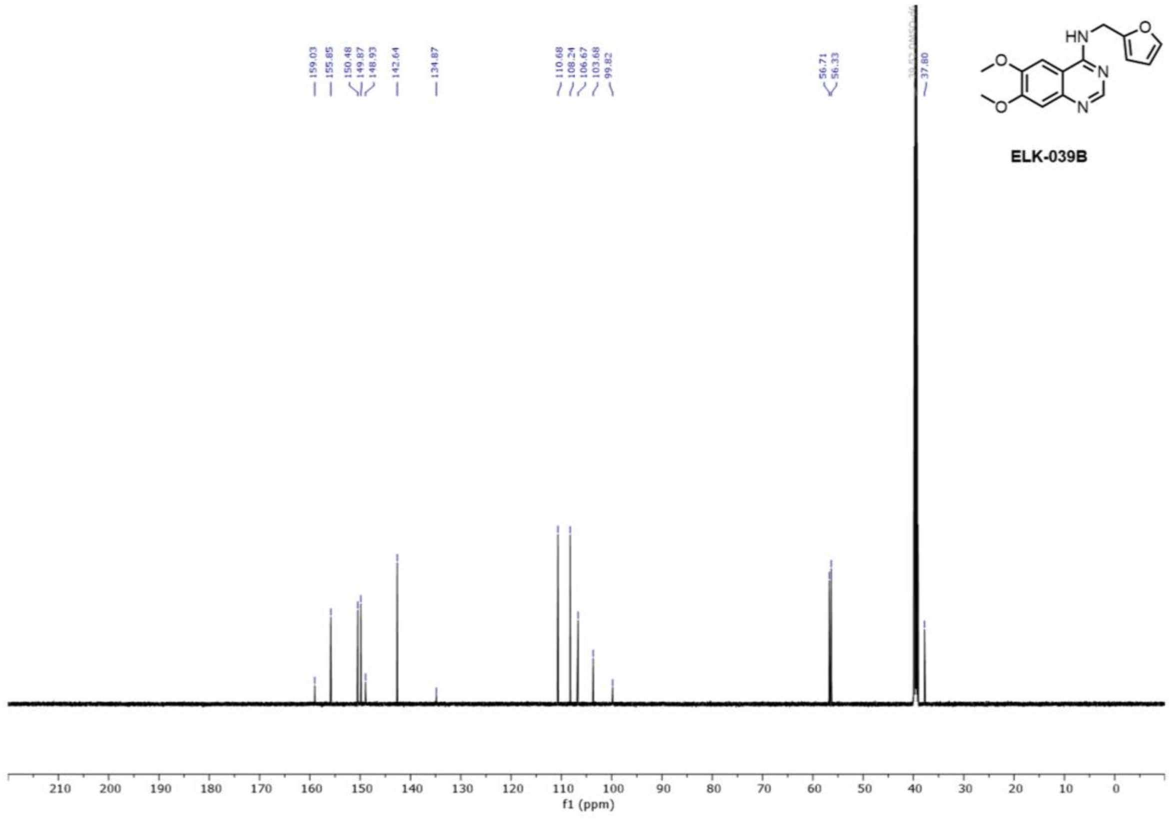

ELK-40C – ^{1}H NMR (600 MHz, DMSO-*d6*)

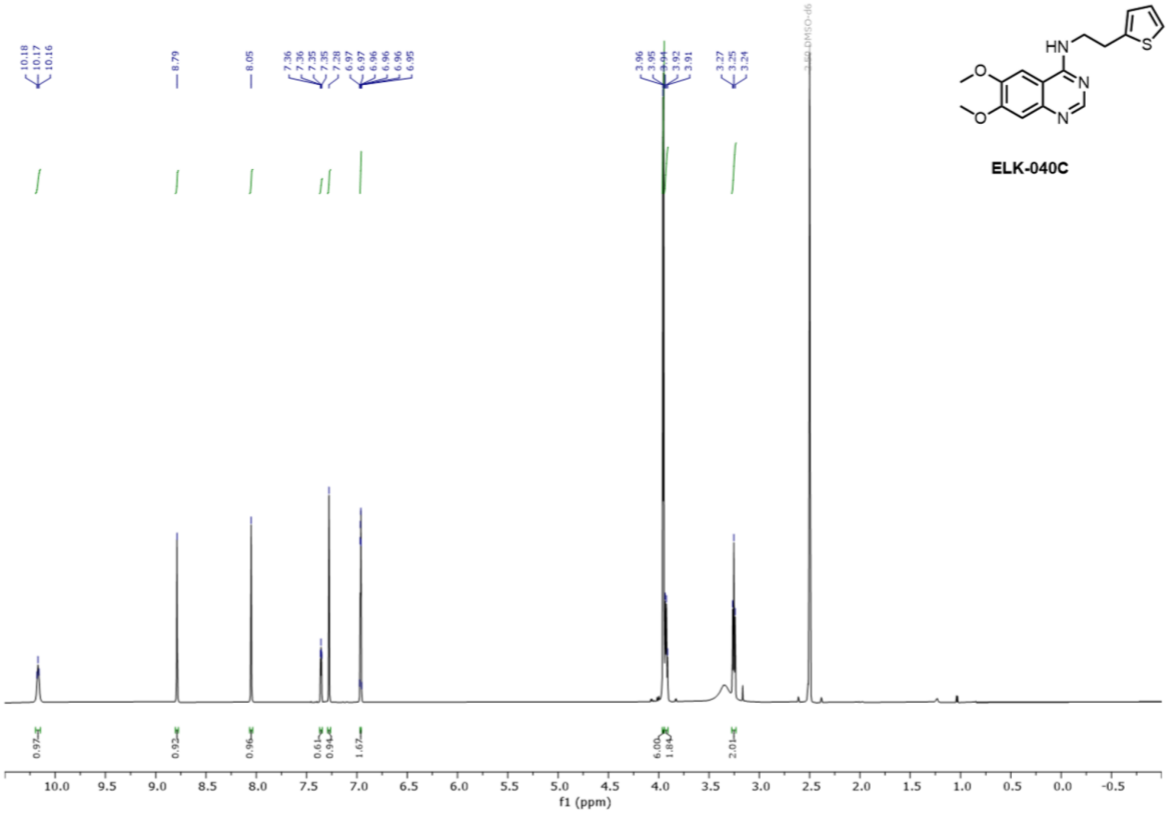

ELK-40C – ^{13}C NMR (151 MHz, DMSO-*d6*)

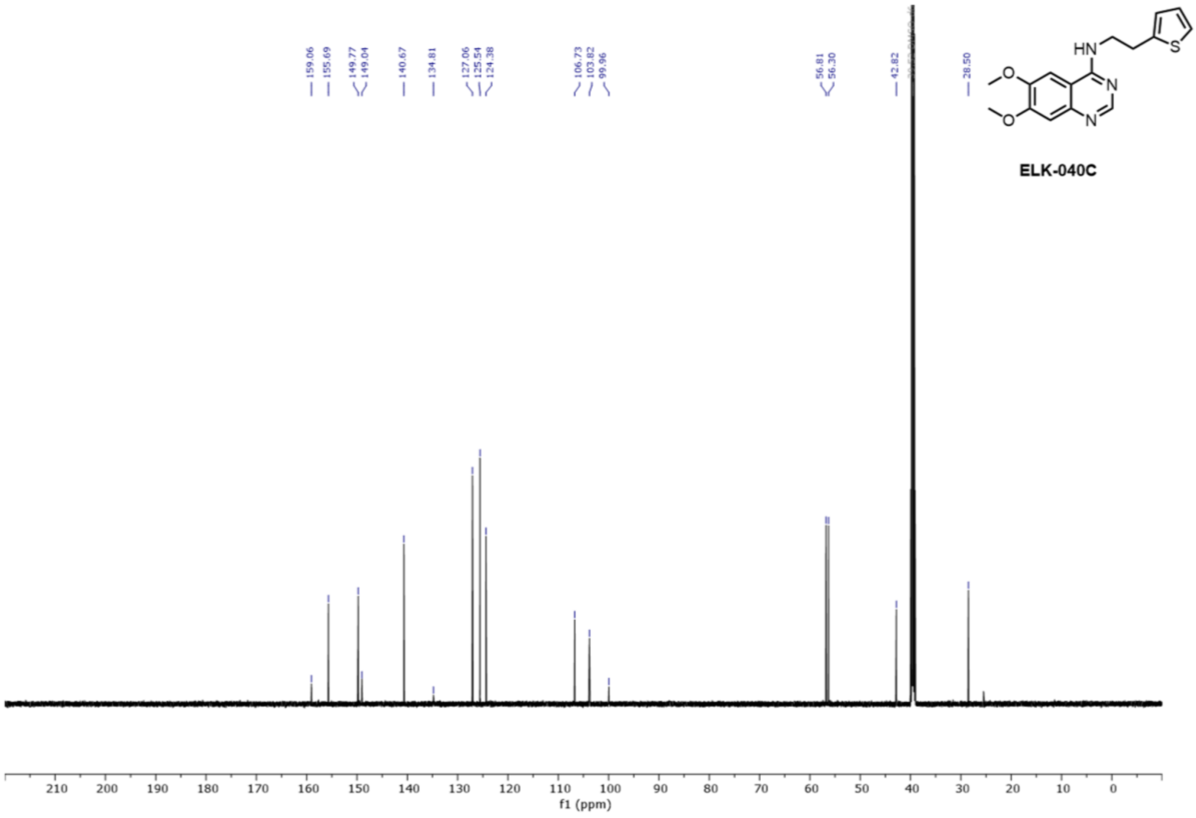

ELK-43C – ^{1}H NMR (600 MHz, DMSO-*d6*)

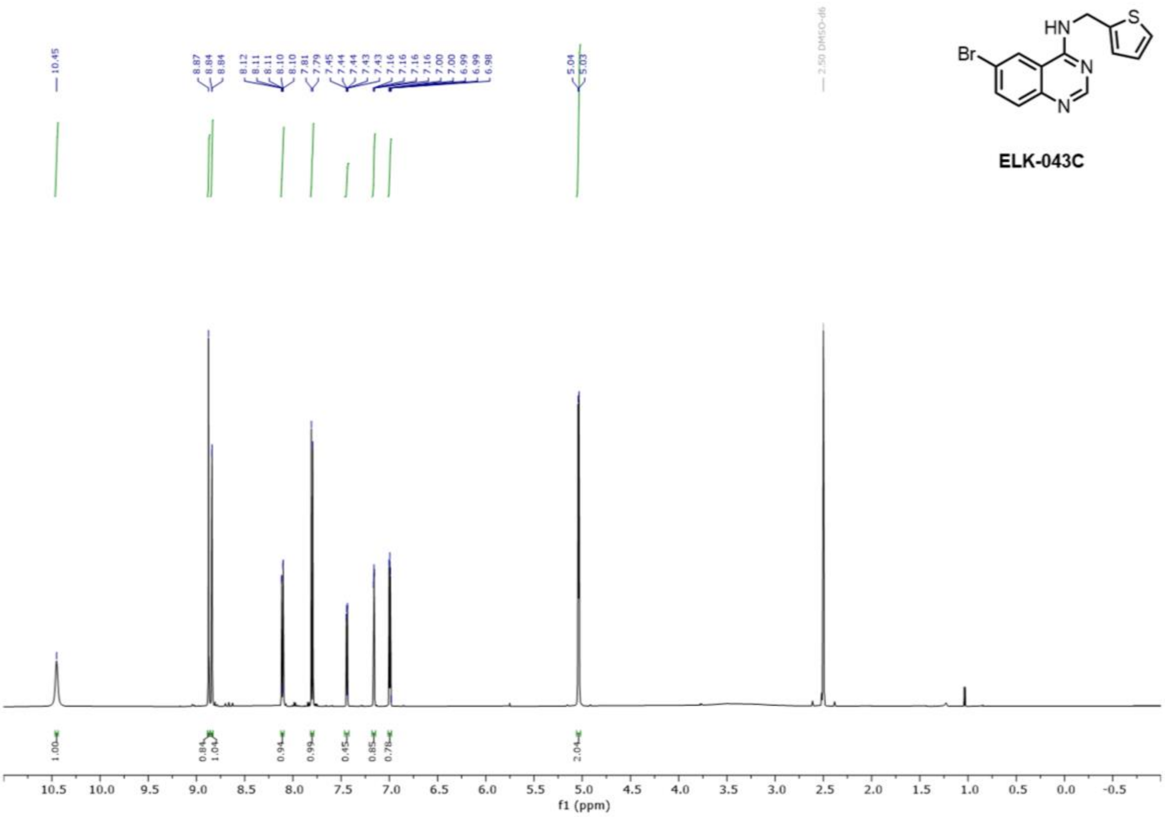

ELK-43C – ^{13}C NMR (151 MHz, DMSO-*d6*)

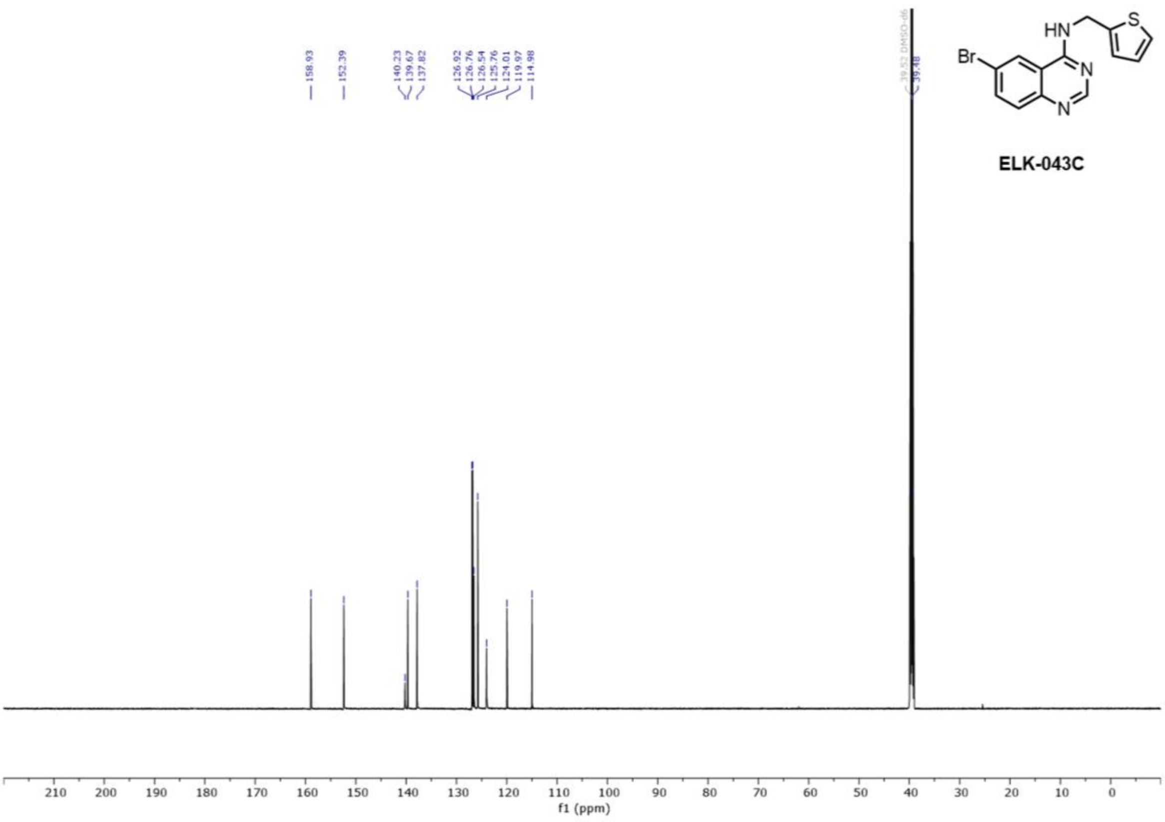

ELK-046C – ^{1}H NMR (600 MHz, DMSO-d_6)

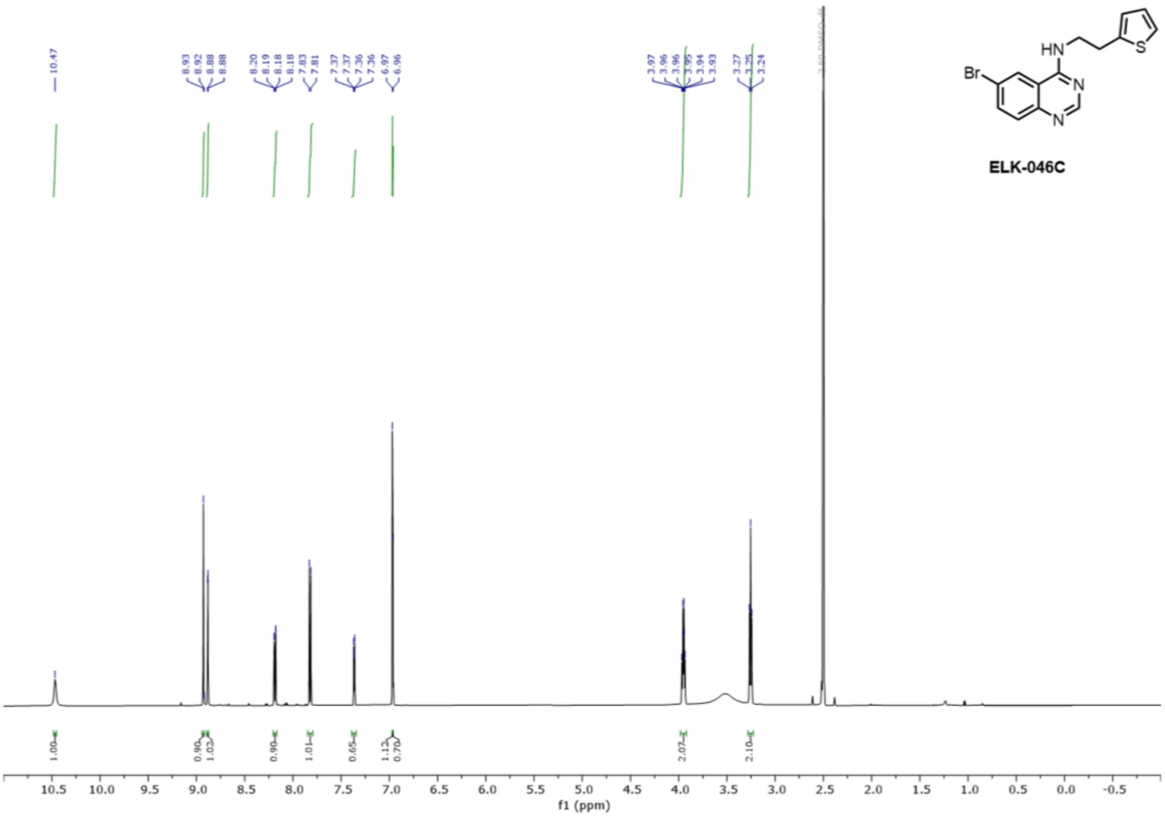

ELK-046C – ^{13}C NMR (151 MHz, DMSO-d_6)

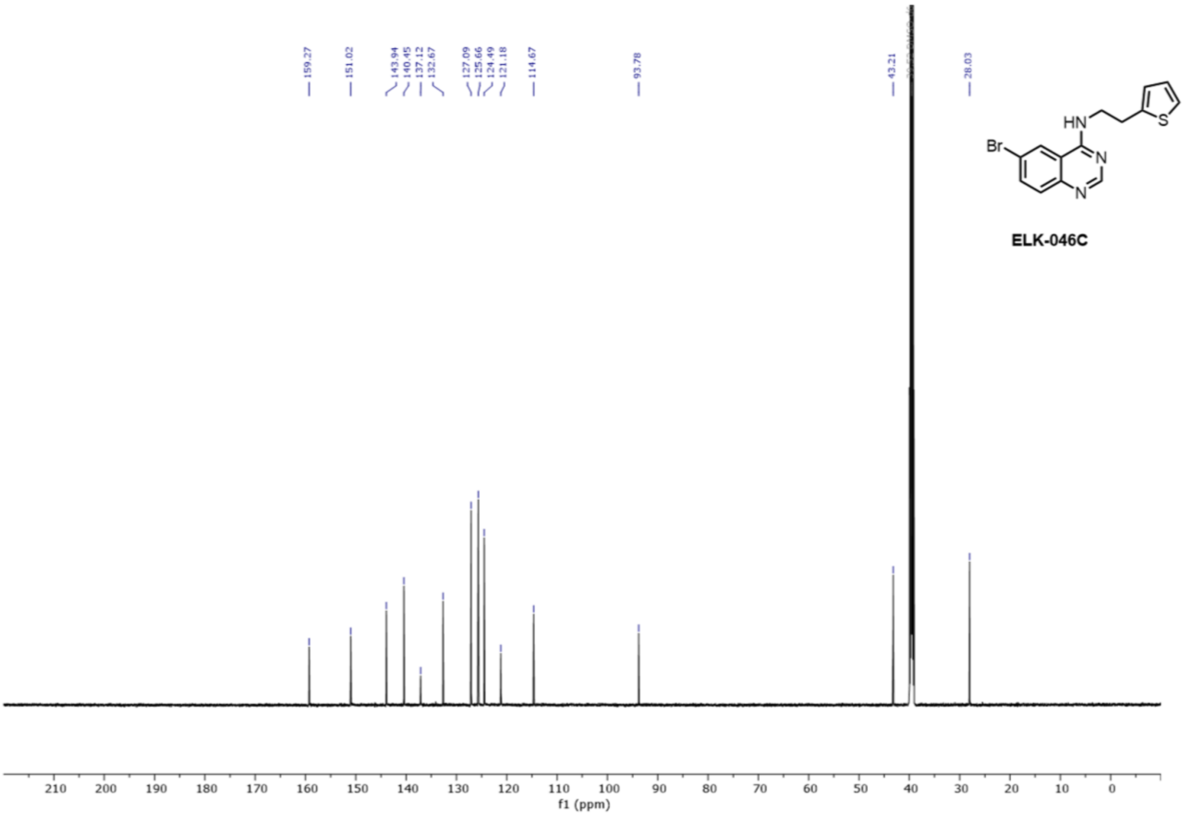

ELK-047A – ^{1}H NMR (600 MHz, DMSO-d_6)

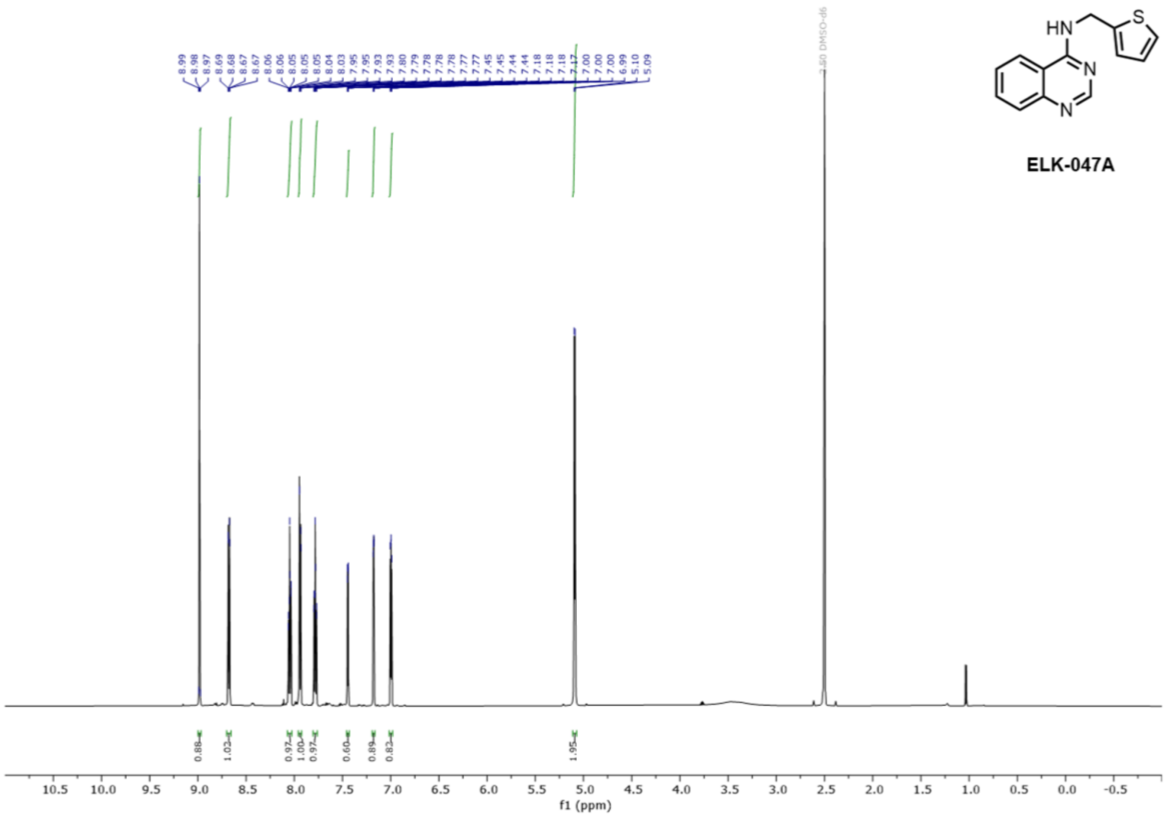

ELK-047A – ^{13}C NMR (151 MHz, DMSO-d_6)

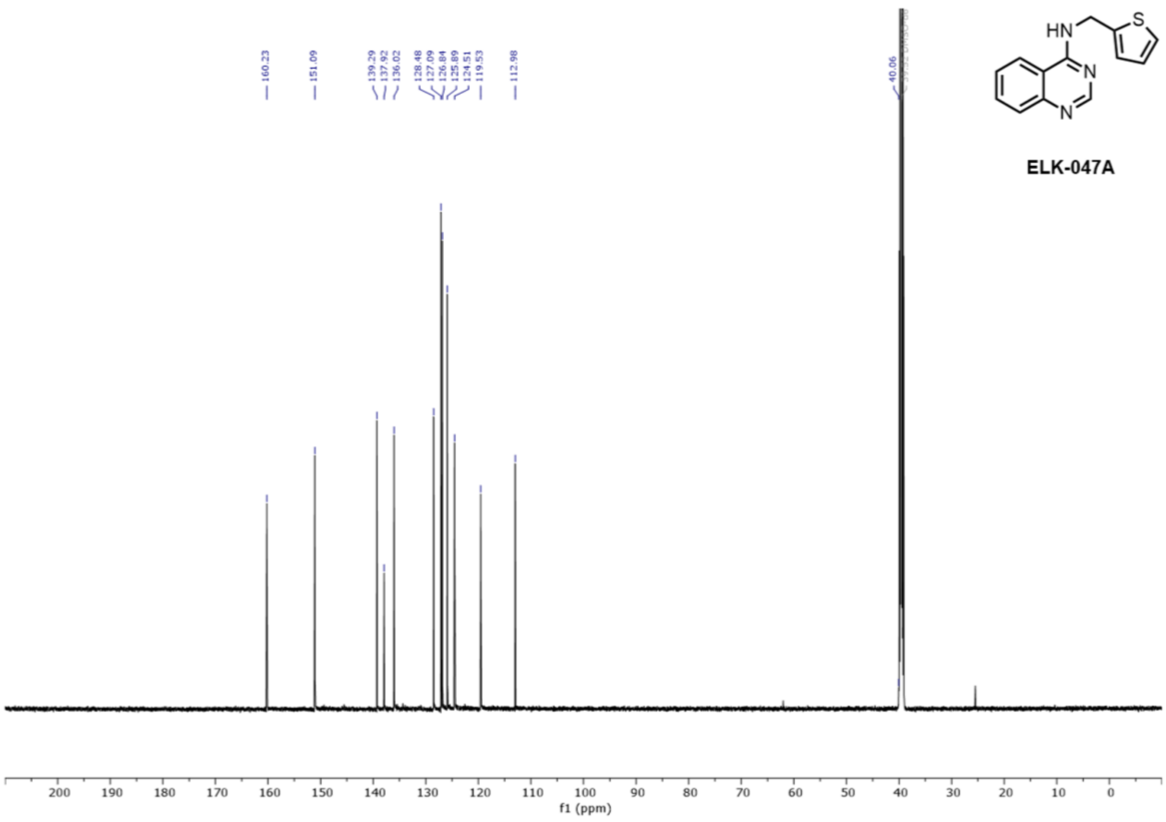

ELK-050A – ^{1}H NMR (600 MHz, DMSO-*d6*)

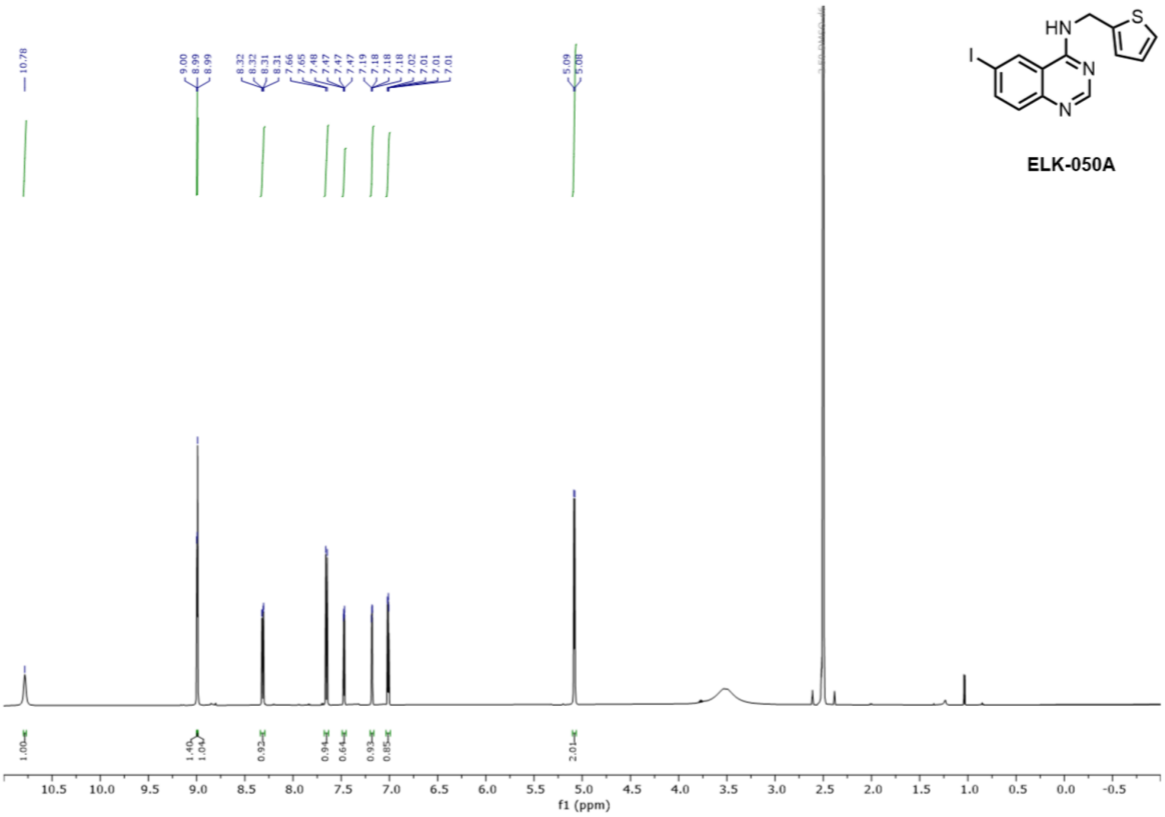

ELK-050A – ^{13}C NMR (151 MHz, DMSO-*d6*)

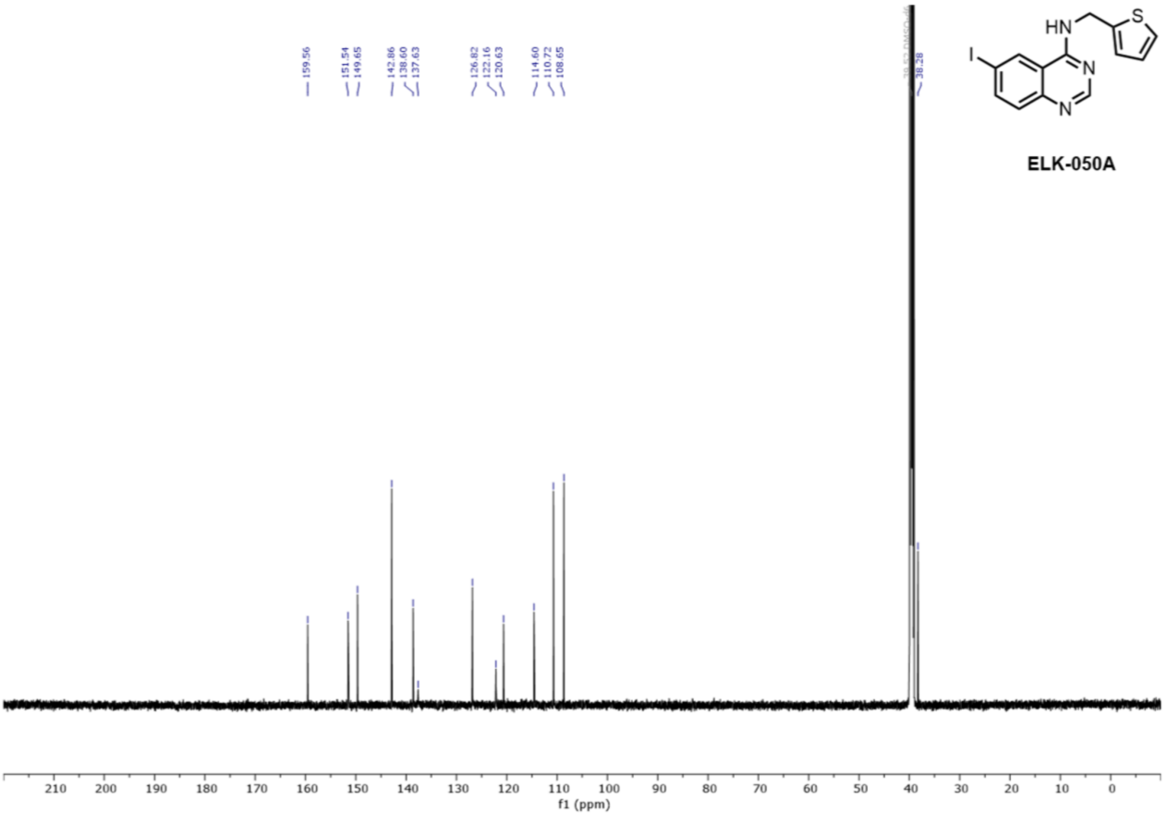

ELK-051A – ^{1}H NMR (600 MHz, DMSO-*d₆*)

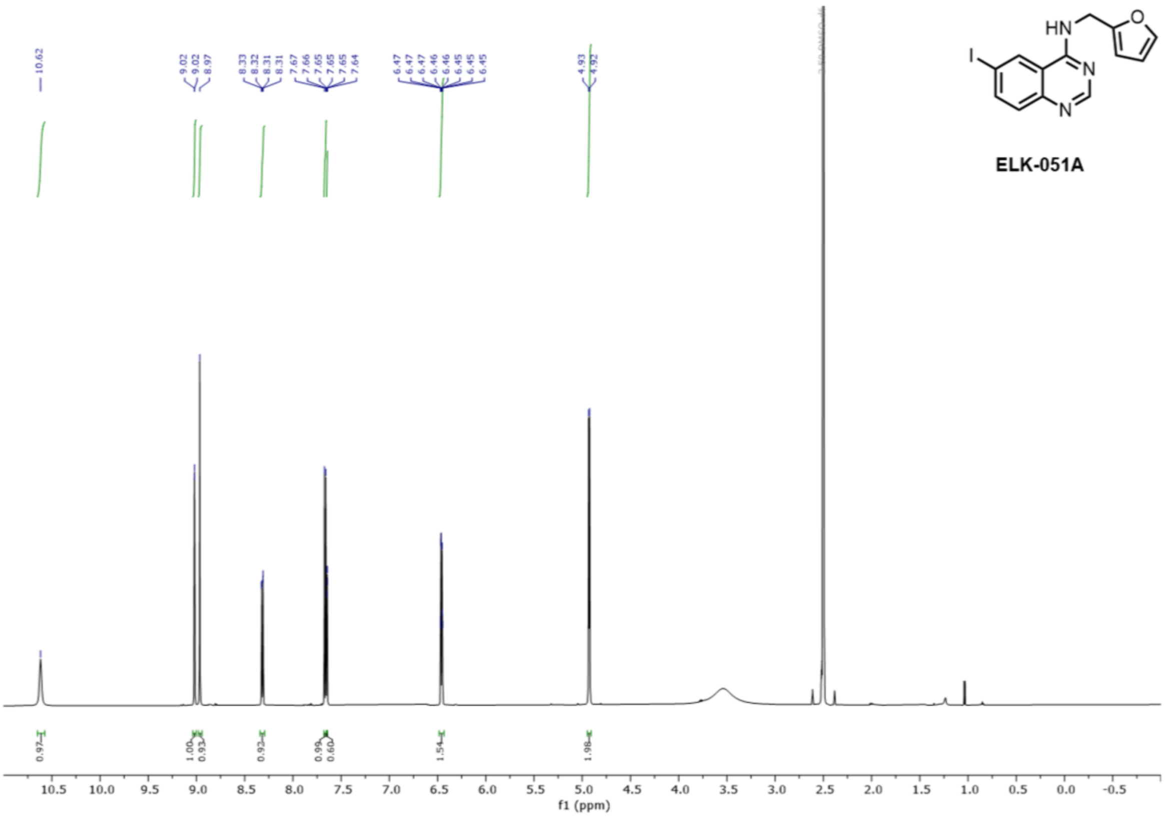

ELK-051A – ^{13}C NMR (151 MHz, DMSO-*d₆*)

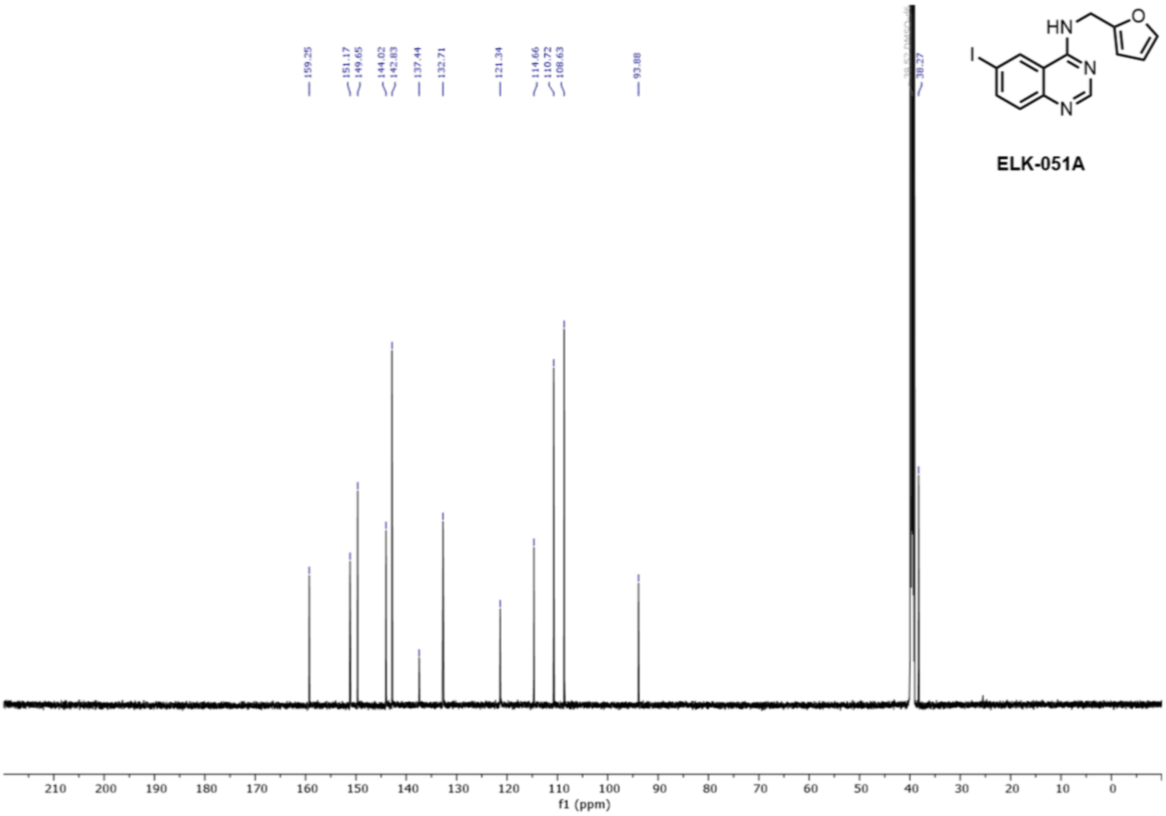

ELK-052A – ^{1}H NMR (600 MHz, DMSO-d_6)

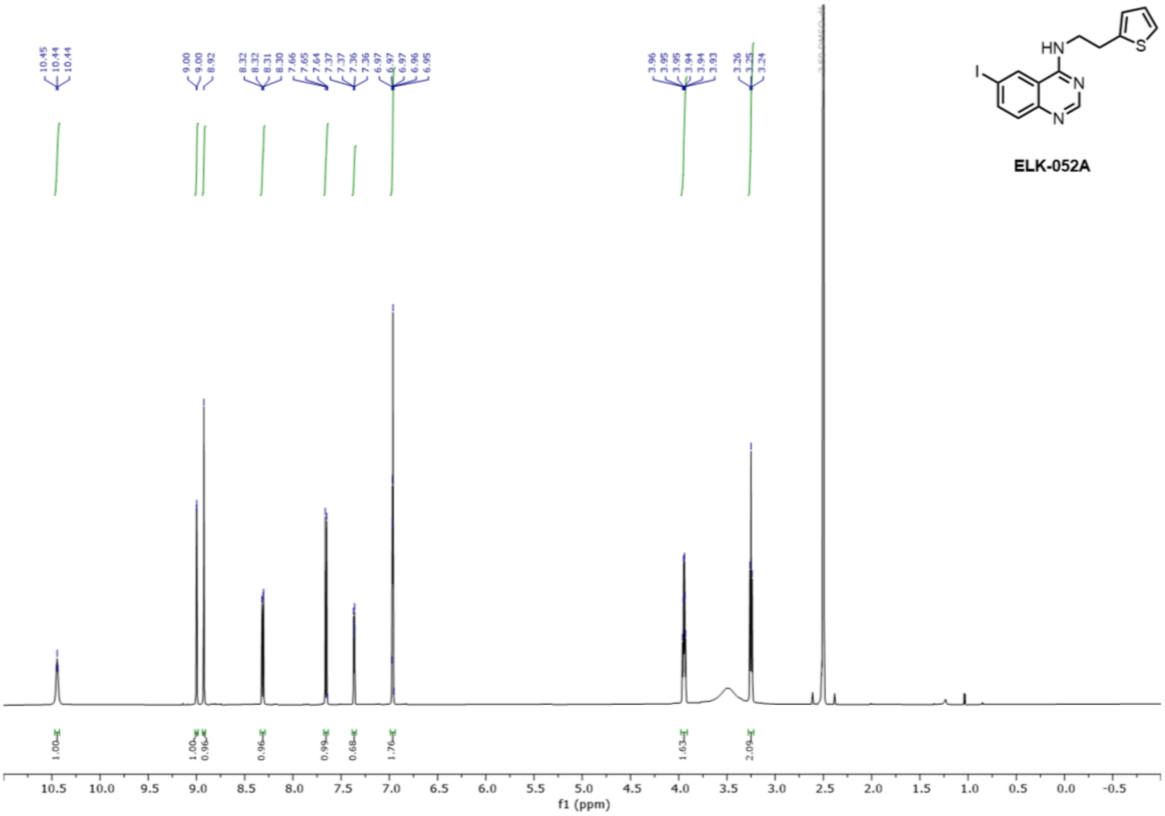

ELK-052A – ^{13}C NMR (151 MHz, DMSO-d_6)

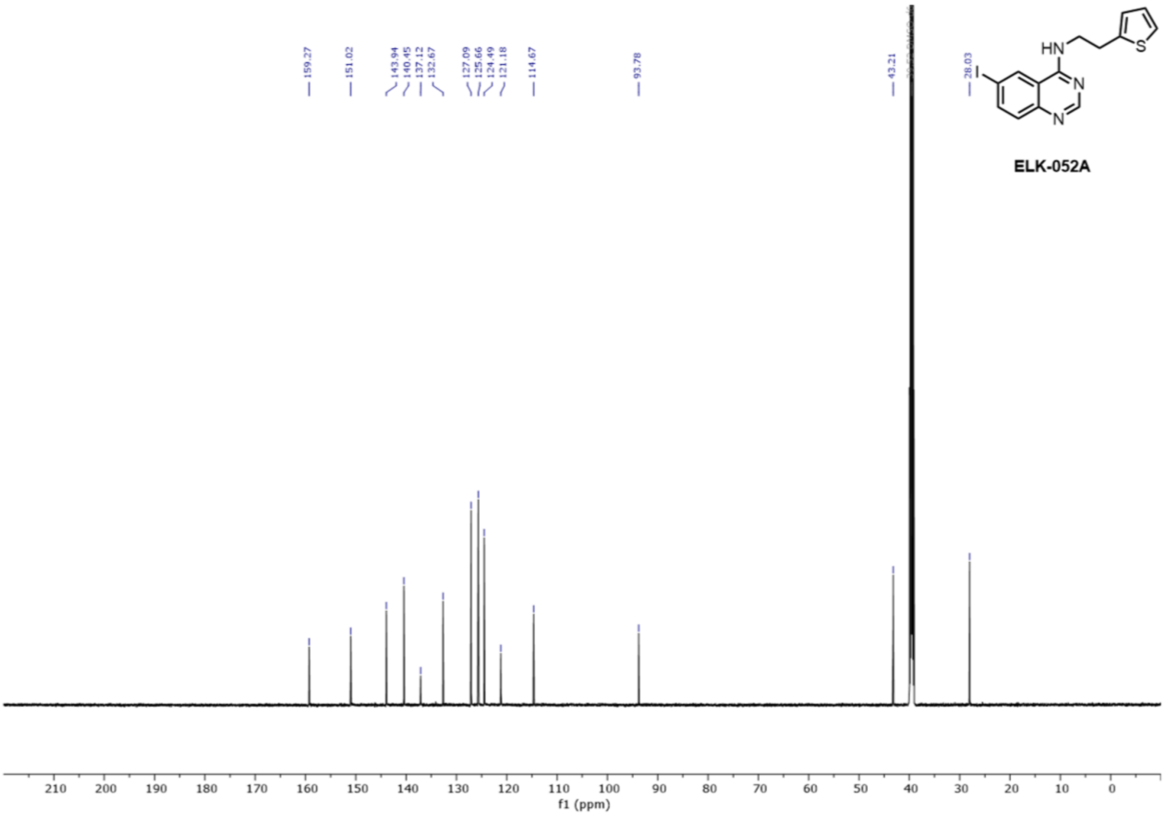

References

1. Rohrbach, S. *et al.* Digitization and validation of a chemical synthesis literature database in the ChemPU. *Science* **377**, 172–180 (2022).
2. Shen, Z. *et al.* An efficient HCCP-mediated direct amination of quinazolin-4(3H)-ones. *Tetrahedron* **67**, 1665–1672 (2011).
3. Whitehead, C. E., Leopold, J. S. & Ziemke, E. Combination with checkpoint inhibitors to treat cancer.
4. Zhou, S. *et al.* Design, synthesis, and structure-activity relationships of a novel class of quinazoline derivatives as coronavirus inhibitors. *European Journal of Medicinal Chemistry* **261**, 115831 (2023).
5. Yang, S. H. *et al.* Virtual screening and synthesis of quinazolines as novel JAK2 inhibitors. *Bioorganic & Medicinal Chemistry* **19**, 968–977 (2011).
6. Long, Q.-S. *et al.* Fabrication of Furan-Functionalized Quinazoline Hybrids: Their Antibacterial Evaluation, Quantitative Proteomics, and Induced Phytopathogen Morphological Variation Studies. *J. Agric. Food Chem.* **67**, 11005–11017 (2019).
7. Zou, Y. *et al.* Fragment-Based Anti-inflammatory Agent Design and Target Identification: Discovery of AF-45 as an IRAK4 Inhibitor to Treat Ulcerative Colitis and Acute Lung Injury. *J. Med. Chem.* **67**, 10687–10709 (2024).
