# Supplementary material for: Chemical programming of kinase inhibitors in a modular chemputer-based system
Source: Commun Biol. 2026 Mar 27;9:874. doi: 10.1038/s42003-026-09873-8 (PMC13315788; doi:10.1038/s42003-026-09873-8)
Supplement: Supplementary file 3 — Description of Additional Supplementary Files [file 42003_2026_9873_MOESM3_ESM.pdf]

Description of Additional Supplementary files

File name: Supplementary Data 1

Description: Provides kinase inhibition activity for AP4-43 and AP2-83, including comparing the two

File name: Supplementary Data 2

Description: Provides raw data for screens
