# Supplementary material for: Chemical programming of kinase inhibitors in a modular chemputer-based system
Source: Commun Biol. 2026 Mar 27;9:874. doi: 10.1038/s42003-026-09873-8 (PMC13315788; doi:10.1038/s42003-026-09873-8)
Supplement: Supplementary file 1 — Transparent Peer Review file [file 42003_2026_9873_MOESM1_ESM.pdf]

nature portfolio

Peer Review File

Chemical Programming of Kinase Inhibitors in a Modular Chemputer-Based System

Corresponding Author: Professor Ross Cagan

This file contains all reviewer reports in order by version, followed by all author rebuttals in order by version.

Version 0:

Reviewer comments:

Reviewer #1

(Remarks to the Author)
The manuscript titled "Chemical Programming of Kinase Inhibitors in a Modular Chemputer-Based System" describes the application of a digital synthesis platform toward the identification and validation of kinase inhibitors. While the core idea is interesting, the manuscript is missing a lot of details which makes it difficult to recommend for publication in its current format. The following are some major concerns that should be addressed before publication:
1. Compound-Specific Synthesis Protocols: While the platform itself has been previously published the methodology is not clear on a) The specific hardware and software configurations employed to generate the compounds. B) Reaction conditions, yields, and any optimizations made C) Essential Chemical Characterization: HNMR, CNMR, HPLC to prove the purity and characterize the compounds. This characterization is a basic requirement for all chemicals to prove their validity.
2. The Kinase profiling assay is described in insufficient details to allow for reproducibility.
3. Single dose is not sufficient to determine if the compounds significantly modulate the target protein. Dose response should be carried out to determine the potency.

Reviewer #2

(Remarks to the Author)
The authors utilized the automated modular synthesis platform "Chemputer", phenotypic screening in a transgenic Drosophila model, and iterative chemical optimization to identify and optimize the compound AP4-43 for targeting KRAS-mutant colorectal cancer (K-CRC), and identified key targets of the compound. The study demonstrates the Chemputer's ability to streamline the synthesis of small molecules and link chemical design directly to biological outcomes, while emphasizing the potential of combining automated synthesis with phenotypic screening to accelerate the discovery of multi-kinase inhibitors for complex, genetically diverse cancers.
It is recommended that the authors further improve and supplement the content and experimental results of the manuscript in the following aspects:
1) The first sentence of the abstract, "The manual synthesis of small molecules presents a bottleneck in medicinal chemistry" is overly definitive. The authors should revise it to "rate-limiting step" or use a more precise expression.
2) The figure or figure legend of Figure 2B should indicate the application concentrations of compounds.
3) Figure 4C should be referenced in the main text in relation to the corresponding experimental results to ensure consistency between the figures and the manuscript content.
4) The article mentions the use of Suzuki coupling to modify the 6-bromo position of the 4-chloroquinazoline core, but it seems that the subsequent compounds (such as AP2-83, AP4-43) do not mention the modification or optimization using the Suzuki reaction. Has the Suzuki reaction optimization been considered to further improve the activity of AP4-43? If such data exists, would the authors be able to supplement this information in the manuscript?

Reviewer #3

(Remarks to the Author)
This study explores the use of an automated digital synthesis platform ('Chemputer') for drug discovery targeting KRAS-mutant colorectal cancer (CRC). While the authors synthesized a library of 4-anilinoquinazoline compounds and identified

AP2-83 through a Drosophila CRC model, the biological validation of the hits appears limited. Although AP4-43 showed improved efficacy in both Drosophila and mammalian organoids, the mechanistic characterization remains insufficient, particularly regarding downstream signaling and target engagement in relevant cellular contexts. Without more rigorous functional assays, it is difficult to assess the true therapeutic potential of these compounds. Overall, the concept is promising, but additional experimental evidence is necessary to convincingly support the authors' conclusions.

Major Comments

1. The overall flow of the manuscript is somewhat confusing, as the description of Figure 2 precedes that of Figure 1 in the Results section. Reorganizing the text to follow the numerical order of figures would enhance readability and improve the logical progression of the results, making the manuscript more persuasive.

2. Although the authors performed multi-kinase profiling with the hit compound AP2-83, the inhibitory effects should be further validated in relevant cellular models, such as organoids or cell lines. For instance, since AP2-82 is suggested to inhibit PI3K and CLK1, downstream effectors including phospho-AKT (a key mediator of PI3K signaling) and phospho-SR proteins (targets of CLK1) should be evaluated to confirm target engagement in a biological context. Such validation would strengthen the mechanistic conclusions.

3. In Figures 4A-B and 5D, organoid diameters were measured to assess growth inhibition. While these data provide preliminary insights, they are insufficient to conclusively demonstrate inhibitory effects. Furthermore, the selectivity of the hit compounds between cancerous and normal cells remains unaddressed. I recommend incorporating complementary assays, such as apoptosis or other cell death markers, to more rigorously evaluate both efficacy and selectivity.

4. The manuscript reports that "In the AKP mouse CRC organoid model, AP4-43 (2 µM) was more effective at reducing organoid growth than both AP2-83 and the standard-of-care drug regorafenib at the same concentration (Fig. 5D)." However, in Figure 5D, the inhibitory effect of AP4-43 appears lower than that of AP2-83, the earlier version of the compound. The authors should clarify this apparent discrepancy to avoid confusion and ensure accurate interpretation of the results.

Minor Comments

• Figure 4C is not mentioned or discussed in the manuscript. The authors should provide a brief description and interpretation of this panel in the Results section.

Version 1:

Reviewer comments:

Reviewer #1

(Remarks to the Author)
The authors have addressed all comments

Reviewer #2

(Remarks to the Author)
The authors have addressed my questions and concerns.

Reviewer #3

(Remarks to the Author)
The authors have addressed all my initial concerns. Thanks.

Open Access This Peer Review File is licensed under a Creative Commons Attribution 4.0 International License, which permits use, sharing, adaptation, distribution and reproduction in any medium or format, as long as you give appropriate credit to the original author(s) and the source, provide a link to the Creative Commons license, and indicate if changes were made.
In cases where reviewers are anonymous, credit should be given to 'Anonymous Referee' and the source.
The images or other third party material in this Peer Review File are included in the article's Creative Commons license, unless indicated otherwise in a credit line to the material. If material is not included in the article's Creative Commons license and your intended use is not permitted by statutory regulation or exceeds the permitted use, you will need to obtain permission directly from the copyright holder.
To view a copy of this license, visit https://creativecommons.org/licenses/by/4.0/

Chemical Programming of Kinase Inhibitors in a Modular Chemputer-Based System:
Response to Reviewers

Reviewer #1 (Remarks to the Author):

The manuscript titled "Chemical Programming of Kinase Inhibitors in a Modular Chemputer-Based System" describes the application of a digital synthesis platform toward the identification and validation of kinase inhibitors. While the core idea is interesting, the manuscript is missing a lot of details which makes it difficult to recommend for publication in its current format. The following are some major concerns that should be addressed before publication:

1. Compound-Specific Synthesis Protocols: While the platform itself has been previously published the methodology is not clear on a) The specific hardware and software configurations employed to generate the compounds. B) Reaction conditions, yields, and any optimizations made C) Essential Chemical Characterization: HNMR, CNMR, HPLC to prove the purity and characterize the compounds. This characterization is a basic requirement for all chemicals to prove their validity.

We now provide significantly more detailed methods in the Methods section, and now include a detailed Appendix that describes the automated synthesis platform configuration, experimental details of specific compounds, and NMR spectra.

2. The Kinase profiling assay is described in insufficient details to allow for reproducibility.

We now provide a detailed kinase profiling protocol in the Methods section.

3. Single dose is not sufficient to determine if the compounds significantly modulate the target protein. Dose response should be carried out to determine the potency.

We now provide dose response curves of 1, 10, and 100 µM for each compound (Fig. 5C and Extended Fig. 2).

Reviewer #2 (Remarks to the Author):

The authors utilized the automated modular synthesis platform "Chemputer", phenotypic screening in a transgenic Drosophila model, and iterative chemical optimization to identify and optimize the compound AP4-43 for targeting KRAS-mutant colorectal cancer (K-CRC), and identified key targets of the compound. The study demonstrates the Chemputer's ability to streamline the synthesis of small molecules and link chemical design directly to biological outcomes, while emphasizing the potential of combining automated synthesis with phenotypic screening to accelerate the discovery of multi-kinase inhibitors for complex, genetically diverse cancers.
It is recommended that the authors further improve and supplement the content and experimental results of the manuscript in the following aspects:
1) The first sentence of the abstract, "The manual synthesis of small molecules presents a bottleneck in medicinal chemistry" is overly definitive. The authors should revise it to "rate-limiting step" or use a more precise expression.

We agree and have changed the first sentence to "Manual synthesis of small molecules can represent a rate-limiting step in medicinal chemistry."

2) The figure or figure legend of Figure 2B should indicate the application concentrations of compounds.

The figure legend (for Figure 3B) now states that "All compounds tested by feeding at 10 μM final food concentration".

3) Figure 4C should be referenced in the main text in relation to the corresponding experimental results to ensure consistency between the figures and the manuscript content.

Thank you for catching this. The relevant sentence in the Results section now reads: "Reducing the gene dosage of the fly orthologues of CLK1 ((*byn>RAP-p1; dyrk3*$^{-/+}$) and PI3K (*byn>RAP-p1;pi3k68D*$^{+/-}$) significantly decreased HPZ area and increased animal survival compared to flies treated with low dose AP2-83 alone (Fig. 4C-D).".

4) The article mentions the use of Suzuki coupling to modify the 6-bromo position of the 4-chloroquinazoline core, but it seems that the subsequent compounds (such as AP2-83, AP4-43) do not mention the modification or optimization using the Suzuki reaction. Has the Suzuki reaction optimization been considered to further improve the activity of AP4-43? If such data exists, would the authors be able to supplement this information in the manuscript?

We did explore this modification strategy in our SAR studies. However, our systematic investigation found that introducing aryl substituents at the 6-position *via* Suzuki coupling consistently resulted in reduced biological activity. This was observed in multiple analogues including the conversion of AP2-81 to AP2-84, AP2-83 to AP2-86, and AP2-83 to AP2-89, where replacing the 6-bromo with phenyl groups diminished potency.

Based on our consistent findings that the 6-bromo substituent is optimal for biological activity in this scaffold, we did not pursue further Suzuki coupling optimization for AP4-43. The data strongly suggests that the 6-bromo moiety represents a key pharmacophoric element for maintaining potent kinase inhibition in this quinazoline series.

Reviewer #3 (Remarks to the Author):

This study explores the use of an automated digital synthesis platform ('Chemputer') for drug discovery targeting KRAS-mutant colorectal cancer (CRC). While the authors synthesized a library of 4-anilinoquinazoline compounds and identified AP2-83 through a Drosophila CRC model, the biological validation of the hits appears limited. Although AP4-43 showed improved efficacy in both Drosophila and mammalian organoids, the mechanistic characterization remains insufficient, particularly regarding downstream signaling and target engagement in relevant cellular contexts. Without more rigorous functional assays, it is difficult to assess the true therapeutic potential of these compounds. Overall, the concept is promising, but additional experimental evidence is necessary to convincingly support the authors' conclusions.

Major Comments

1. The overall flow of the manuscript is somewhat confusing, as the description of Figure 2 precedes that of Figure 1 in the Results section. Reorganizing the text to follow the numerical order of figures would enhance readability and improve the logical progression of the results, making the manuscript more persuasive.

We agree and have now (i) reorganised the text to better match the order of the figures, and (ii) improved and clarified the text describing our approach in the first section of the Results. We have also added a Fig. 8 that summarises the development of AP4-43.

2. Although the authors performed multi-kinase profiling with the hit compound AP2-83, the inhibitory effects should be further validated in relevant cellular models, such as organoids or cell lines. For instance, since AP2-82 is suggested to inhibit PI3K and CLK1, downstream effectors including phospho-AKT (a key mediator of PI3K signaling) and phospho-SR proteins (targets of CLK1) should be evaluated to confirm target engagement in a biological context. Such validation would strengthen the mechanistic conclusions.

We now provide new Fig. 7 with AKP organoid data that demonstrates significant reduction in two CLK cancer targets: ß-catenin (CLK-1) and NF-κB (CLK-4; Fig. 7A-H). These mirror results from other inhibitors of CLK-1 and CLK-4, also referenced in the text.

We did not observe changes in phospho-AKT (Fig. 7I-L); however as discussed in the text, in CRC (i) PIK3CG often acts as a tumour suppressor (and is commonly downregulated in CRC, although its levels in *AKP* cells has not been reported) or (ii) can act as an oncogene but perhaps redundant to PIK3CA (references provided in text). Also of note, Drosophila has a single orthologue for both.

Finally, we now also include data demonstrating that neither AP2-83 nor AP4-43 impact canonical RAS/MAPK signalling, as assessed by phosphorylated ERK (Fig. 7M-P). This is consistent with both molecules' profiles and further suggests an alternative approach to targeting KRAS-driven CRC.

The Methods section has been expanded to reflect this new work.

3. In Figures 4A-B and 5D, organoid diameters were measured to assess growth inhibition. While these data provide preliminary insights, they are insufficient to conclusively

demonstrate inhibitory effects. Furthermore, the selectivity of the hit compounds between cancerous and normal cells remains unaddressed. I recommend incorporating complementary assays, such as apoptosis or other cell death markers, to more rigorously evaluate both efficacy and selectivity.

We now provide new data showing IC50s for AP2-83 and AP4-43 in human K-CRC cell lines SW620 and SW837 treated for 72 hrs. Both compounds showed tumour cell killing in the low µM range (Fig. 5D, 5E). The Methods section has been expanded to reflect this new work.

4. The manuscript reports that "In the AKP mouse CRC organoid model, AP4-43 (2 µM) was more effective at reducing organoid growth than both AP2-83 and the standard-of-care drug regorafenib at the same concentration (Fig. 5D)." However, in Figure 5D, the inhibitory effect of AP4-43 appears lower than that of AP2-83, the earlier version of the compound. The authors should clarify this apparent discrepancy to avoid confusion and ensure accurate interpretation of the results.

Thank you for pointing this out: it was an error on our part. We have re-written this: "In *AKP* mouse CRC organoids, AP4-43 (2 µM) was more effective at reducing organoid growth than the standard-of-care drug regorafenib at the same concentration (Fig. 5F), though less effective than AP2-83 or AP4-42." In the discussion we now include the point: "Also of note, AP2-83 showed better activity in mouse organoids, either reflecting (i) differences in whole animal vs. cultured cells, such as toxicity, or (ii) differences between mouse and fly."

Minor Comments

• Figure 4C is not mentioned or discussed in the manuscript. The authors should provide a brief description and interpretation of this panel in the Results section.

Thank you, we have corrected this: "Reducing the gene dosage of the fly orthologues of CLK1 ((*byn>RAP-p1; dyrk3*$^{-/+}$) and PI3K (*byn>RAP-p1;pi3k68D*$^{+/-}$) significantly decreased HPZ area and increased animal survival compared to flies treated with low dose AP2-83 alone (Fig. 4C-D)." We have also checked to make sure that all Figures are properly referenced in the text in their appropriate order.
